# Supplementary material for: Genetic architecture of 67 oral diseases and their links to systemic diseases
Source: HGG Adv. 2026 Jun 17;7(3):100633. doi: 10.1016/j.xhgg.2026.100633 (PMC13343153; doi:10.1016/j.xhgg.2026.100633)
Supplement: Document S1. Figures S1–S71 [file mmc1.pdf]

HGGA, Volume 7

Supplemental information

Genetic architecture of 67 oral diseases and their links to systemic diseases

Kirika Karppinen, Hanna M. Ollila, Kanwal Batool, FinnGen, Estonian Biobank Research Team, Erik Abner, David P. Rice, Aarno Palotie, Tuula Palotie, Samuli Ripatti, Nina Mars, and Satu Strausz

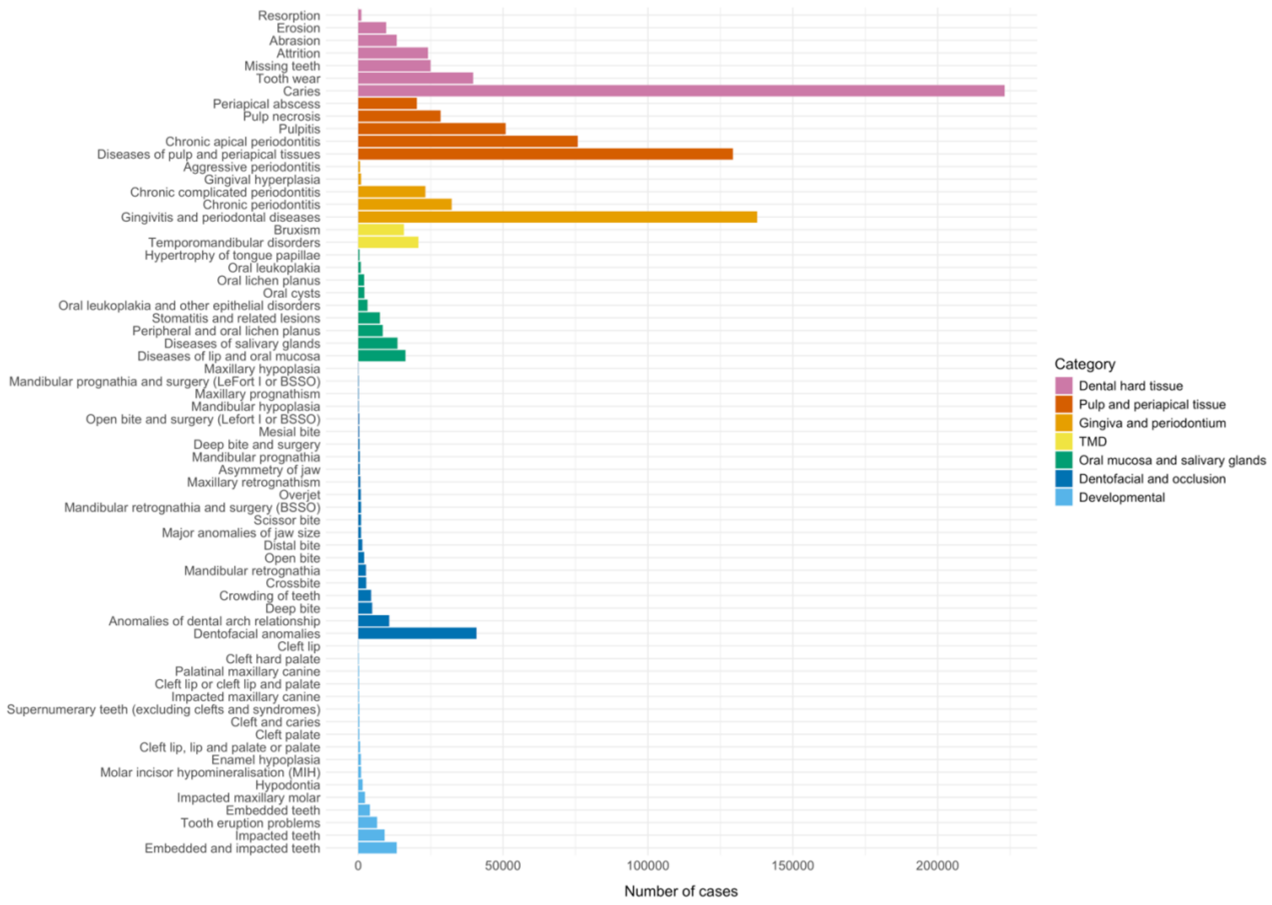

Figure S1. Number of cases of all oral and craniofacial phenotypes. *Phenotypes are organized by phenotype category.*

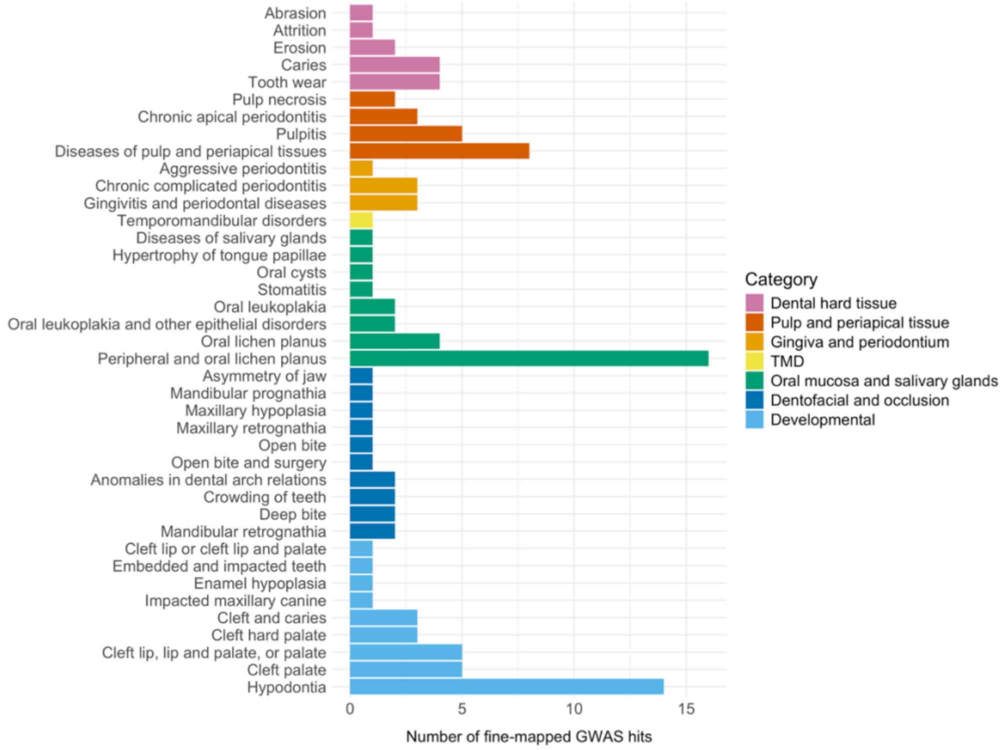

Figure S2. Number of fine-mapped GWAS association per oral and craniofacial phenotype. *Phenotypes are organized by phenotype category.*

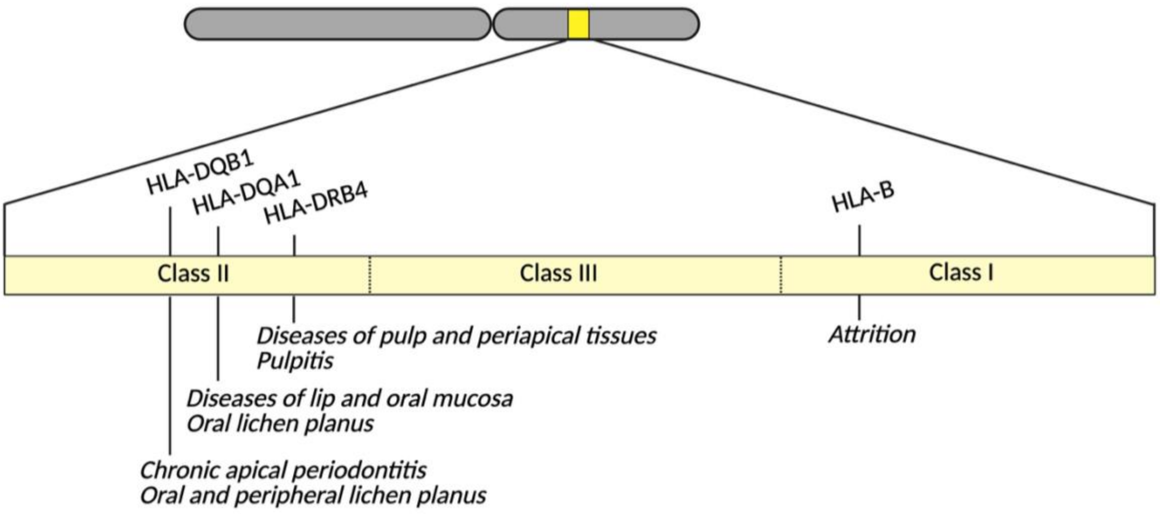

Figure S3. Association of oral phenotypes with HLA alleles according to HLA fine-mapping results. *The approximate positions of the associated HLA alleles within the HLA region are shown. The figure is not drawn to scale.*

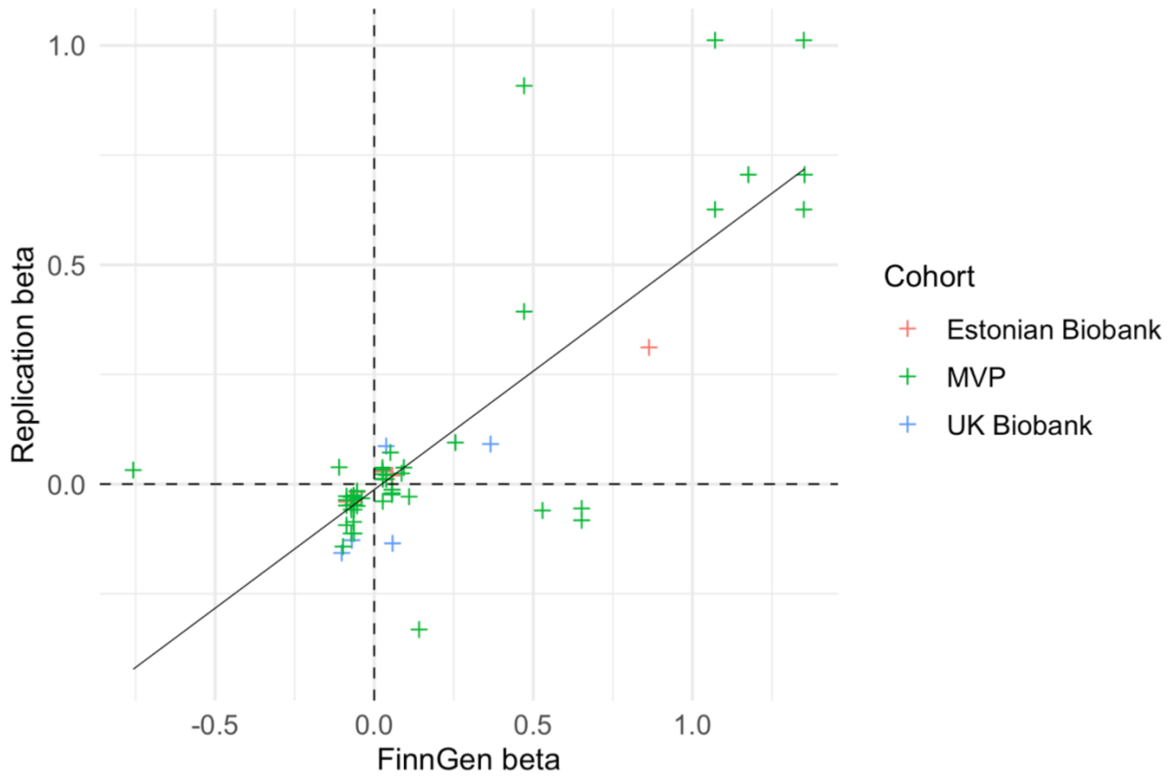

Figure S4. Comparison of effect size estimates between discovery and replication analyses. *Each point represents a successfully replicated variant-phenotype association, with FinnGen GWAS beta estimates plotted against replication estimates across Estonian Biobank, MVP and UK Biobank cohorts. The overall linear fit is shown by the regression line.*

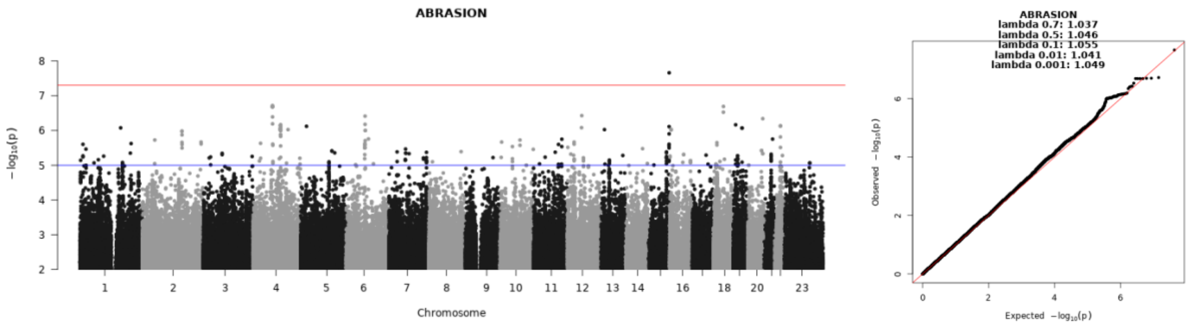

Figure S5. Manhattan and QQ-plot of phenotype Abrasion.

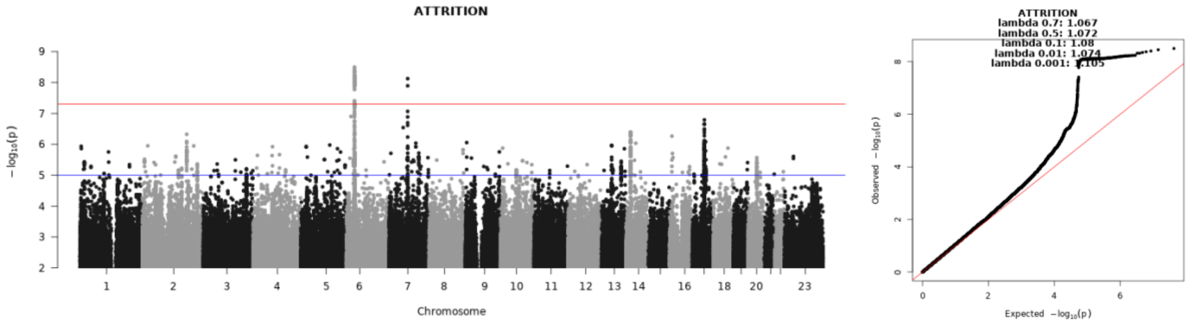

Figure S6. Manhattan and QQ-plot of phenotype Attrition.

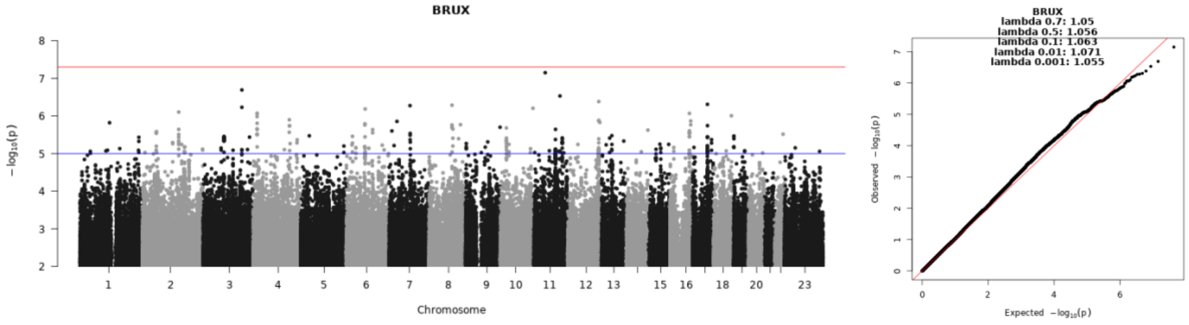

Figure S7. Manhattan and QQ-plot of phenotype Bruxism.

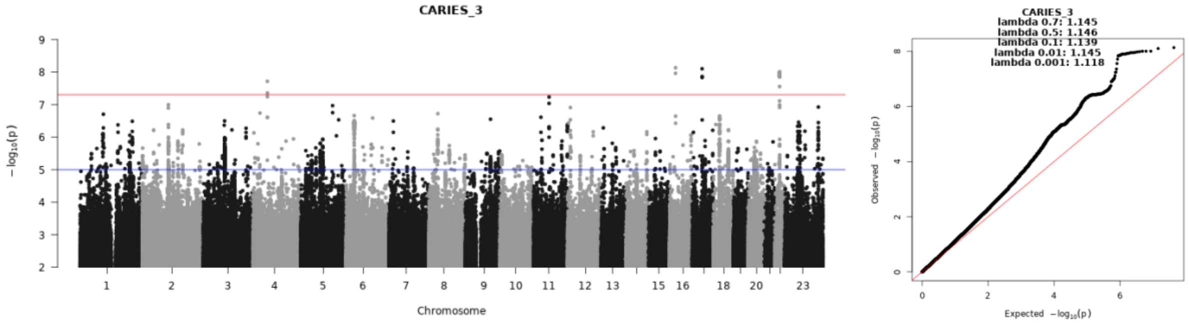

Figure S8. Manhattan and QQ-plot of phenotype Caries.

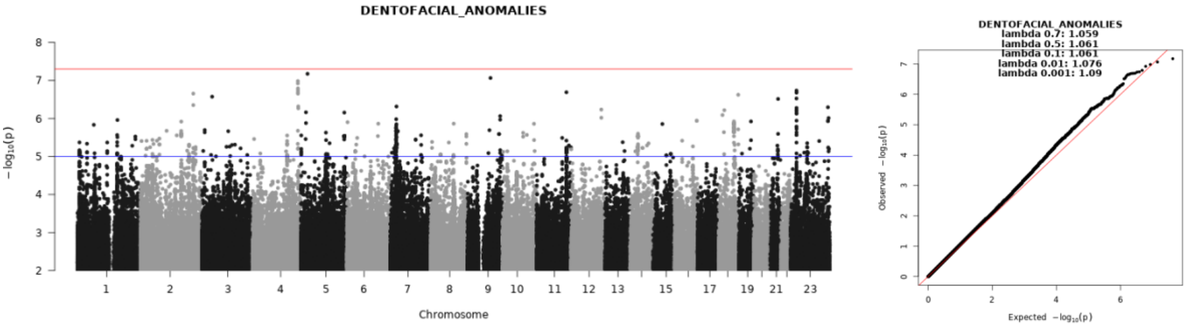

Figure S9. Manhattan and QQ-plot of phenotype Dentofacial anomalies.

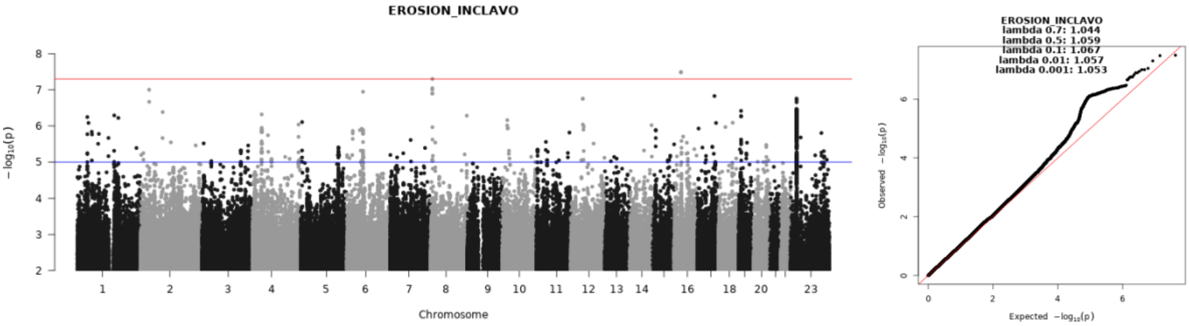

Figure S10. Manhattan and QQ-plot of phenotype Erosion.

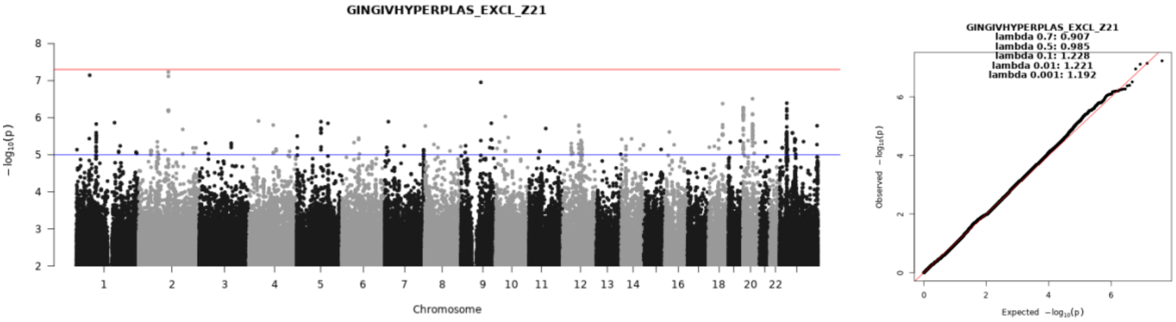

Figure S11. Manhattan and QQ-plot of phenotype Gingival hyperplasia.

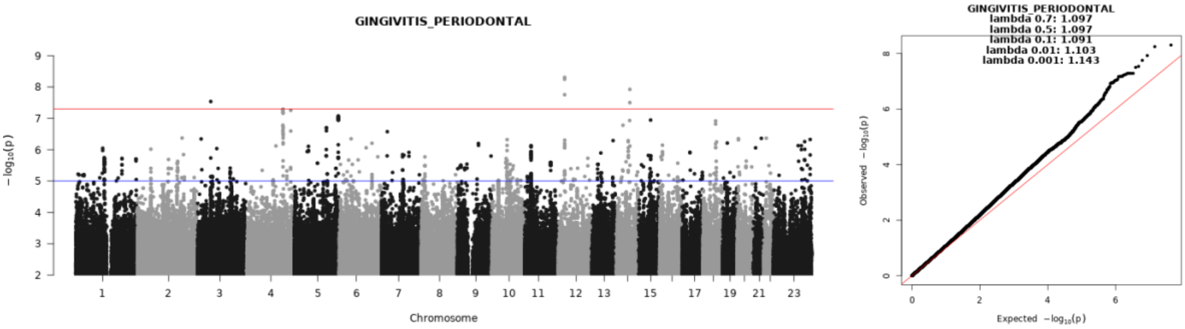

Figure S12. Manhattan and QQ-plot of phenotype Gingivitis and periodontal diseases.

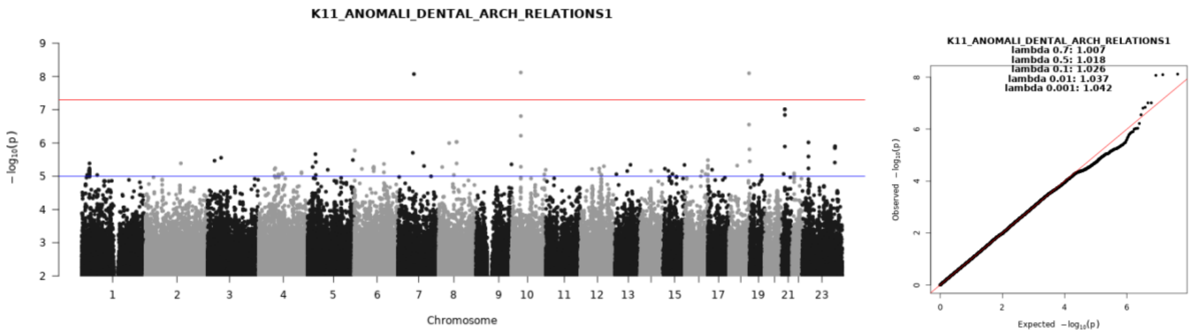

Figure S13. Manhattan and QQ-plot of phenotype Anomalies of dental arch relationship.

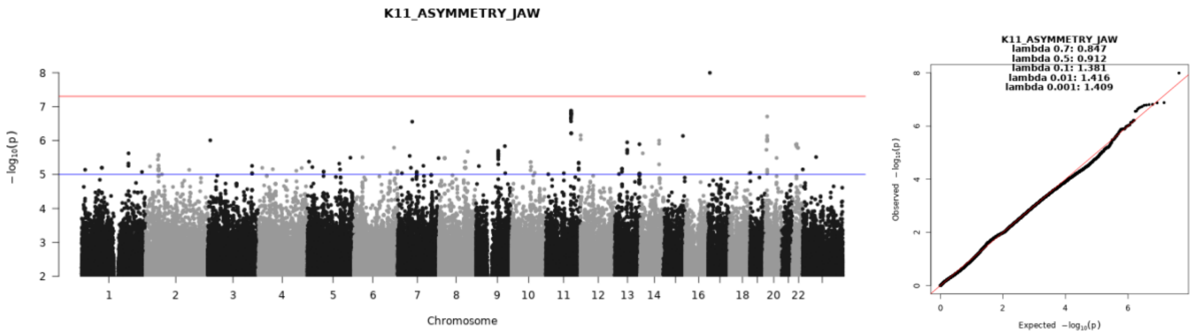

Figure S14. Manhattan and QQ-plot of phenotype Asymmetry of jaw.

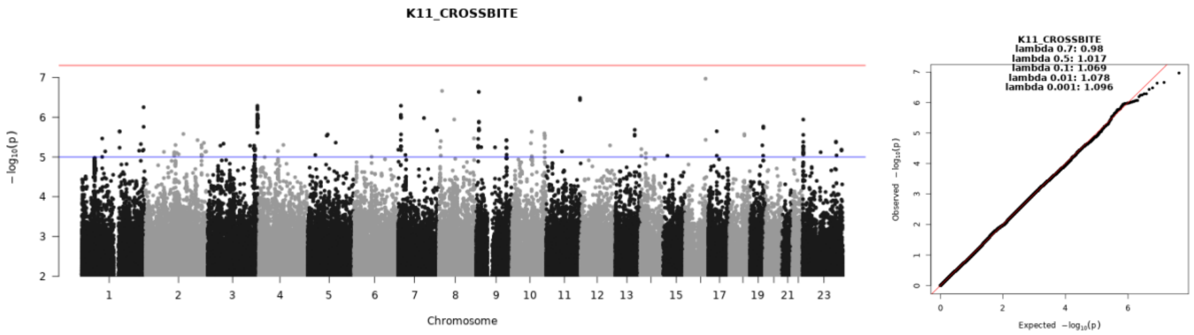

Figure S15. Manhattan and QQ-plot of phenotype Crossbite.

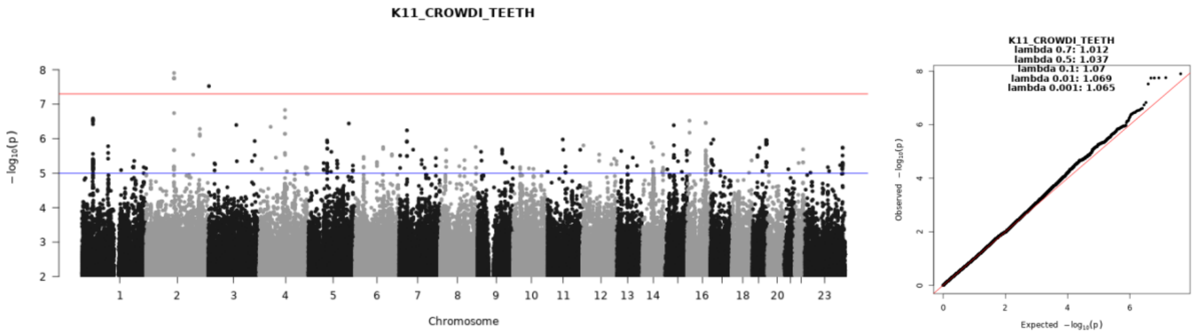

Figure S16. Manhattan and QQ-plot of phenotype Crowding of teeth.

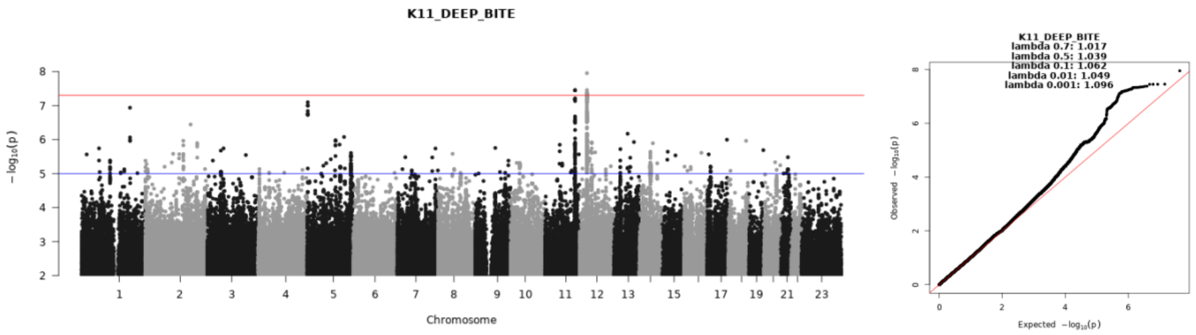

Figure S17. Manhattan and QQ-plot of phenotype Deep bite.

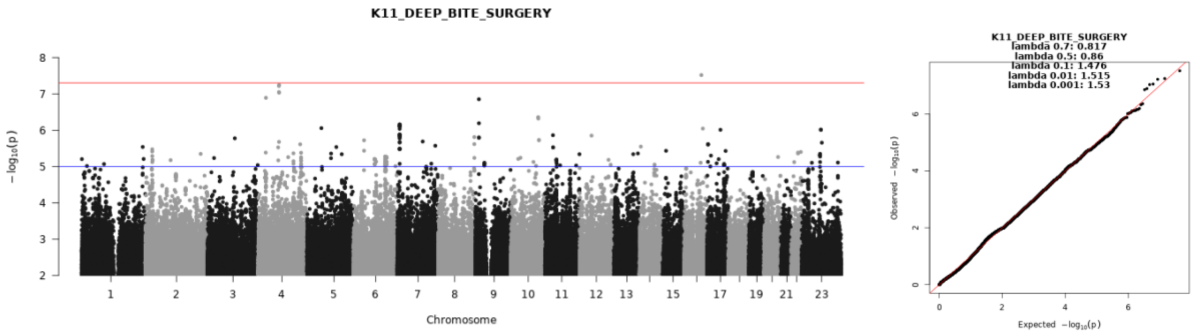

Figure S18. Manhattan and QQ-plot of phenotype Deep bite that required surgery.

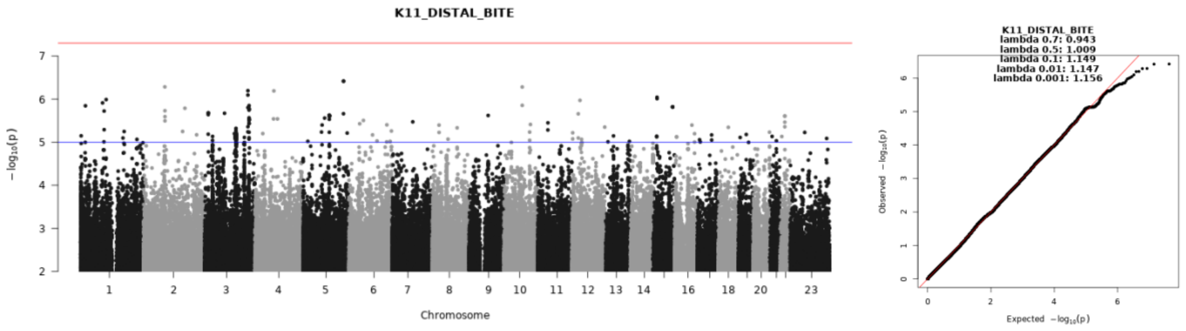

Figure S19. Manhattan and QQ-plot of phenotype Disto-occlusion.

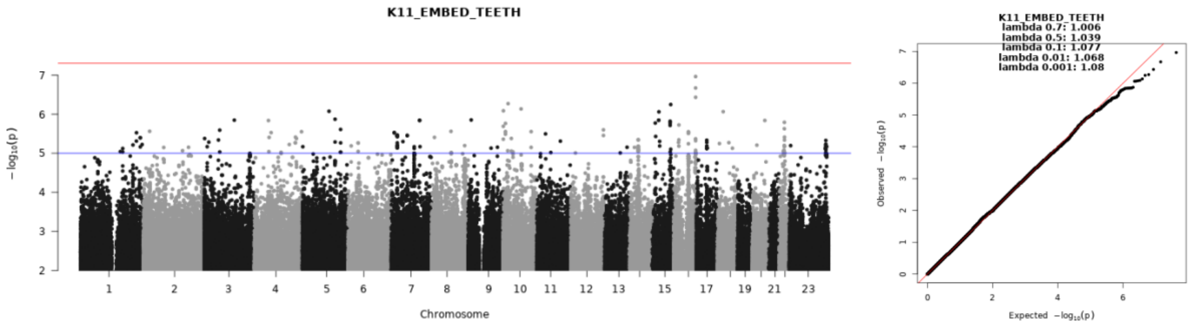

Figure S20. Manhattan and QQ-plot of phenotype Embedded teeth.

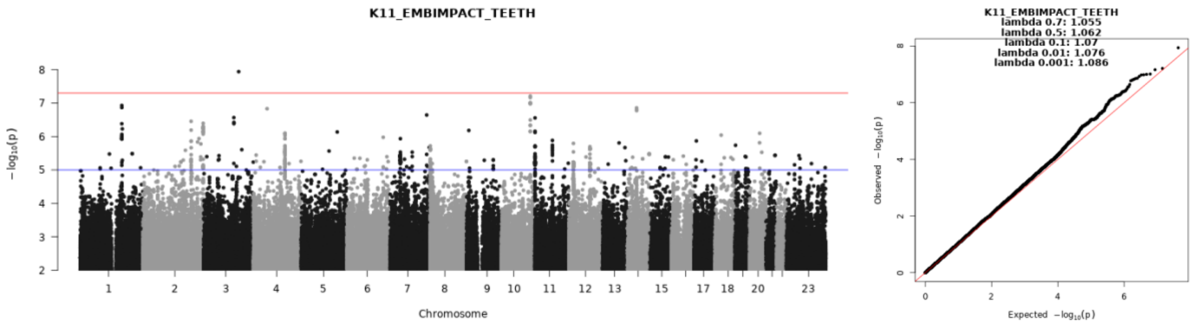

Figure S21. Manhattan and QQ-plot of phenotype Embedded and impacted teeth.

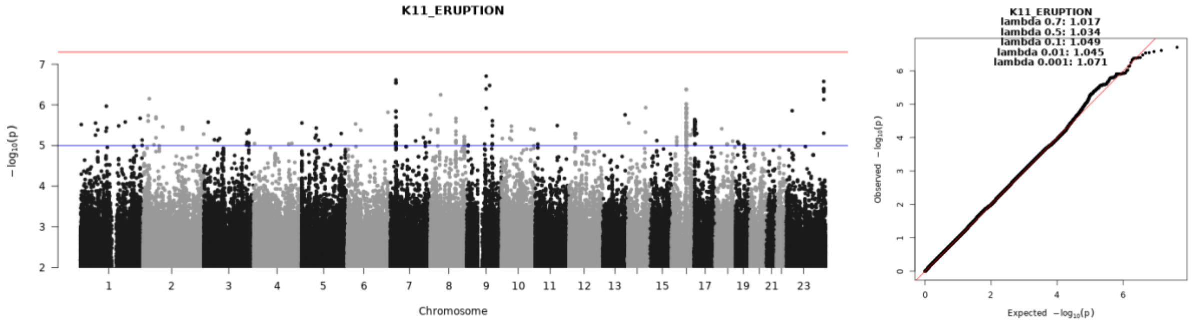

Figure S22. Manhattan and QQ-plot of phenotype Tooth eruption problems.

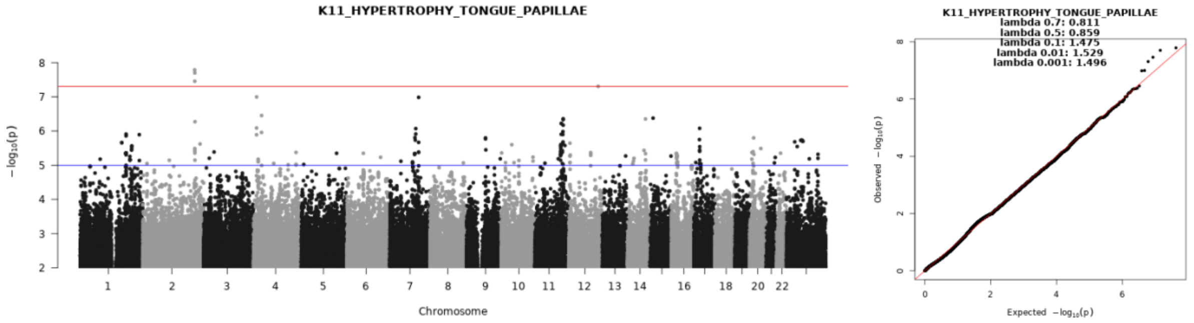

Figure S23. Manhattan and QQ-plot of phenotype Hypertrophy of tongue papillae.

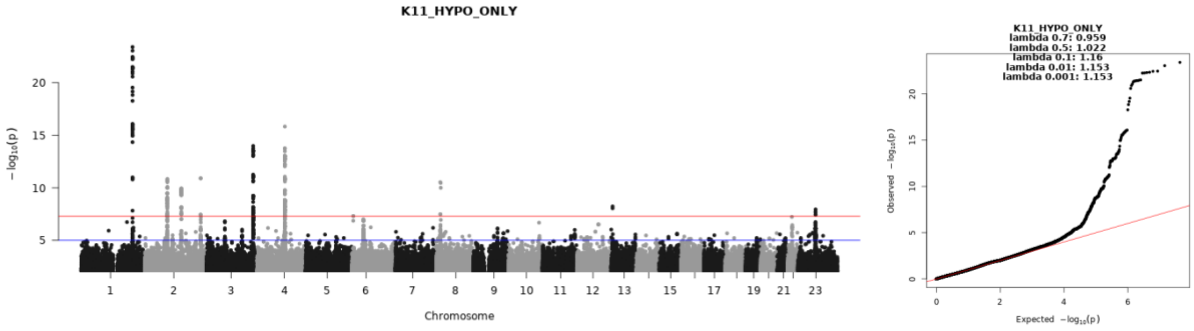

Figure S24. Manhattan and QQ-plot of phenotype Hypodontia or oligodontia.

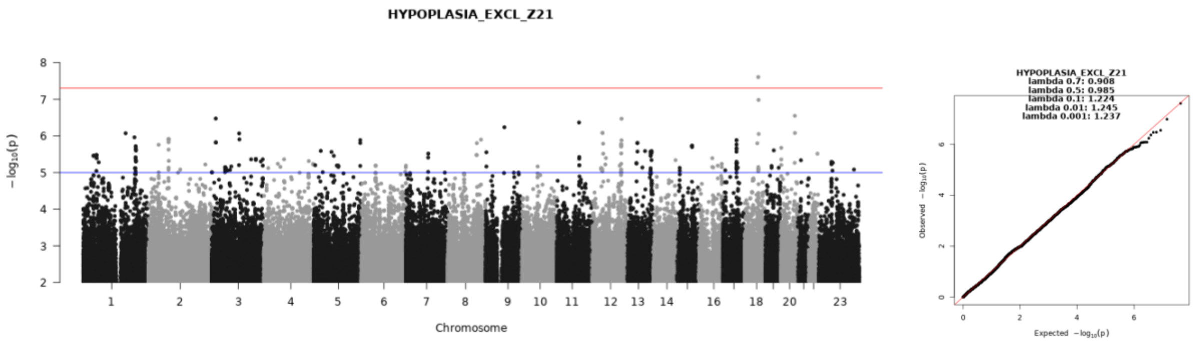

Figure S25. Manhattan and QQ-plot of phenotype Hypoplasia of dental enamel.

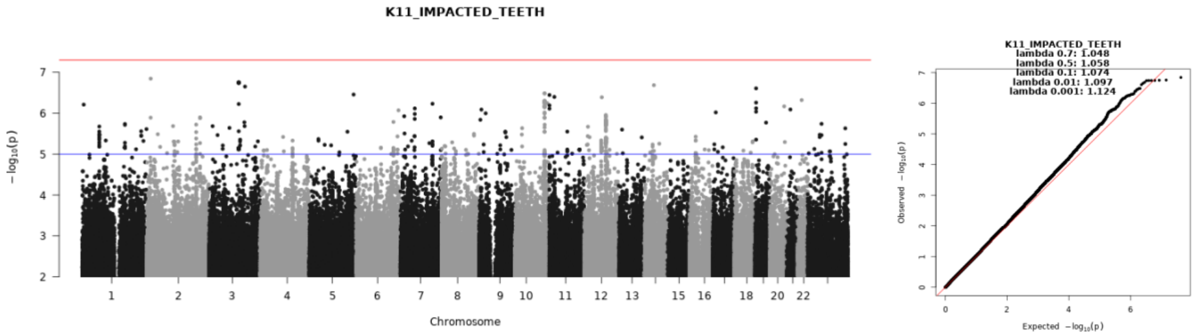

Figure S26. Manhattan and QQ-plot of phenotype Impacted teeth.

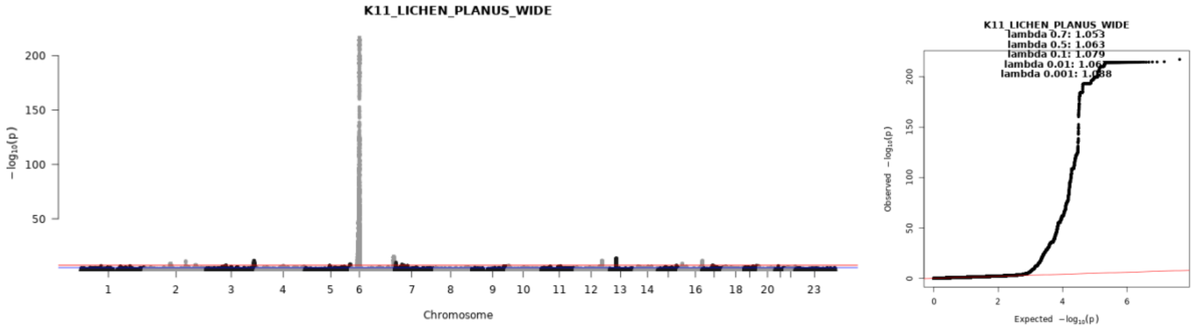

Figure S27. Manhattan and QQ-plot of phenotype Peripheral and oral lichen planus.

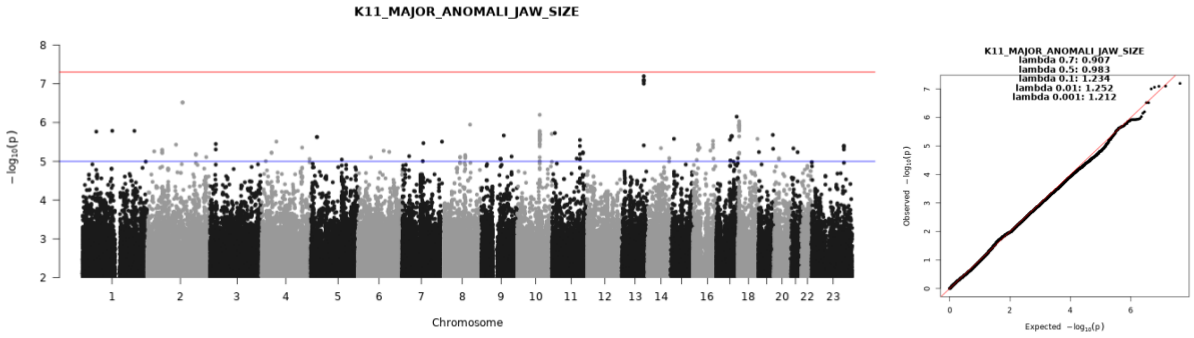

Figure S28. Manhattan and QQ-plot of phenotype Major anomalies of jaw size.

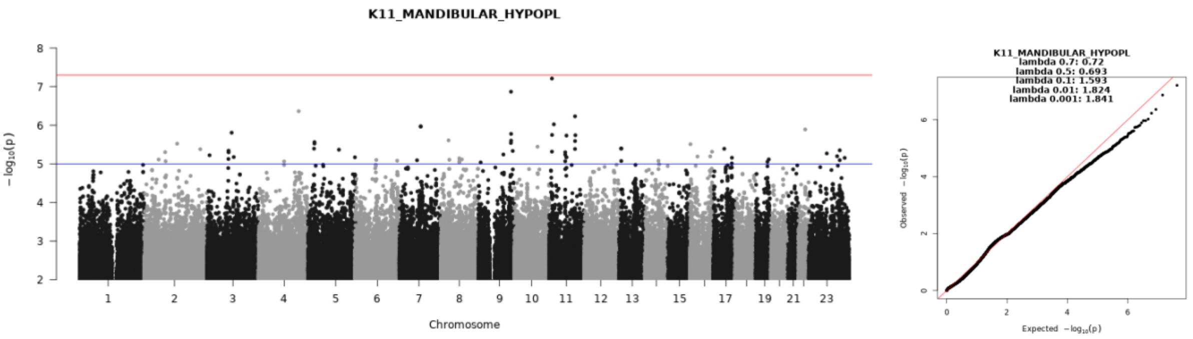

Figure S29. Manhattan and QQ-plot of phenotype Mandibular hypoplasia.

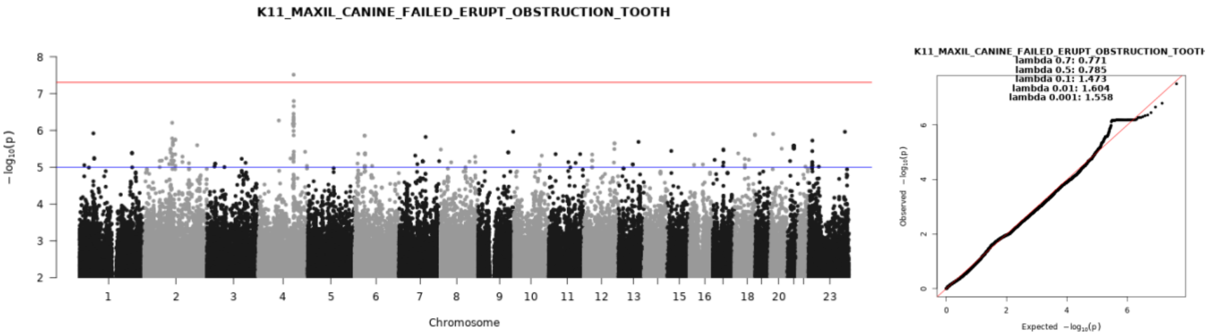

Figure S30. Manhattan and QQ-plot of phenotype Impacted maxillary canine.

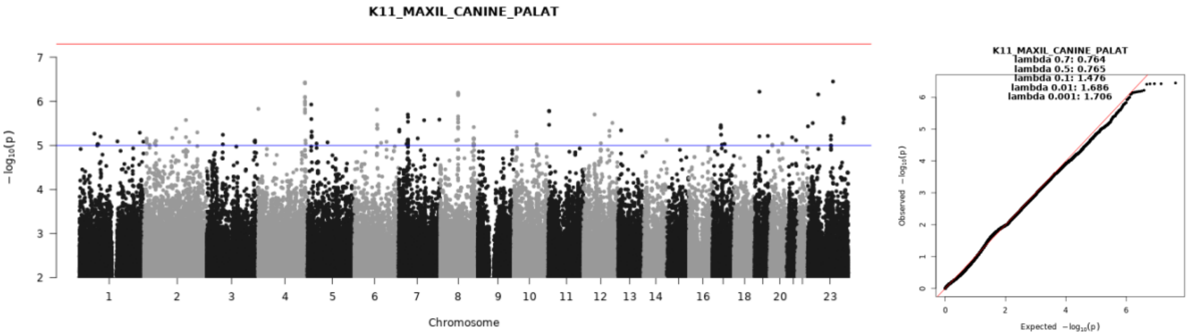

Figure S31. Manhattan and QQ-plot of phenotype Palatinal maxillary canine.

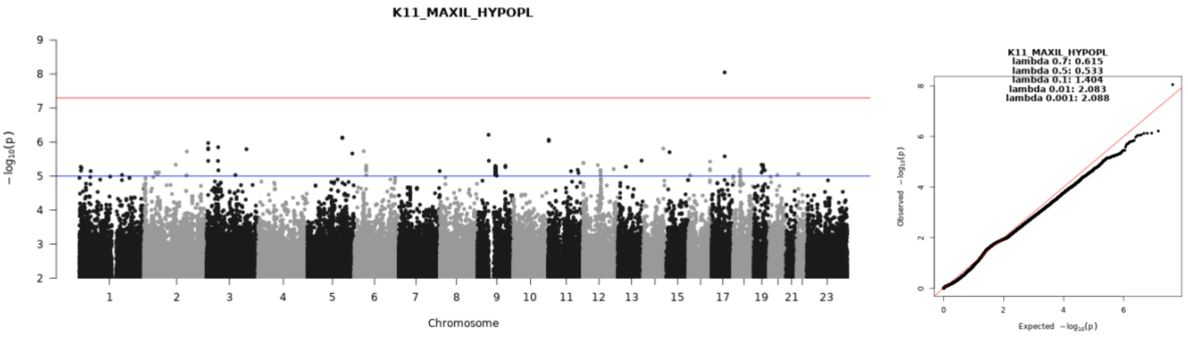

Figure S32. Manhattan and QQ-plot of phenotype Maxillary hypoplasia.

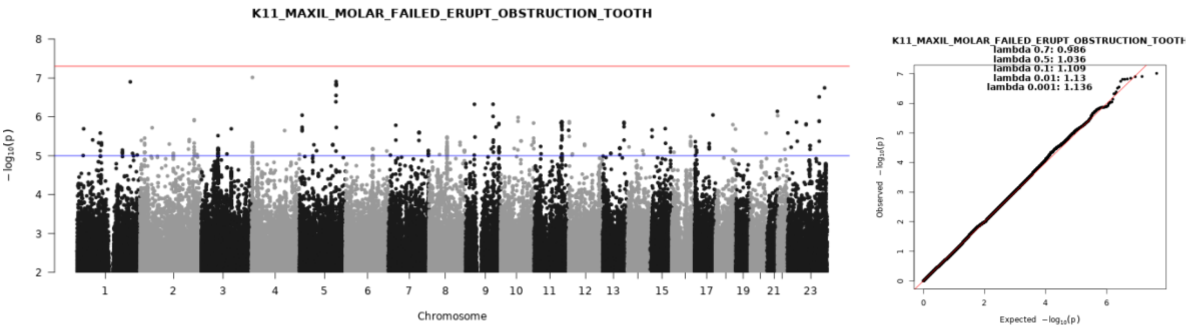

Figure S33. Manhattan and QQ-plot of phenotype Impacted maxillary molar.

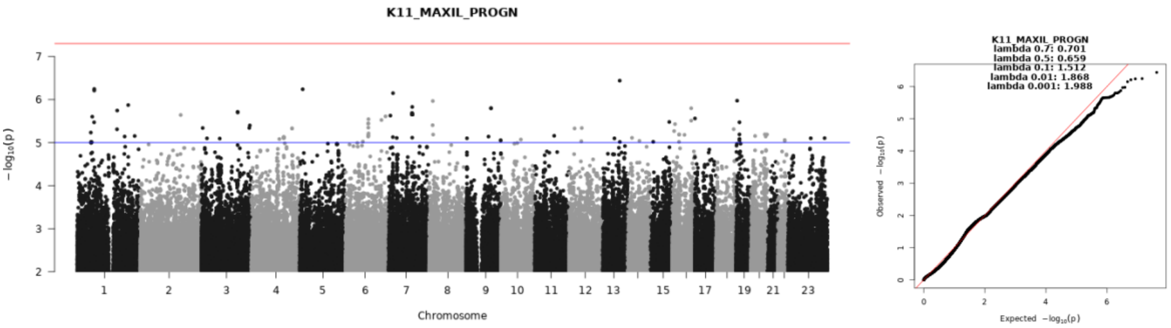

Figure S34. Manhattan and QQ-plot of phenotype Maxillary prognathia.

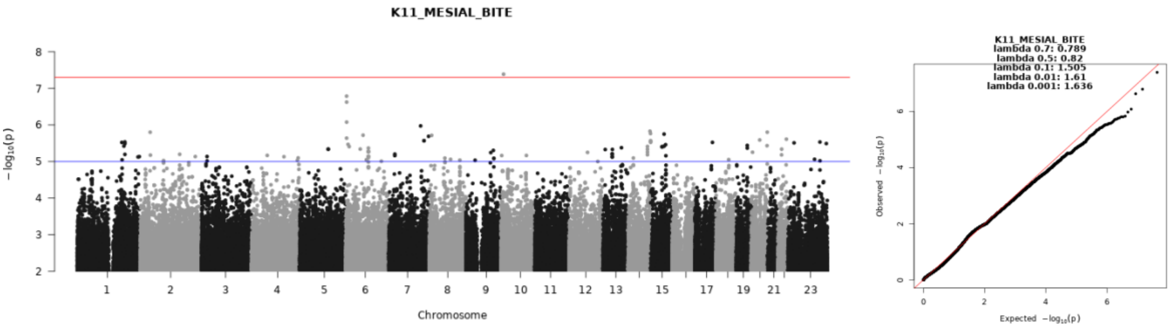

Figure S35. Manhattan and QQ-plot of phenotype Mesio-occlusion.

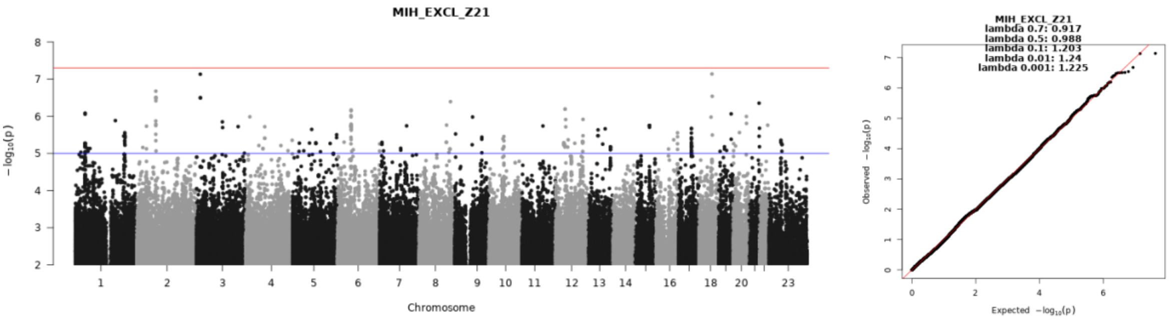

Figure S36. Manhattan and QQ-plot of phenotype Molar incisor hypomineralisation (MIH).

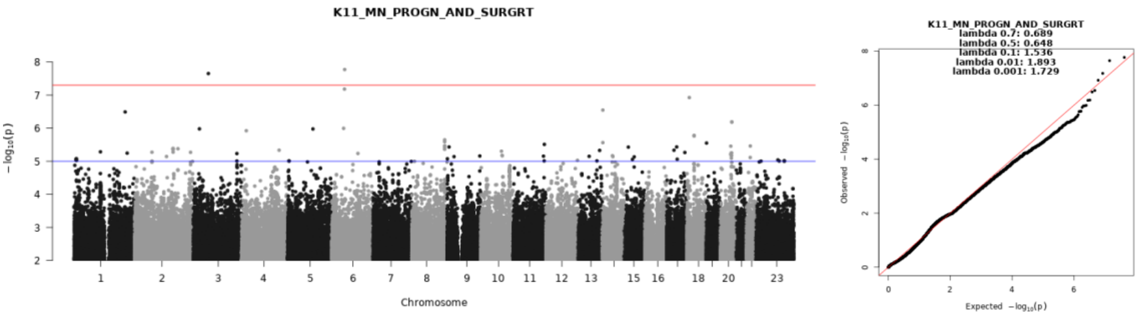

Figure S37. Manhattan and QQ-plot of phenotype Mandibular prognathia and surgery (LeFort I or BSSRO).

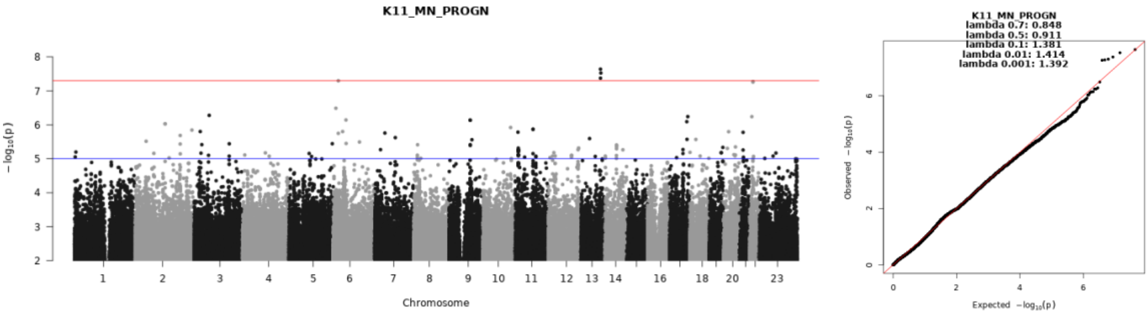

Figure S38. Manhattan and QQ-plot of phenotype Mandibular prognathia.

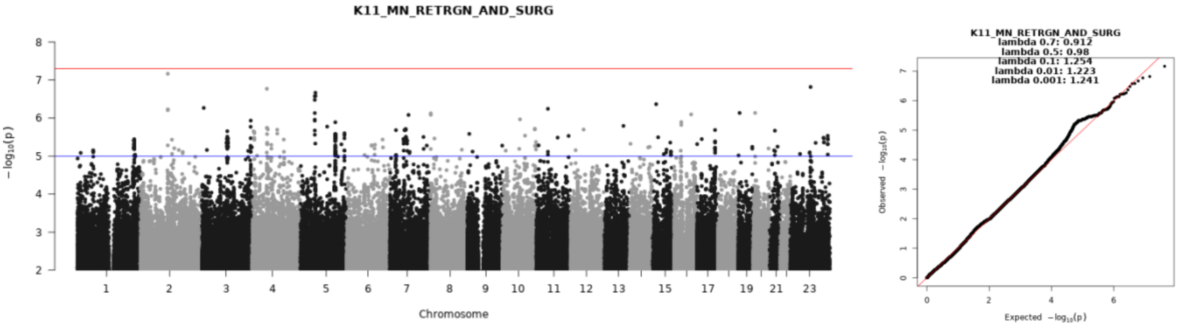

Figure S39. Manhattan and QQ-plot of phenotype Mandibular retrognathia and surgery (BSSRO).

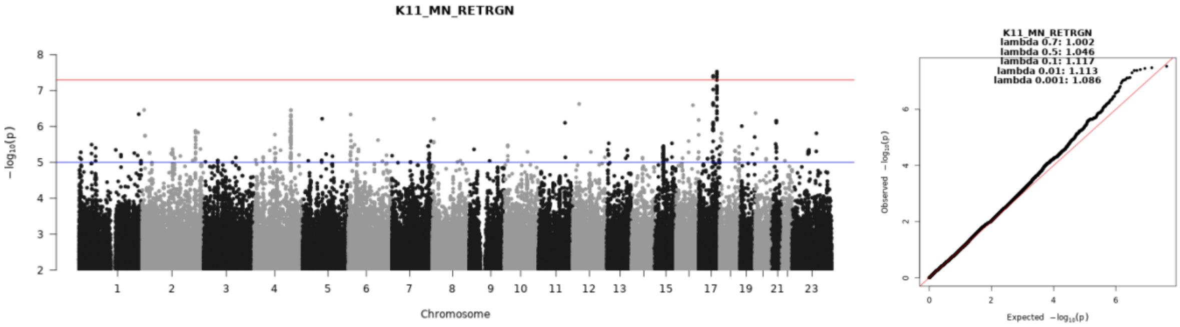

Figure S40. Manhattan and QQ-plot of phenotype Mandibular retrognathia.

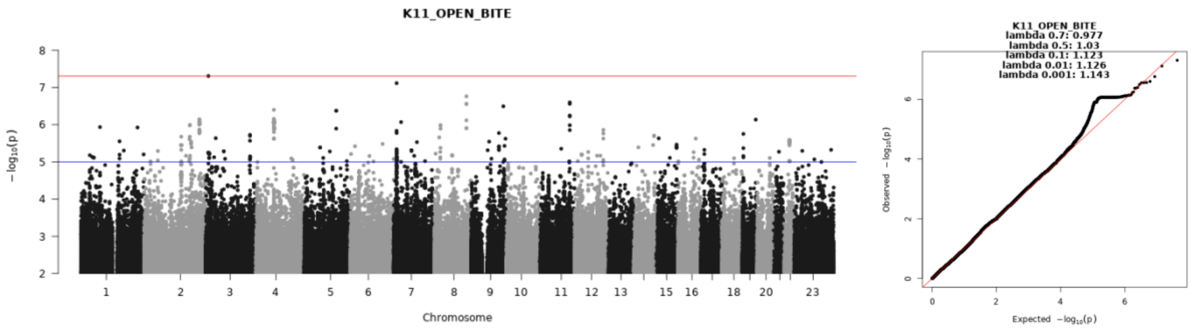

Figure S41. Manhattan and QQ-plot of phenotype Open bite.

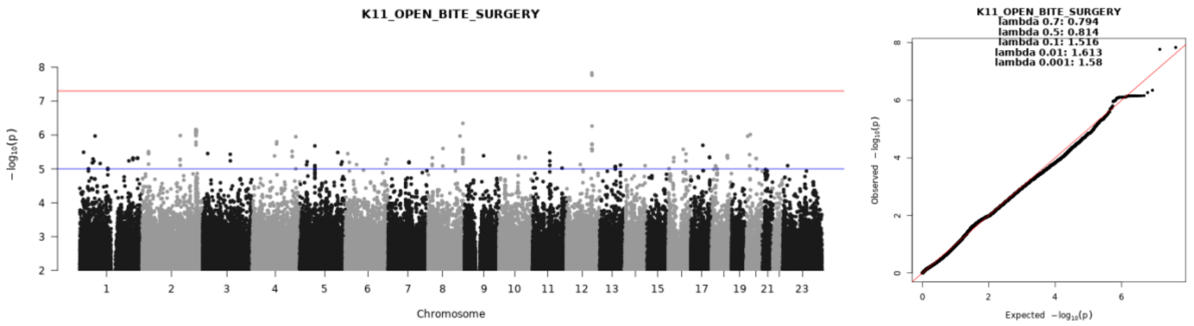

Figure S42. Manhattan and QQ-plot of phenotype Open bite that required surgery (LeFort1 or BSSRO).

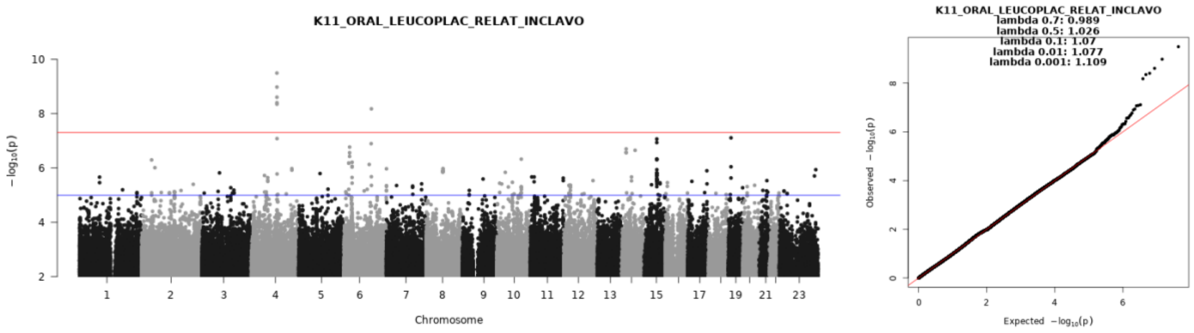

Figure S43. Manhattan and QQ-plot of phenotype Oral leukoplakia and related diseases.

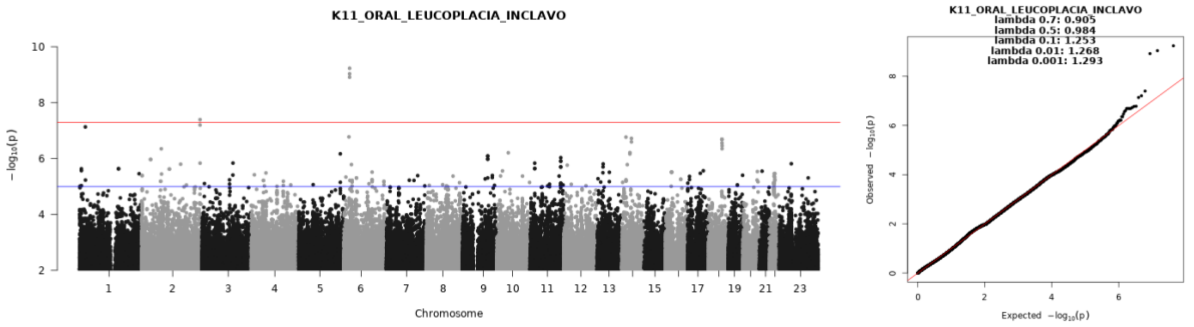

Figure S44. Manhattan and QQ-plot of phenotype Oral leukoplakia.

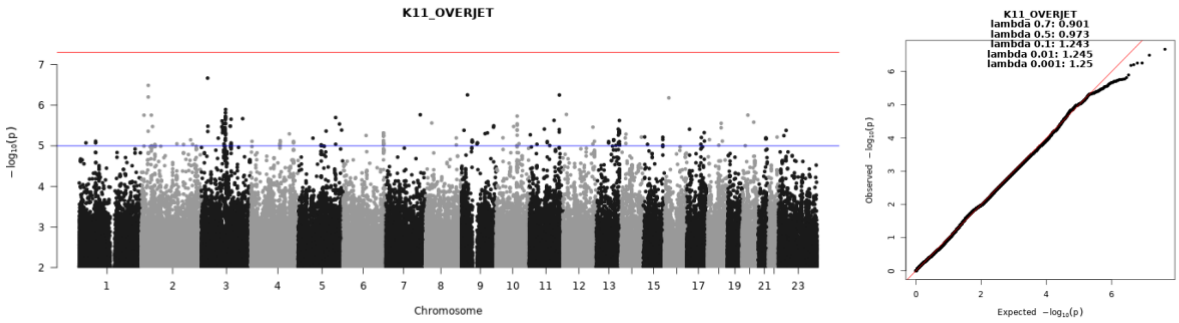

Figure S45. Manhattan and QQ-plot of phenotype Overjet.

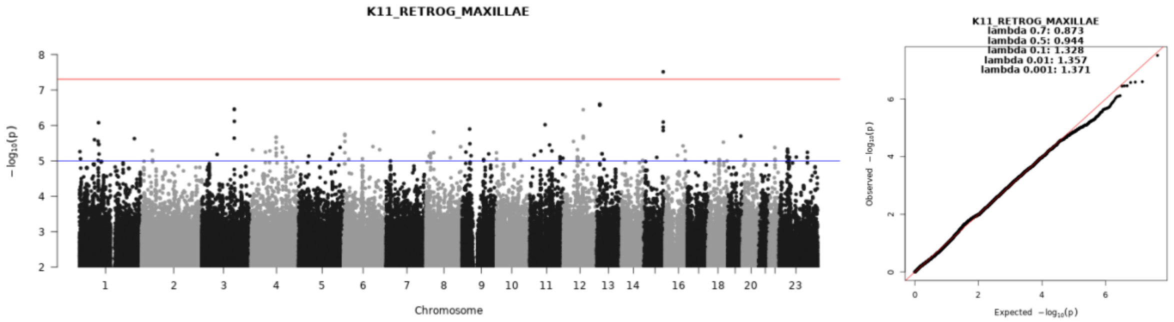

Figure S46. Manhattan and QQ-plot of phenotype Maxillary retrognathism.

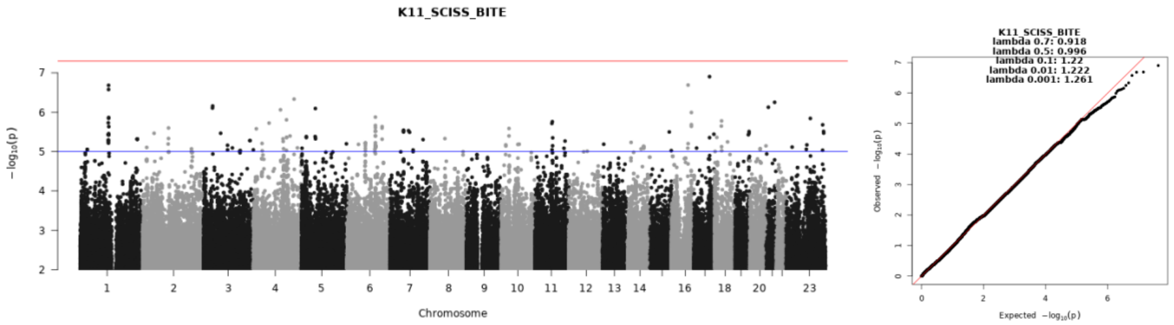

Figure S47. Manhattan and QQ-plot of phenotype Scissor bite.

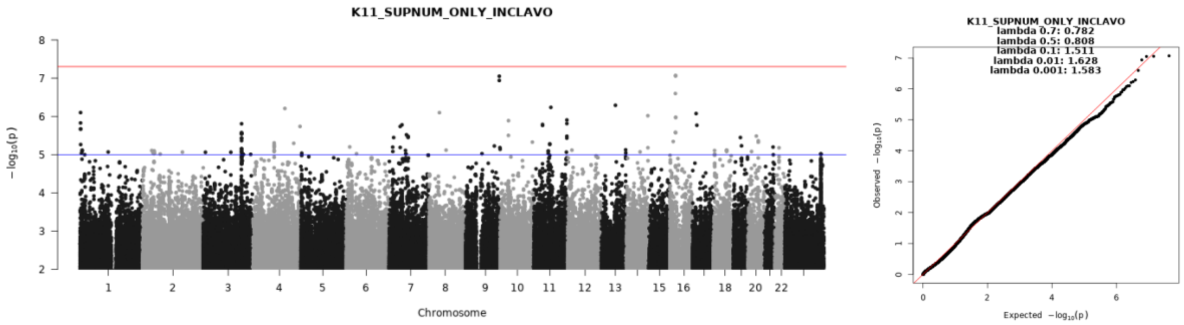

Figure S48. Manhattan and QQ-plot of phenotype Supernumerary teeth (excluding clefts and syndromes).

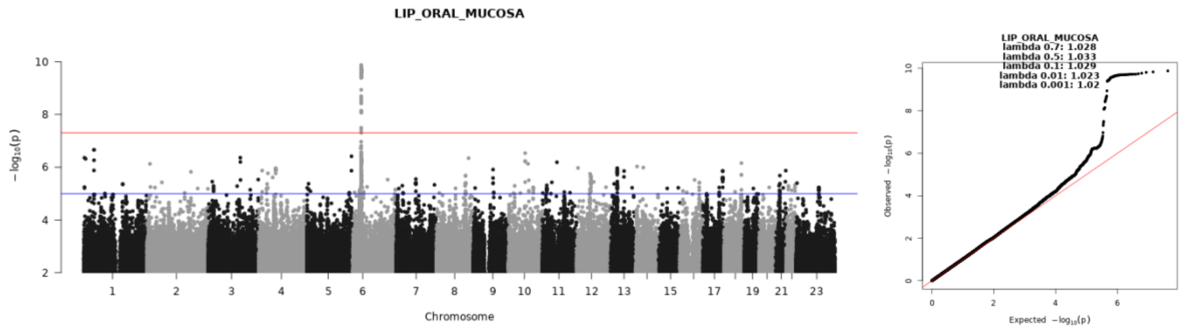

Figure S49. Manhattan and QQ-plot of phenotype Diseases of lip and oral mucosa.

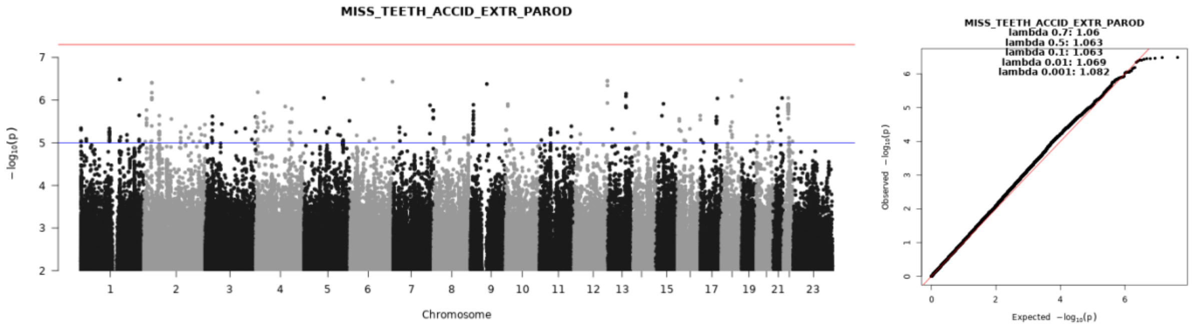

Figure S50. Manhattan and QQ-plot of phenotype Tooth loss.

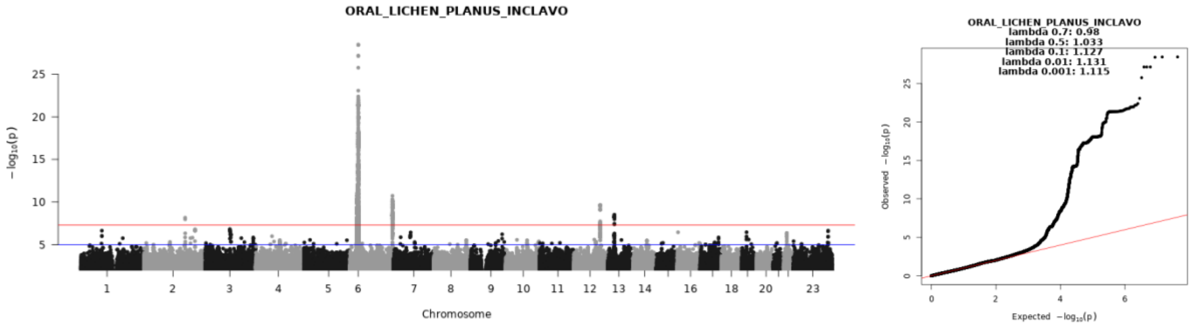

Figure S51. Manhattan and QQ-plot of phenotype Oral lichen planus.

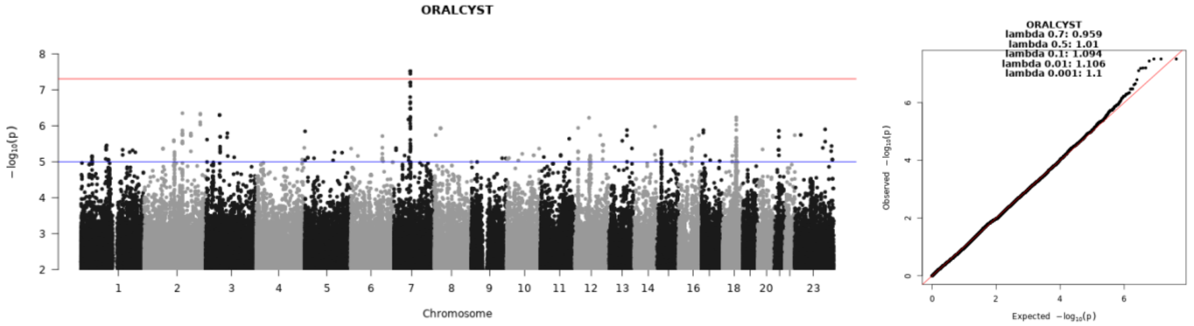

Figure S52. Manhattan and QQ-plot of phenotype Oral cysts.

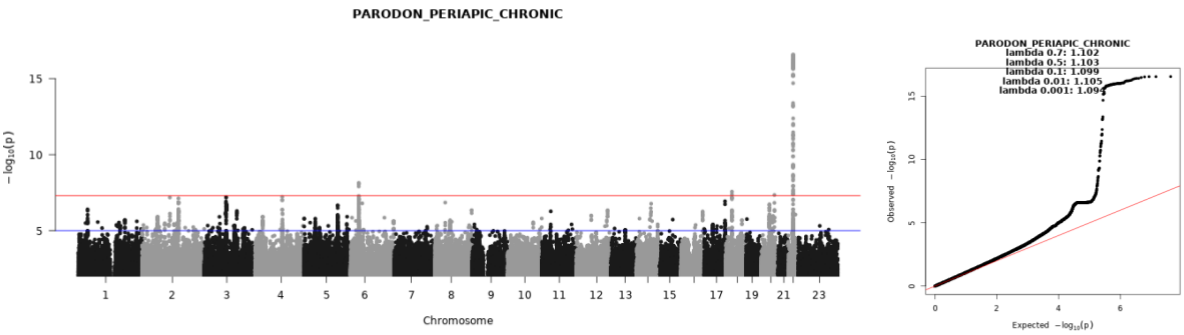

Figure S53. Manhattan and QQ-plot of phenotype Chronic apical periodontitis.

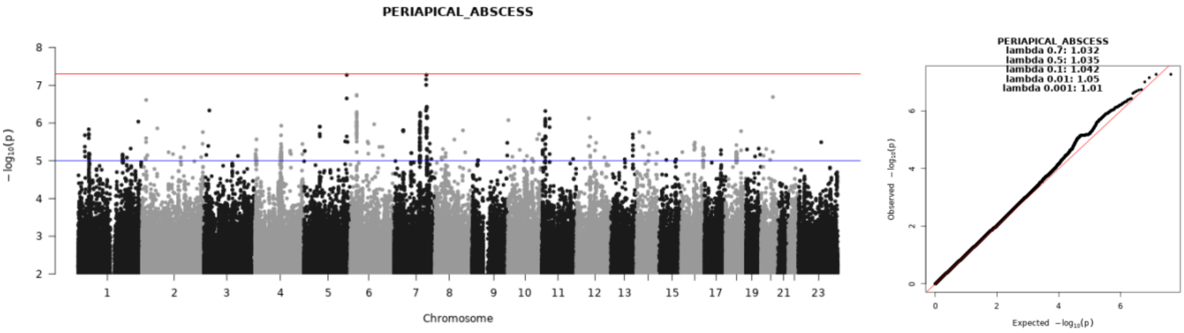

Figure S54. Manhattan and QQ-plot of phenotype Periapical abscess.

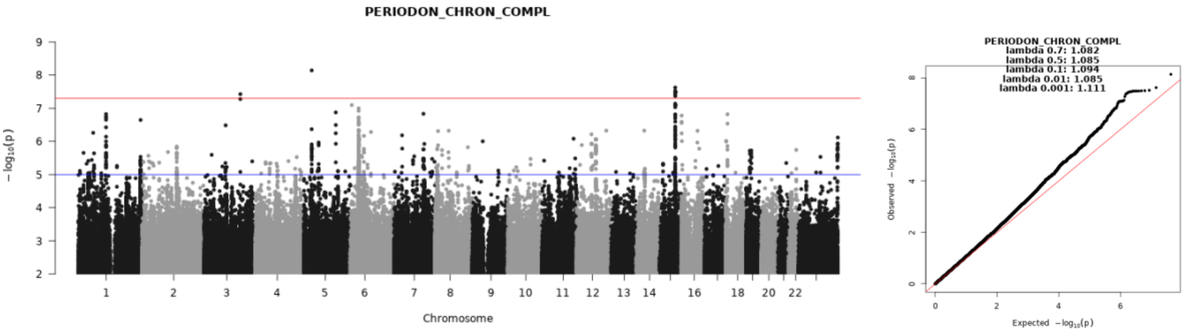

Figure S55. Manhattan and QQ-plot of phenotype Chronic complicated periodontitis.

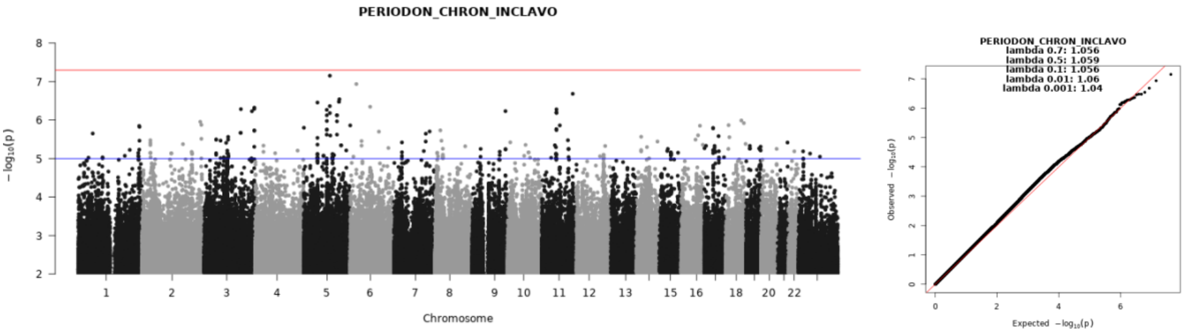

Figure S56. Manhattan and QQ-plot of phenotype Chronic periodontitis.

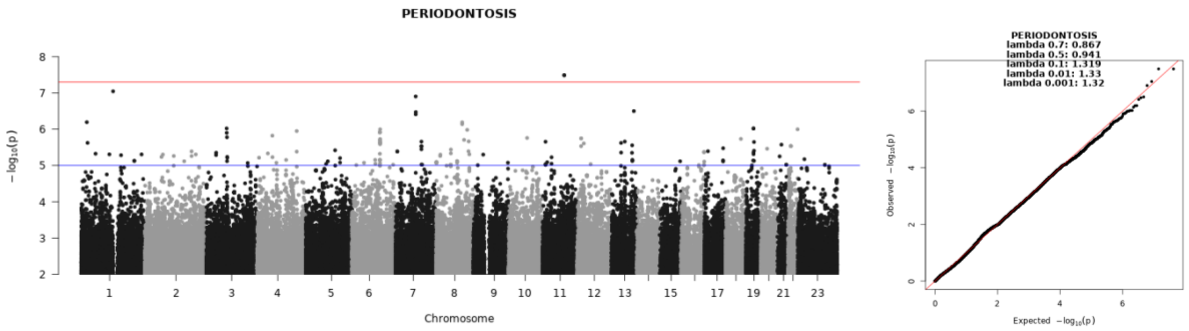

Figure S57. Manhattan and QQ-plot of phenotype Aggressive periodontitis.

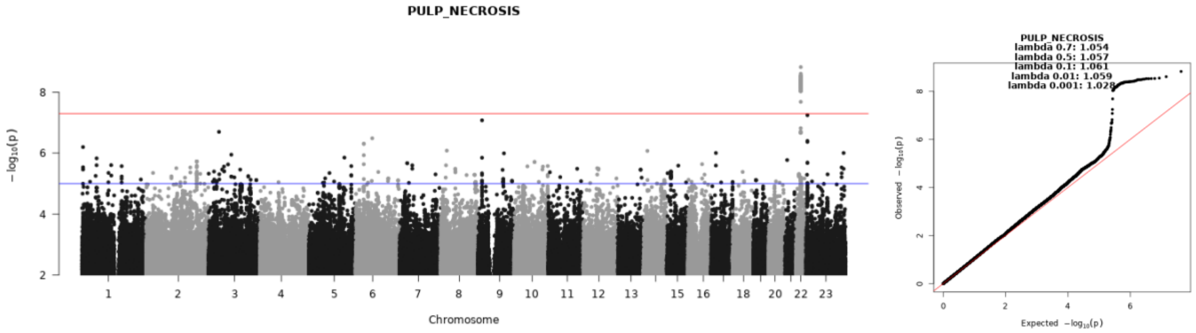

Figure S58. Manhattan and QQ-plot of phenotype Necrosis of pulp.

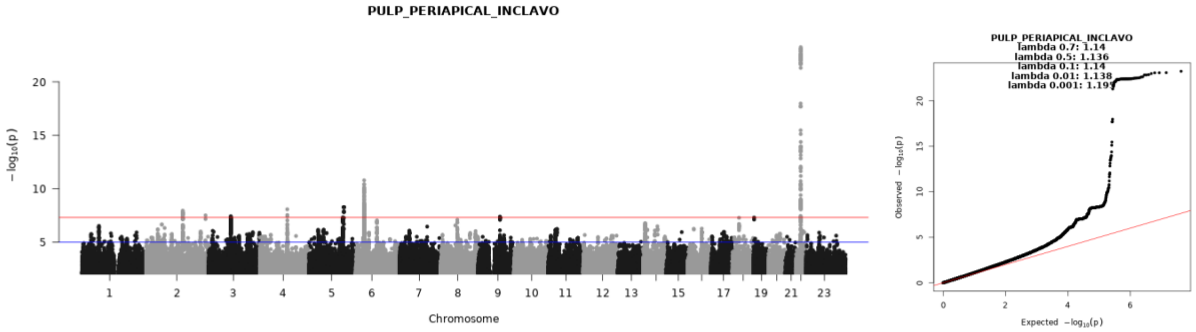

Figure S59. Manhattan and QQ-plot of phenotype Diseases of pulp and periapical tissues.

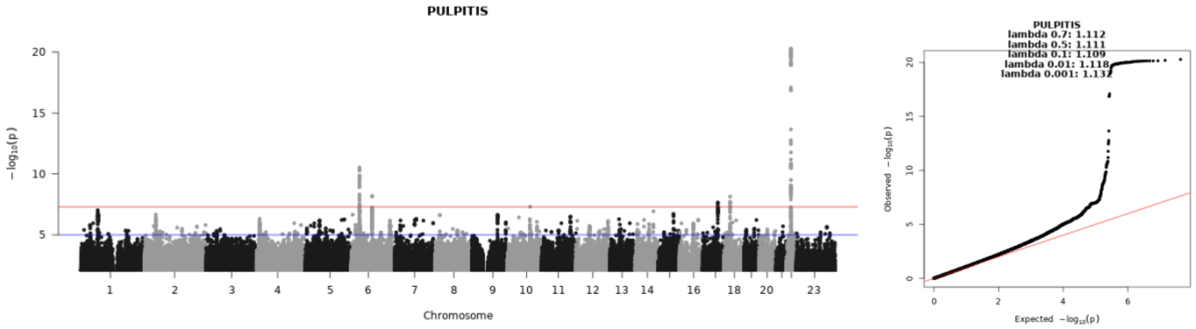

Figure S60. Manhattan and QQ-plot of phenotype Dental pulpitis.

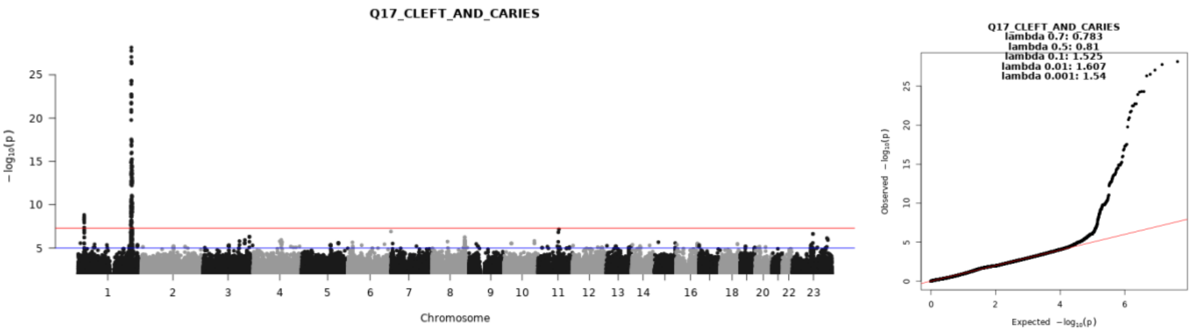

Figure S61. Manhattan and QQ-plot of phenotype Caries in clefts patients.

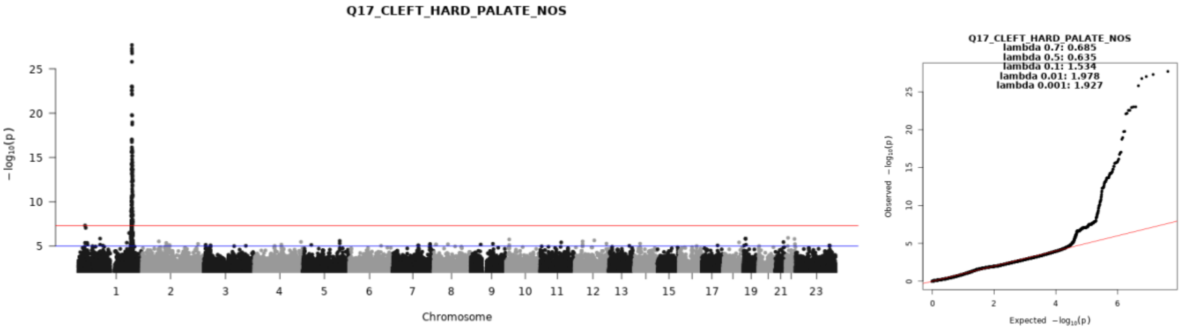

Figure S62. Manhattan and QQ-plot of phenotype Cleft hard palate.

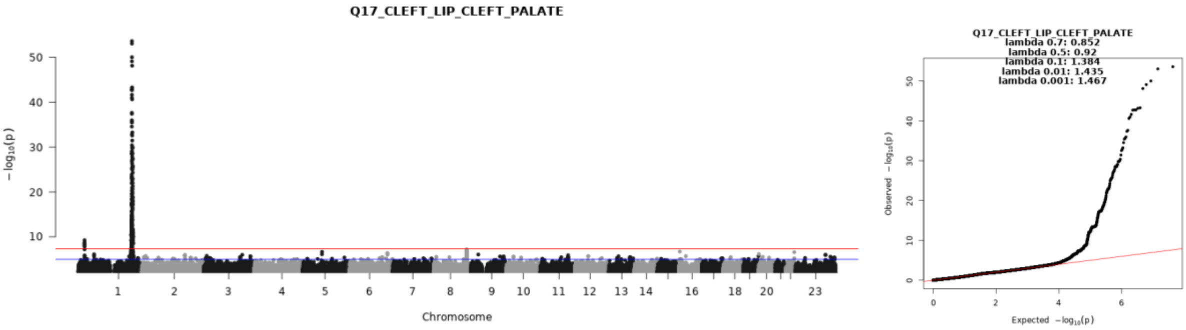

Figure S63. Manhattan and QQ-plot of phenotype Cleft lip, lip and palate or palate.

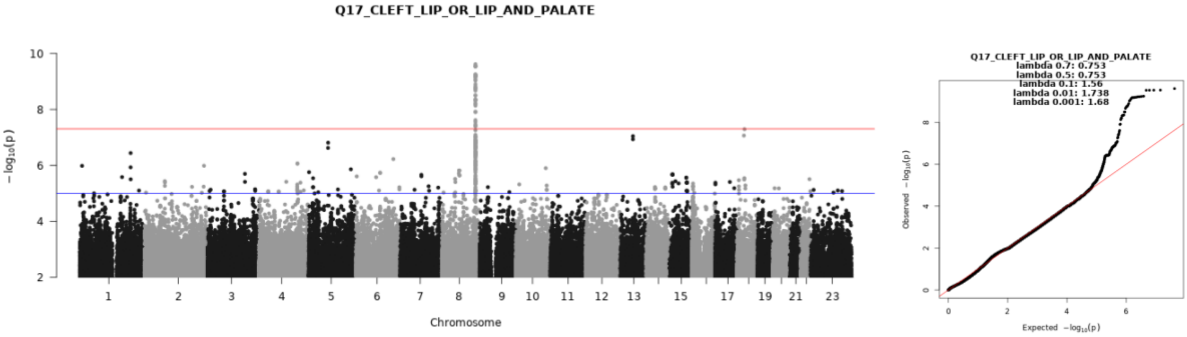

Figure S64. Manhattan and QQ-plot of phenotype Cleft lip or cleft lip and palate.

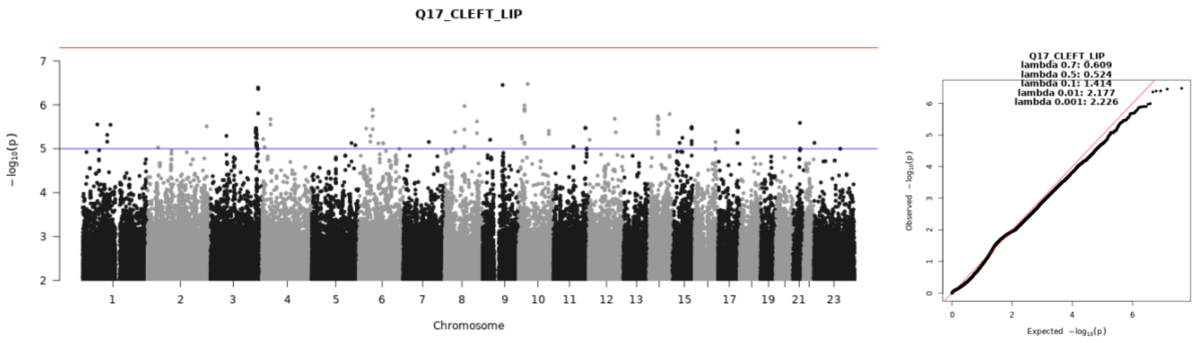

Figure S65. Manhattan and QQ-plot of phenotype Cleft lip.

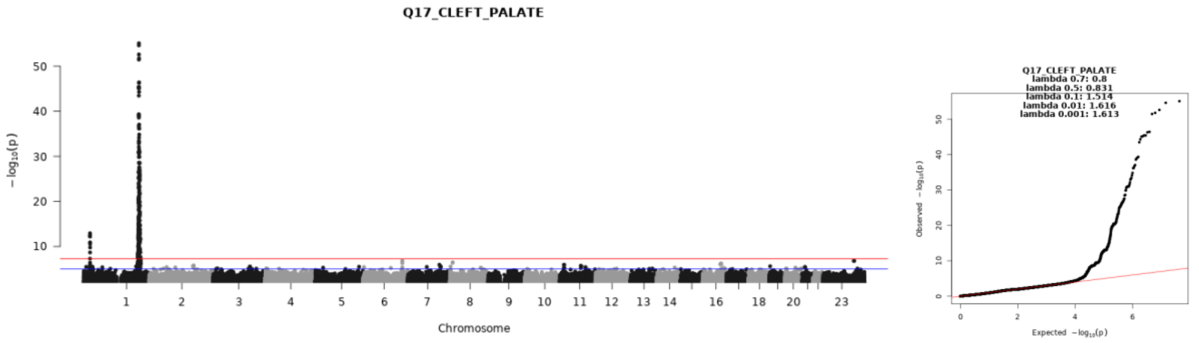

Figure S66. Manhattan and QQ-plot of phenotype Cleft palate.

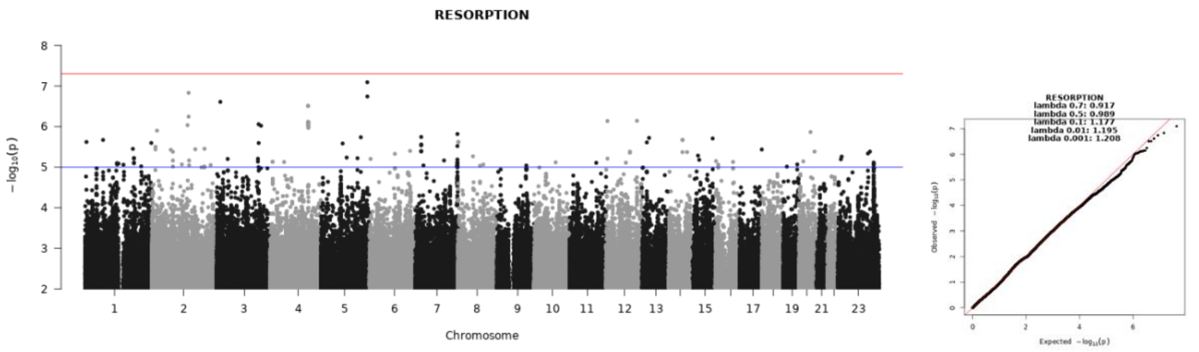

Figure S67. Manhattan and QQ-plot of phenotype Pathological resorption of teeth.

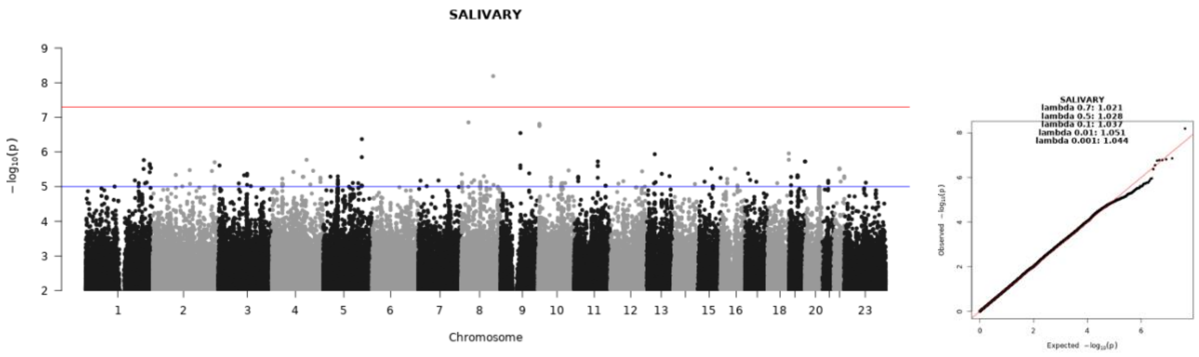

Figure S68. Manhattan and QQ-plot of phenotype Diseases of salivary glands.

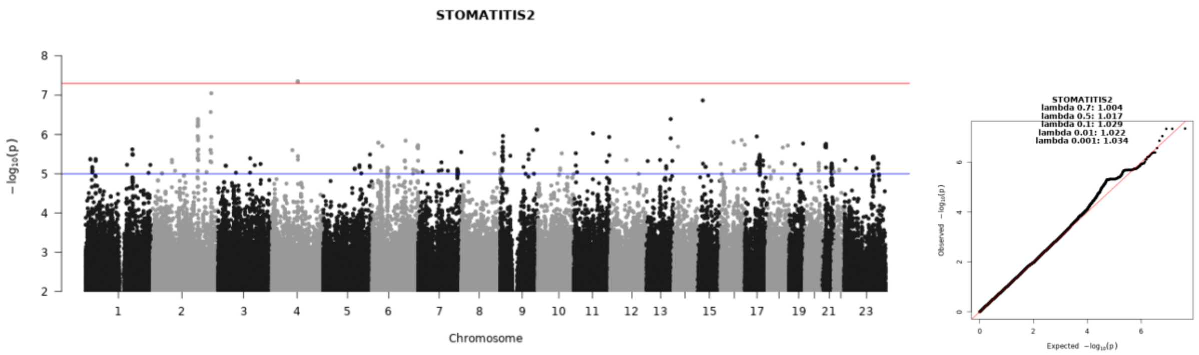

Figure S69. Manhattan and QQ-plot of phenotype Stomatitis and related lesions.

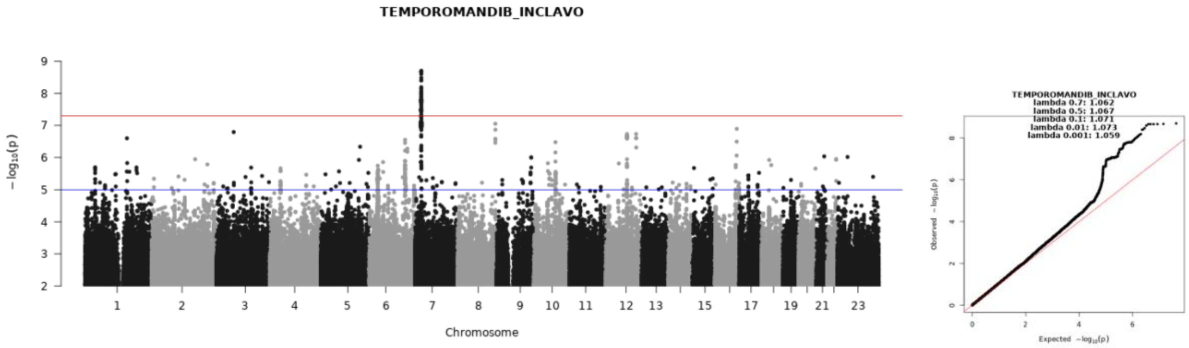

Figure S70. Manhattan and QQ-plot of phenotype Temporomandibular joint disorders.

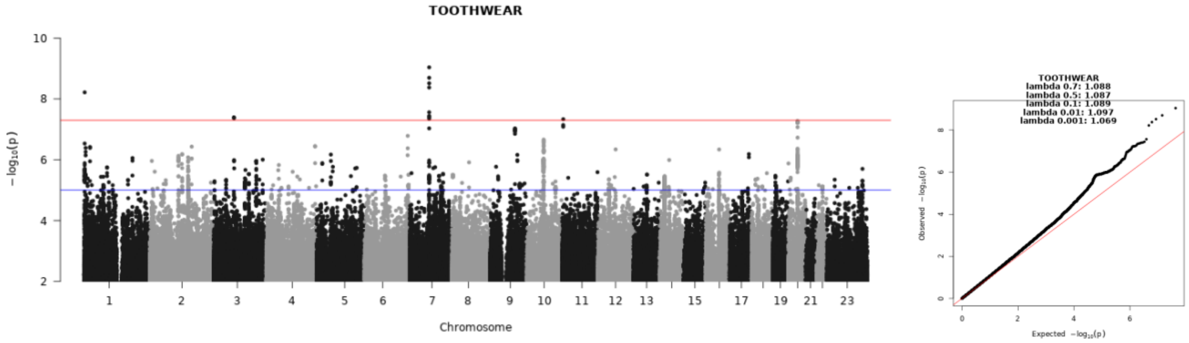

Figure S71. Manhattan and QQ-plot of phenotype Tooth wear.
