# Supplementary material for: Genetic architecture of 67 oral diseases and their links to systemic diseases
Source: HGG Adv. 2026 Jun 17;7(3):100633. doi: 10.1016/j.xhgg.2026.100633 (PMC13343153; doi:10.1016/j.xhgg.2026.100633)
Supplement: Document S2. Article plus supplemental information [file mmc3.pdf]

HGG Advances

ARTICLE

Genetic architecture of 67 oral diseases and their links to systemic diseases

Kirika Karppinen,[1] Hanna M. Ollila,[1,2,3,4] Kanwal Batool,[5] FinnGen, Estonian Biobank Research Team,[5,12] Erik Abner,[5] David P. Rice,[6] Aarno Palotie,[1,2,7] Tuula Palotie,[8] Samuli Ripatti,[1,2,9] Nina Mars,[1,2,*] and Satu Strausz[1,2,3,4,10,11,*]

Summary

Oral and craniofacial diseases are common, yet their genetic basis and links to systemic health are incompletely understood. We performed genome-wide association analyses of 67 oral phenotypes in 500,348 FinnGen participants, identifying 102 genome-wide significant loci, including 45 previously unreported associations. 48 loci remained significant after category-level Bonferroni correction. Fine-mapping revealed 14 coding variants, such as a missense variant in *USP31* for caries and in *MANBA* for oral leukoplakia, and a stop-gained variant in *GPNMB* for temporomandibular disorders. Human leukocyte antigen (HLA) analyses implicated *DQA1* and *DQB1* alleles in lichen planus and other mucosal disorders. We observed 378 statistically significant genetic correlations (r_g) among oral traits, such as tooth loss and chronic apical periodontitis (r_g = 0.91, 95% confidence interval [CI]: [0.76, 1.05], $p = 1.7 \times 10^{-34}$), and 419 significant correlations between oral and systemic diseases, including periodontal diseases with chronic laryngitis ($r_g = 0.97$, 95% CI: [0.58, 1.36], $p = 1.2 \times 10^{-6}$) and bruxism with gastroesophageal reflux ($r_g = 0.51$, 95% CI: [0.38, 0.65], $p = 1.1 \times 10^{-13}$). These results expand the catalog of oral disease loci, uncover Finnish-enriched risk alleles, and highlight shared inflammatory, immune, and structural pathways connecting oral and systemic health.

Introduction

Oral diseases, including dental caries, periodontitis (MIM: 260950), and temporomandibular disorders (TMDs), contribute significantly to the global disease burden, with untreated dental caries affecting 2.3 billion individuals and severe periodontitis affecting over 750 million individuals globally.[1] Despite epidemiologically well-established connections to systemic conditions, including cardiovascular disease, diabetes, and autoimmune disorders,[2–4] the underlying biological and genetic factors remain incompletely characterized. Previous genome-wide association studies (GWASs) have reported SNP-based heritability of 13% for dental caries and 1%–6% for periodontitis, depending on the phenotype definition.[5] Large-scale cohorts with genetic information and diagnostic codes provide an opportunity to explore the shared genetic architecture across different oral diseases and their overlap with systemic diseases.

Oral diseases are complex, multifactorial conditions influenced by genetic, environmental, inflammatory, and microbiome-related factors. While GWASs have primarily focused on common conditions such as caries and periodontitis,[5–8] recent efforts have begun to investigate a broader range of oral phenotypes, for example, pulp and periapical diseases, bruxism (MIM: 606840), and dentofacial developmental anomalies.[9–14] These studies have discovered risk loci involved in immune regulation and barrier defense (e.g., *SIGLEC5* [MIM: 604200] and *DEFA1A3* [MIM: 125220]), craniofacial development (e.g., *IRF6* [MIM: 607199], *NOG* [MIM: 602991], and *SOX9* [MIM: 608160]), and human leukocyte antigen (HLA)-mediated immune pathways, highlighting links between oral traits and systemic health.[6,11–14] However, most GWASs to date remain limited in scale or scope, often focusing on single diseases. This leaves the broader genetic landscape of oral health and overlap with systemic diseases largely uncharacterized.

We therefore aimed to conduct a comprehensive genome-wide investigation of 67 common and rare oral and craniofacial phenotypes within the FinnGen study,[15] leveraging germline genetic data linked to nationwide health registries covering both systemic and dental diagnoses in 500,348 individuals. Here, we explore the shared

[1]Institute for Molecular Medicine Finland, Helsinki Institute of Life Science, University of Helsinki, Helsinki, Finland; [2]Broad Institute of MIT and Harvard, Cambridge, MA, USA; [3]Center for Genomic Medicine, Massachusetts General Hospital, Boston, MA, USA; [4]Anesthesia, Critical Care, and Pain Medicine, Massachusetts General Hospital and Harvard Medical School, Boston, MA, USA; [5]Estonian Genome Centre, Institute of Genomics, University of Tartu, Tartu, Estonia; [6]Orthodontics, Department of Oral and Maxillofacial Diseases, University of Helsinki and Helsinki University Hospital, Helsinki, Finland; [7]Analytic and Translational Genetics Unit (ATGU), Department of Medicine, Department of Neurology and Department of Psychiatry, Massachusetts General Hospital, Boston, MA, USA; [8]Orthodontics, Department of Oral and Maxillofacial Diseases, Clinicum, Faculty of Medicine, University of Helsinki, Helsinki, Finland; [9]Department of Public Health, University of Helsinki, Helsinki, Finland; [10]Department of Oral and Maxillofacial Diseases, Head and Neck Center, Cleft Palate and Craniofacial Centre, Department of Plastic Surgery, University of Helsinki and Helsinki University Hospital, Helsinki, Finland
[11]Lead contact
[12]Further details can be found in the supplemental information
*Correspondence: nina.mars@helsinki.fi (N.M.), satu.strausz@helsinki.fi (S.S.)
https://doi.org/10.1016/j.xhgg.2026.100633.
© 2026 The Author(s). Published by Elsevier Inc. on behalf of American Society of Human Genetics.
This is an open access article under the CC BY license (http://creativecommons.org/licenses/by/4.0/).

and unique genetic architecture of oral and craniofacial phenotypes and their links to systemic diseases. Our study identifies loci associated with these phenotypes and provides insights into genetic risk factors linking oral diseases and general health.

Material and methods

Study cohort

FinnGen is a nationwide research project that integrates genetic and health registry data from over 500,000 individuals in Finland. It combines extensive genetic data with comprehensive longitudinal health data, enabling large-scale GWASs on a wide range of diseases, including oral and systemic conditions.[15]

We utilized data from FinnGen release R12, which includes genetic and health registry data from over 500,000 individuals ($N =$ 500,348). Oral disease phenotypes were identified based on ICD-8, ICD-9, and ICD-10 codes, covering 67 conditions, including diseases of the dental hard tissues, periodontium, oral mucosa, salivary glands, jaw structure, occlusion, and other related oral and craniofacial disorders. Detailed phenotype definitions and case-control numbers are provided in Table S1.

The number of individuals per phenotype category was calculated by counting unique individuals with at least one phenotype in that category. Individuals with multiple phenotypes within the same category were counted only once. Similarly, the number of fine-mapped GWAS loci per category was calculated by counting unique loci associated with at least one phenotype in that category.

Genotyping and sample quality control and imputation

Genotyping in the FinnGen cohort was performed by using Illumina (Illumina, San Diego, CA, USA) and Affymetrix arrays (Thermo Fisher Scientific, Santa Clara, CA, USA) and lifted over to the Genome Reference Consortium Human Build v.38 (GRCh38/hg38). Individuals with high genotype absence (>5%), inexplicit sex, or excess heterozygosity (± 4 standard deviations) were excluded from the data.[15] Additionally, variants that had high absence (>2%), low minor-allele count (<3), or low Hardy-Weinberg equilibrium (HWE) ($p < 1 \times 10^{-6}$) were removed. All individuals in the cohort were Finns and matched against the SISu v.4 reference panel (http://www.sisuproject.fi/).

Before imputation, array-genotyped samples were pre-phased with Eagle 2.3.5 using the default parameters, except for the number of conditioning haplotypes, which was set to 20,000.

Genotype imputation was carried out using the population-specific SISu v.4.2 imputation reference panel with Beagle 4.1 (v.27Jan18.7e1). Post-imputation quality control involved checking the expected conformity of the imputation INFO-value distribution, minor-allele frequency (MAF) differences between the target dataset and the imputation reference panel, and the chromosomal continuity of the imputed genotype calls.

Genome-wide association analysis and fine-mapping

Genome-wide association testing was conducted using the Regenie v.2.2.4 software and the FinnGen Regenie pipeline (https://github.com/FINNGEN/regenie-pipelines/). The analysis was adjusted for current age or age at death, sex, genotyping chip, genetic relationship, and the first 10 principal components (PCs).[16] Further analyses were conducted in R (v.4.5.3).[17] Genome-wide significant associations were defined using the conventional threshold of $p < 5 \times 10^{-8}$. To account for the breadth of the oral phenotype screen, we additionally applied a category-level Bonferroni correction based on the seven phenotype categories shown in Figure 1 by multiplying each GWAS p value by seven. Variants with $p_{adj} < 5 \times 10^{-8}$, corresponding to an unadjusted $p < 7.14 \times 10^{-9}$, were considered significant after category-level Bonferroni correction. Findings meeting this more stringent criterion are indicated in Tables 1, 2, S2, and S3.

To identify genetic variants with the highest likelihood of being causal, a fine-mapping approach was employed using the SuSiE (sum of single effects) model,[18] with the FinnGen fine-mapping pipeline (https://github.com/FINNGEN/finemapping-pipeline). The variants were annotated with the nearest protein-coding gene based on the Ensembl database (release 115).[19] The genes and diseases were annotated with MIM numbers from the OMIM database, where applicable.[20] The Finnish enrichment values (FIN enrichment) were defined as Finnish allele frequency (AF) divided by non-Finnish, non-Swedish, non-Estonian European (NFSEE) AF in gnomAD v.2.1.1 lifted from GRCh37 to GRCh38.[21] Variants not found in gnomAD v.2.1.1 are marked as N/A. The values marked as Inf have an NFSEE AF of 0 in the database.

HLA fine-mapping

HLA fine-mapping was conducted using multivariate logistic regression models with covariates age, sex, cohort, and the first 10 genetic PCs. Imputed HLA allele dosages were obtained from the FinnGen reference panel, and rare alleles with MAFs < 1% were excluded from the analysis.[22]

Cross-trait genetic correlation analysis

We performed cross-trait genetic correlation analyses using linkage disequilibrium score regression (LDSC) with the FinnGen LDSC pipeline (https://github.com/FINNGEN/LDSC). The systemic diseases and conditions used in the analysis were selected based on high population prevalence, including conditions and risk factors with known associations with oral and craniofacial phenotypes. Summary statistics were derived from FinnGen GWAS results (release R12) for systemic disease phenotypes. In addition to the statistical significance level of $p < 0.05$, a more stringent Bonferroni-corrected threshold of $p < 7.14 \times 10^{-3}$ was applied based on the seven phenotype categories. LDSC genetic correlation estimates are not constrained to $[-1, 1]$, and values outside this range may occur due to sampling variability in the heritability and covariance estimates (Tables S6 and S7). SNP-based heritability estimates are presented on the observed scale, based on the European LD reference panel[23] (Table S8). Heatmaps were created using the pheatmap package in R with complete-linkage hierarchical clustering.[24]

Definition of previously unreported loci and variants using GWAS Catalog cross-referencing

Previously unreported GWAS loci were defined according to fine-mapping loci regions. NHGRI-EBI GWAS Catalog variant associations were downloaded (v.1.0, 2025-09-15), and all variants located within the loci regions were searched and filtered for previous genome-wide significant associations with oral or craniofacial phenotypes in the same phenotype category. Diseases of dental hard tissue and pulp and periapical tissue were combined

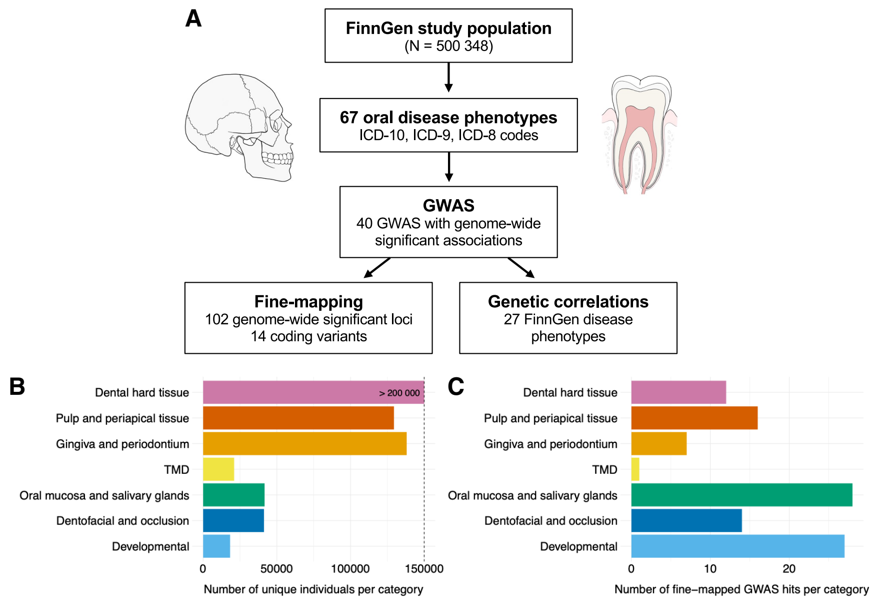

Figure 1. Study overview
(A) Flowchart of study design and data.
(B) Number of individuals per phenotype category. 67 oral and craniofacial phenotypes are grouped into seven phenotype categories. In this visualization, individuals are included only once in each category. The x axis has been limited to 200,000 for visualization purposes.
(C) Number of fine-mapped GWAS loci per phenotype category. Here, loci shared between several phenotypes are included only once in each category.

due to etiological similarities. Lead variants and 95% credible set coding variants were searched from GWAS Catalog to determine any previous GWAS associations.[18]

Replication

Replication analyses of all fine-mapped lead variants and additional 95% credible set coding variants of FinnGen oral phenotypes were performed using summary statistics from 12 UK Biobank and 21 phenotypes from the Million Veteran Program (MVP), obtained from previously published GWASs listed in Tables S9 and S11. In addition, three GWASs were conducted in the Estonian Biobank (EstBB) for replication (Table S10). Replication of variants was assessed in corresponding or category-matched phenotypes, with diseases of dental hard tissue and pulp and periapical tissue combined due to etiological similarities. Full replication results are provided in Tables S13–S15 for the UK Biobank, EstBB, and MVP data, respectively. Nominal evidence of replication was defined as $p < 0.05$ in the replication cohort. The replication results are summarized in Table S12, which lists all replicated variants across the three replication cohorts. The β estimates between FinnGen GWASs and replication analyses are compared in Figure S4.

Ethics statement

Study subjects in FinnGen provided informed consent for biobank research in accordance with the Finnish Biobank Act. Alternatively, separate research cohorts, collected prior to the Finnish Biobank Act coming into effect (in September 2013) and the start of FinnGen (August 2017), were collected based on study-specific consents and later transferred to the Finnish biobanks after approval by Fimea (Finnish Medicines Agency), the National Supervisory Authority for Welfare and Health. Recruitment protocols followed the biobank protocols approved by Fimea. The Coordinating Ethics Committee of the Hospital District of Helsinki and Uusimaa (HUS) statement number for the FinnGen study is Nr HUS/990/2017.

The FinnGen study is approved by Finnish Institute for Health and Welfare (permit numbers: THL/2031/6.02.00/2017, THL/1101/5.05.00/2017, THL/341/6.02.00/2018, THL/2222/6.02.00/2018, THL/283/6.02.00/2019, THL/1721/5.05.00/2019, and THL/1524/5.05.00/2020), Digital and population data service agency (permit numbers: VRK43431/2017-3, VRK/6909/2018-3, and VRK/4415/2019-3), the Social Insurance Institution (permit numbers: KELA 58/522/2017, KELA 131/522/2018, KELA 70/522/2019, KELA 98/522/2019, KELA 134/522/2019, KELA 138/522/2019, KELA 2/522/2020, and KELA 16/522/2020), Findata (permit numbers: THL/2364/14.02/2020, THL/4055/14.06.00/2020, THL/3433/14.06.00/2020, THL/4432/14.06/2020, THL/5189/14.06/2020, THL/5894/14.06.00/2020, THL/6619/14.06.00/2020, THL/209/14.06.00/2021, THL/688/14.06.00/2021, THL/1284/14.06.00/2021, THL/1965/14.06.00/2021, THL/5546/14.02.00/2020, THL/2658/14.06.00/2021, THL/4235/14.06.00/2021, and THL/4990/14.02.00/2023), Statistics Finland (permit numbers: TK-53-1041-17, TK/143/07.03.00/2020 [earlier TK-53-90-20], TK/1735/07.03.00/2021, and TK/3112/07.03.00/2021), and Finnish Registry for Kidney Diseases permission/extract from the meeting minutes on July 4, 2019.

The Biobank Access Decisions for FinnGen samples and data utilized in FinnGen Data Freeze 13 include THL Biobank BB2017_55, BB2017_111, BB2018_19, BB_2018_34, BB_2018_67, BB2018_71, BB2019_7, BB2019_8, BB2019_26, BB2020_1, BB2021_65, BB22-0025-A01, BB22-0025-A03, BB23-0222-A01, BB22-0025-A04, BB22-0025-A06, BB22-0025-A08, and THLBB2024_30; Finnish Red Cross Blood Service Biobank 7.12.2017, 13.11.2023, and 001-2023; Helsinki Biobank HUS/359/2017, HUS/248/2020, HUS/430/2021 §28 and §29, HUS/150/2022 §12–§18, §23, §58, and §59, HUS/128/2023 §18, BB22-0025-A01, BB22-0025-A02, BB22-0025-A05, BB22-0025-A07, BB22-0025-A09, BB22-0025-A10, BB22-0025-A03, BB23-0222-A01, BB22-0025-A04, BB22-0025-A06, BB22-0025-A08, Amendment_BB22-0025-A05; decision allowing data processing to continue until August 31, 2027: BB_2021-0140, HUS/150/2022 §12, BB_2021-0139, HUS/150/2022 §13, BB_2021-0161,HUS/150/2022 §14, BB_2021-0164, HUS/150/2022 §15, BB_2021-0169, HUS/150/2022 §16, BB_2021-0170, HUS/150/2022 §17, BB_2021-0179, HUS/150/2022 §18, BB_2022-0262, HUS/150/2022 §58, BB22-0067, and HUS/150/2022 §59; Auria Biobank AB17-5154 and amendment #1 (August 17, 2020), amendments BB_2021-0140, BB_2021-0156 (August 26, 2021 and February 2, 2022), BB_2021-0169, BB_2021-0179, BB_2021-0161, AB20-5926 and amendment #1 (April 23, 2020) and its modifications (September 22, 2021), BB_2022-0262, BB_2022-0256, BB22-0025-A01, BB22-0025-A02, BB22-0025-A03, BB23-0222_A01, BB22-0025-A02, BB22-0025-A05, BB22-0025-A07, BB22-0025-A09, BB22-0025-A10, BB22-0025-A03, BB23-0222-A01, BB22-0025-A04,

Table 1. Lead variants of previously unreported loci associated with oral phenotypes in FinnGen

Phenotype	rsID	chrom:pos:ref:alt	β	SE	p	p_{adj}	AF	FIN	Variant	Gene
Attrition	rs42131	7:73397257:A>T	−0.070	0.012	7.50×10^{-9}	5.25×10^{-8}	0.195	1.084	regulatory region	*FZD9*
Tooth wear	rs188833787	3:76017712:A>T	0.224	0.041	4.03×10^{-8}	2.82×10^{-7}	0.008	7.149	intron	*ROBO2*
	rs42129	7:73408518:C>A	−0.061	0.010	9.15×10^{-10}	6.40×10^{-9}	0.176	1.054	upstream gene	*FZD9*
	rs2093587330	11:3989373:A>G	0.817	0.149	4.63×10^{-8}	3.24×10^{-7}	0.001	N/A	noncoding transcript exon	*STIM1*
Caries	rs200486134	16:23069106:C>G	0.864	0.149	7.35×10^{-9}	5.15×10^{-8}	<0.001	0.473	missense	*USP31*
	rs187591243	17:38913563:G>A	0.141	0.024	7.92×10^{-9}	5.54×10^{-8}	0.007	1.726	intron	*LASP1*
Diseases of pulp and periapical tissues	rs4988521	2:229754017:C>G	−0.757	0.137	3.12×10^{-8}	2.18×10^{-7}	<0.001	0.157	intergenic	*TRIP12*
	rs560704998, rs75187107, rs398106121	3:85546839:C>CA	−0.028	0.005	3.77×10^{-8}	2.64×10^{-7}	0.711	1.164	intron	*CADM2*
	rs5871429, rs397804460	5:131585275:G>GA	0.026	0.005	4.80×10^{-8}	3.36×10^{-7}	0.619	0.865	intron	*RAPGEF6*
Pulpitis	rs1321699072	10:89325978:C>T	−0.629	0.115	4.95×10^{-8}	3.47×10^{-7}	0.002	18.719	intron	*LIPA*
Chronic apical periodontitis	rs34648990	20:52084522:G>A	0.316	0.058	4.51×10^{-8}	3.16×10^{-7}	0.002	0.101	3' UTR	*ZFP64*
Gingivitis and periodontal diseases	rs7613444	3:52504284:G>C	0.030	0.005	2.91×10^{-8}	2.03×10^{-7}	0.236	1.471	intron	*STAB1*
	rs111231814	12:24820195:C>A	−0.630	0.108	4.94×10^{-9}	3.46×10^{-8}	0.001	0.520	intron	*BCAT1*
	rs187684552	14:71584969:A>G	0.244	0.043	1.19×10^{-8}	8.31×10^{-8}	0.003	0.268	intron	*SIPA1L1*
Chronic complicated periodontitis	rs75351459	3:141093823:C>T	0.109	0.020	$3.76 \times 10^{-}8$	2.63×10^{-7}	0.059	1.997	intron	*SPSB4*
	rs2877160	5:30781297:C>T	0.058	0.010	7.23×10^{-9}	5.06×10^{-8}	0.620	0.895	intergenic	*CDH6*
	rs1051730	15:78601997:G>A	0.056	0.010	2.38×10^{-8}	1.67×10^{-7}	0.331	0.914	synonymous	*CHRNA3*
Embedded and impacted teeth	rs1182052384	3:138799892:T>C	0.582	0.102	1.15×10^{-8}	8.05×10^{-8}	0.003	Inf	intron	*PIK3CB*
Impacted maxillary canine	rs189229384	4:135451123:A>T	1.344	0.243	3.09×10^{-8}	2.16×10^{-7}	0.011	1.709	intergenic	*PABPC4L*
Enamel hypoplasia	rs117645102	18:52010029:G>A	2.469	0.443	2.48×10^{-8}	1.74×10^{-7}	0.001	7.794	intergenic	*DCC*
Hypodontia	rs531152356	6:5323510:T>G	2.395	0.439	4.91×10^{-8}	3.44×10^{-7}	<0.001	0.000	intron	*FARS2*
	rs2320968	13:22218835:A>G	0.285	0.049	5.93×10^{-9}	4.15×10^{-8}	0.146	1.072	intron	*FGF9*
Cleft hard palate	rs187395934	1:26589269:G>A	3.462	0.635	4.86×10^{-8}	3.40×10^{-7}	0.001	N/A	downstream gene	*RPS6KA1*
Anomalies in dental arch relations	rs149999594	10:35697922:C>T	−0.406	0.070	7.61×10^{-9}	5.33×10^{-8}	0.013	1.230	noncoding transcript exon	*FZD8*
	rs1443684783	18:77083370:TC>T	1.102	0.191	8.02×10^{-9}	5.62×10^{-8}	0.001	Inf	intron	*MBP*
Asymmetry of jaw	rs1412908547	17:6086430:G>C	3.738	0.652	1.01×10^{-8}	7.07×10^{-8}	<0.001	N/A	intron	*WSCD1*
Crowding of teeth	rs201475998	2:108875328: AAAAAAAAAG>A	0.260	0.046	1.25×10^{-8}	8.73×10^{-8}	0.055	8.590	intron	*CCDC138*
	rs929805940	3:2153482:T>C	1.879	0.339	3.00×10^{-8}	2.10×10^{-7}	0.001	N/A	intron	*CNTN4*
Deep bite	rs4938326	11:117034748:G>C	0.172	0.031	3.53×10^{-8}	2.47×10^{-7}	0.114	1.268	intron	*SIK3*
	rs1257741	12:28381636:G>C	−0.144	0.025	1.11×10^{-8}	7.78×10^{-8}	0.227	0.906	intron	*CCDC91*
Maxillary hypoplasia	rs573825482	17:50249671:G>A	4.720	0.821	8.89×10^{-9}	6.23×10^{-8}	<0.001	8.049	intergenic	*TMEM92*
Mandibular prognathia	rs535975844	13:96784159:T>G	2.543	0.455	2.30×10^{-8}	1.61×10^{-7}	0.001	Inf	intron	*HS6ST3*

(*Continued on next page*)

Table 1. *Continued*

Phenotype	rsID	chrom:pos:ref:alt	β	SE	p	p_{adj}	AF	FIN	Variant	Gene
Open bite	rs139374150	3:10913763:G>A	0.685	0.126	4.93×10^{-8}	3.45×10^{-7}	0.011	2.105	intron	*SLC6A11*
Open bite and surgery	rs572104116	12:101645832:G>A	3.019	0.533	1.47×10^{-8}	1.03×10^{-7}	0.001	4.689	intron	*MYBPC1*
Maxillary retrognathia	rs11073419	15:95119863:T>C	−0.396	0.072	3.09×10^{-8}	2.16×10^{-7}	0.206	0.882	intron	*MCTP2*
Temporomandibular disorders	rs191297708	7:23217214:T>C	−0.125	0.021	1.99×10^{-9}	1.39×10^{-8}	0.067	18.635	intergenic	*NUP42*
Oral cysts	rs181890726	7:63794711:A>G	−0.539	0.097	3.05×10^{-8}	2.13×10^{-7}	0.035	0.677	downstream gene	*ZNF722*
Stomatitis	rs201138667	4:96283898:TA>T	0.424	0.077	4.46×10^{-8}	3.12×10^{-7}	0.009	N/A	intergenic	*PDHA2*
Oral leukoplakia and other epithelial disorders	rs74649783	4:102502645:A>G	0.444	0.071	3.19×10^{-10}	2.23×10^{-9}	0.024	1.848	intron	*NFKB1*
	rs542270346	6:110242809:T>C	0.987	0.170	6.61×10^{-9}	4.63×10^{-8}	0.004	1.366	intron	*CDC40*
Oral leukoplakia	rs755173701	2:234643265:T>C	2.385	0.434	4.03×10^{-8}	2.82×10^{-7}	0.001	3.122	intergenic	*ARL4C*
	rs1172595265	6:22745355:G>A	4.742	0.775	9.17×10^{-10}	6.42×10^{-9}	<0.001	0.000	intron	*HDGFL1*
Peripheral and oral lichen planus	rs189942924	6:23106848:T>C	0.557	0.099	1.82×10^{-8}	1.27×10^{-7}	0.005	56.253	downstream gene	*HDGFL1*
Diseases of salivary glands	rs760512017	8:117300695: C>CAAA	0.614	0.106	6.47×10^{-9}	4.53×10^{-8}	0.003	2.675	regulatory region	*SLC30A8*
Hypertrophy of tongue papillae	rs1178338740	2:206245999: AAAAAG>A	3.205	0.567	1.63×10^{-8}	1.14×10^{-7}	<0.001	N/A	intron	*CMKLR2*

Genome-wide association analysis identified 102 loci including previously unreported loci across 67 oral and craniofacial phenotypes. Variants shown are the most strongly associated in each locus according to fine-mapping, and the loci have no previously reported genome-wide significant associations within the same phenotype category in the GWAS Catalog.[18] p_{adj} was calculated as $p \times 7$ to account for the seven oral phenotype categories; variants with $p_{adj} < 5 \times 10^{-8}$ are considered significant after category-level Bonferroni correction. chrom, chromosome; pos, position; ref, reference allele; alt, alternative allele; SE, standard error; *p*, *p* value; AF, allele frequency in FinnGen for the alternative allele; FIN, Finnish enrichment of variant.

BB22-0025-A06, and BB22-0025-A08; decision allowing data processing to continue until August 31, 2027: AB20-5926, BB_2021-0140, BB_2021-0156, BB_2021-0161, BB_2021-0161, BB_2021-0164, BB_2021-0169, BB_2021-0179, and BB_2022-0262; Biobank Borealis of Northern Finland_2017_1013, 2021_5010, 2021_5010 amendment, 2021_5018, 2021_5018 amendment, 2021_5015, 2021_5015 amendment, 2021_5015 Amendment_2, 2021_5023, 2021_5023 amendment, 2021_5023 Amendment_2, 2021_5017, 2021_5017 amendment, 2022_6001, 2022_6001 amendment, 2022_6006 amendment, 2022_6006 Amendment_2, BB22-0067, 2022_0262, 2022_0262 amendment, BB22-0025-A01, BB22-0025-A02, BB22-0025-A05, BB22-0025-A07, BB22-0025-A09, BB22-0025-A10, BB22-0025-A03, BB23-0222-A01, BB22-0025-A04, BB22-0025-A06, and BB22-0025-A08; decision allowing data processing to continue until August 31, 2027: BB/2021/5015, BB/2021/5017, BB/2021/5018, BB/2021/5023, BB/2022/6006, BB/2022/6001, BB/2022-0262, and BB/2021/5010; Biobank of Eastern Finland 1186/2018 and amendment 22§/2020, 53§/2021, 13§/2022, 14§/2022, 15§/2022, 27§/2022, 28§/2022, 29§/2022, 33§/2022, 35§/2022, 36§/2022, 37§/2022, 39§/2022, 7§/2023, 32§/2023, 33§/2023, 34§/2023, 35§/2023, 36§/2023, 37§/2023, 38§/2023, 39§/2023, 40§/2023, 41§/2023, BB22-0025-A01, BB22-0025-A02, BB22-0025-A05, BB22-0025-A07, BB22-0025-A09, BB22-0025-A10, BB22-0025-A03, BB23-0222-A01, BB22-0025-A04, BB22-0025-A06, and BB22-0025-A08; decision allowing data processing to continue until August 31, 2027: MO-BB_2021-0179-A0, MO-BB_2021-0156_PRE-A01, BB_2021-0140, MO-BB_2021-0170_PRE-A0, MO-BB_2021-0169-A01, MO-BB_2022-0256-A01, MO-BB_2021-0161-A01, MO-BB_2021-0161-A02, BB22-0067-A01, and MO-BB_2022-0262-A0; Finnish Clinical Biobank Tampere MH0004 and amendments (21.02.2020 and 06.10.2020), BB2021-0140 8§/2021, 9§/2021, §9/2022, §10/2022, §12/2022, 13§/2022, §20/2022, §21/2022, §22/2022, §23/2022, 28§/2022, 29§/2022, 30§/2022, 31§/2022, 32§/2022, 38§/2022, 40§/2022, 42§/2022, 1§/2023, BB2021-0140, BB22-0025-A01, BB_2021-0161, BB22-0025-A02, BB22-0025-A05, BB22-0025-A07, BB22-0025-A09, BB22-0025-A10, BB22-0025-A03, BB23-0222-A01, BB22-0025-A04, BB22-0025-A06, and BB22-0025-A08; decision allowing data processing to continue until August 31, 2027: BB_2021-0140, BB_ 2021-0161, BB_ 2021-0179, BB_ 2021-0156, BB_ 2021-0169, BB_ 2021-0170, and BB22-0067-A01; Central Finland Biobank 1-2017, BB_2021-0169, BB_2021-0179, BB_2022-0256, and BB_2022-0262; decision allowing data processing to continue until August 31, 2027 for projects: BB_2021-0179, BB22-0067,BB_2022-0262, BB_2021-0170, BB_2021-0164, BB_2021-0161, BB_2021-0169, BB22-0025-A01, BB22-0025-A02, BB22-0025-A05, BB22-0025-A07, BB22-0025-A09, BB22-0025-A10, BB22-0025-A03, BB23-0222-A01, BB22-0025-A04, BB22-0025-A06, and BB22-0025-A08; Terveystalo Biobank STB 2018001 and amendment August 25, 2020; Finnish Hematological Registry and Clinical Biobank decision June 18, 2021, amendment January 2, 2024; and Arctic Biobank P0844: ARC_2021_1001, ARC_2023_3003 (BB22-0025-A01), BB22-0025-A03, BB23-0222-A01, BB22-0025-A04, BB22-0025-A06, and BB22-0025-A08.

The EstBB is a volunteer-based biobank with 212,955 participants in the current data freeze.[25] All biobank participants have signed a broad informed consent form, and information on ICD-10 codes is obtained via regular linking with the national Health Insurance Fund and other relevant databases, with the majority of the electronic health records having been collected since 2004.[26] Analyses were restricted to individuals with European ancestry. The activities of the EstBB are regulated by the

Table 2. **Lead and credible set coding variants of previously unreported loci associated with oral phenotypes in FinnGen**

Phenotype	rsID	chrom:pos:ref:alt	β	SE	p	p_{adj}	AF	FIN	Variant	Nearest gene
Caries	rs200486134	16:23069106:C>G	0.864	0.149	7.35×10^{-9}	5.15×10^{-8}	<0.001	0.473	missense	*USP31*
Hypodontia	rs4129190	2:88173272:G>A	−0.323	0.051	3.53×10^{-10}	2.47×10^{-9}	0.123	1.012	missense	*THNSL2*
Oral leukoplakia and other epithelial disorders	rs75826658	4:102632215:C>T	0.428	0.070	1.04×10^{-9}	7.28×10^{-9}	0.025	1.775	missense	*MANBA*
Oral lichen planus	rs9315906	13:42303129:G>C	−0.280	0.048	5.69×10^{-9}	3.98×10^{-8}	0.137	0.839	synonymous	*AKAP11*
Temporomandibular disorders	rs11537976	7:23274204:G>T	−0.125	0.021	2.17×10^{-9}	1.52×10^{-8}	0.067	15.926	stop gained	*GPNMB*

Genome-wide association analysis identified 14 coding variants from fine-mapped loci associated with oral and craniofacial phenotypes. Variants shown are coding lead variants and coding variants from 95% credible sets, which have no previously reported genome-wide significant associations in the GWAS Catalog.[18] chrom, chromosome; pos, position; ref, reference allele; alt, alternative allele; se, standard error; p, p-value; AF, allele frequency in FinnGen for the alternative allele; FIN, Finnish enrichment of variant. p_{adj} was calculated as $p \times 7$ to account for the seven oral phenotype categories; variants with $p_{adj} < 5 \times 10^{-8}$ were considered significant after category-level Bonferroni correction.

Human Genes Research Act, which was adopted in 2000 specifically for the operations of the EstBB. Individual-level data analysis in the EstBB was carried out under ethical approval 1.1-12/624 from the Estonian Committee on Bioethics and Human Research (Estonian Ministry of Social Affairs), using data according to release application 6-7/GI/2014 from the EstBB.

Results

Oral phenotypes span a wide clinical spectrum

The FinnGen dataset includes 500,348 individuals with genome-wide genotyping and comprehensive health registry data, including lifetime medical diagnoses, prescription and purchase data, laboratory values, sociodemographic factors, and cause-of-death records. We assessed 67 oral and craniofacial phenotypes spanning a range of structures from dental hard tissue to oral mucosa and salivary glands (Figure 1A; Table S1). All 67 phenotypes were grouped into seven categories based on tissue type and diagnostic similarity, as shown in Figures 1B and 1C.

The most prevalent diseases were dental caries (n = 223,126) and gingivitis and periodontal diseases (n = 137,746), followed by diseases of pulp and periapical tissues (n = 129,318), chronic apical periodontitis (n = 75,784), and pulpitis (n = 50,909). Rarer diagnoses included oral leukoplakia (n = 930), cleft palate (n = 388 [MIM = 119540]), and impacted maxillary canine (n = 311), highlighting the wide phenotypic range of the dataset (Figures 1B and S1; Table S1).

Genome-wide association analysis identifies 102 loci for oral and craniofacial phenotypes

We conducted GWASs for all 67 phenotypes and identified genome-wide significant associations for 40 phenotypes. For these 40 phenotypes, we performed fine-mapping to identify lead and credible set variants, which revealed 102 independent genome-wide significant loci (Table S2). Of these, 48 loci remained significant after category-level Bonferroni correction ($p_{adj} < 5 \times 10^{-8}$) and are highlighted as Bonferroni-supported findings in Tables 1 and S2. The full genome-wide significant discovery set is reported throughout.

The genome-wide significant discovery set included lead variants for high-burden traits such as caries (e.g., rs187591243 in *LASP1* [MIM: 602920], β = 0.14, p = 7.9×10^{-9}) and gingivitis/periodontitis (e.g., rs187684552 in *SIPA1L1* [MIM: 617504], β = 0.24, p = 1.2×10^{-8}), as well as for developmental anomalies such as cleft hard palate (rs187395934 near *RPS6KA1* [MIM: 601684], β = 3.46, p = 4.9×10^{-8}) and impacted maxillary canine (rs189229384 near *PABPC4L* [MIM: 603407], β = 1.34, p = 3.1×10^{-8}). A subset of variants showed notably large effect sizes, particularly those associated with rare traits such as hypertrophy of tongue papillae (rs1178338740 in *CMKLR2* [MIM: 600239], β = 3.21, p = 1.6×10^{-8}) and maxillary hypoplasia (rs573825482 near *TMEM92* [MIM: 619604], β = 4.72, p = 8.9×10^{-9}) (Table 1).

Bonferroni-supported loci included variants for tooth wear (rs42129 near *FZD9* [MIM: 601766]), hypodontia/oligodontia (rs2320968 near *FGF9* [MIM: 600921]), oral leukoplakia and epithelial disorders (rs74649783 in *NFKB1* [MIM: 164011]), TMDs (rs191297708 near *NUP42* [MIM: 619998]), and diseases of salivary glands (rs760512017 near *SLC30A8* [MIM: 611145]), all with $p_{adj} < 5 \times 10^{-8}$ (Table 1).

Coding variants from fine-mapped loci

Among the lead variants, we identified a previously unreported missense variant for caries (rs200486134 in *USP31* [MIM: 619536], β = 0.86, p = 7.4×10^{-9}). When examining the 95% credible set variants of fine-mapped loci, nine additional coding variants were identified, including five previously unreported variants (Tables 2, S3, and S4). Of the coding variants shown in Table S3, seven variants met the category-level Bonferroni threshold ($p_{adj} < 5 \times 10^{-8}$). Additional coding variants included missense variants for oral leukoplakia and other epithelial disorders (rs75826658 in *MANBA* [MIM: 609489], β = 0.43, p = 1.0×10^{-9}) and hypodontia/oligodontia (rs4129190 in *THNSL2* [MIM: 611261], β = −0.32, p = 3.5×10^{-10}), and a stop-gained variant for TMD (rs11537976 in *GPNMB* [MIM: 604368], β = −0.13,

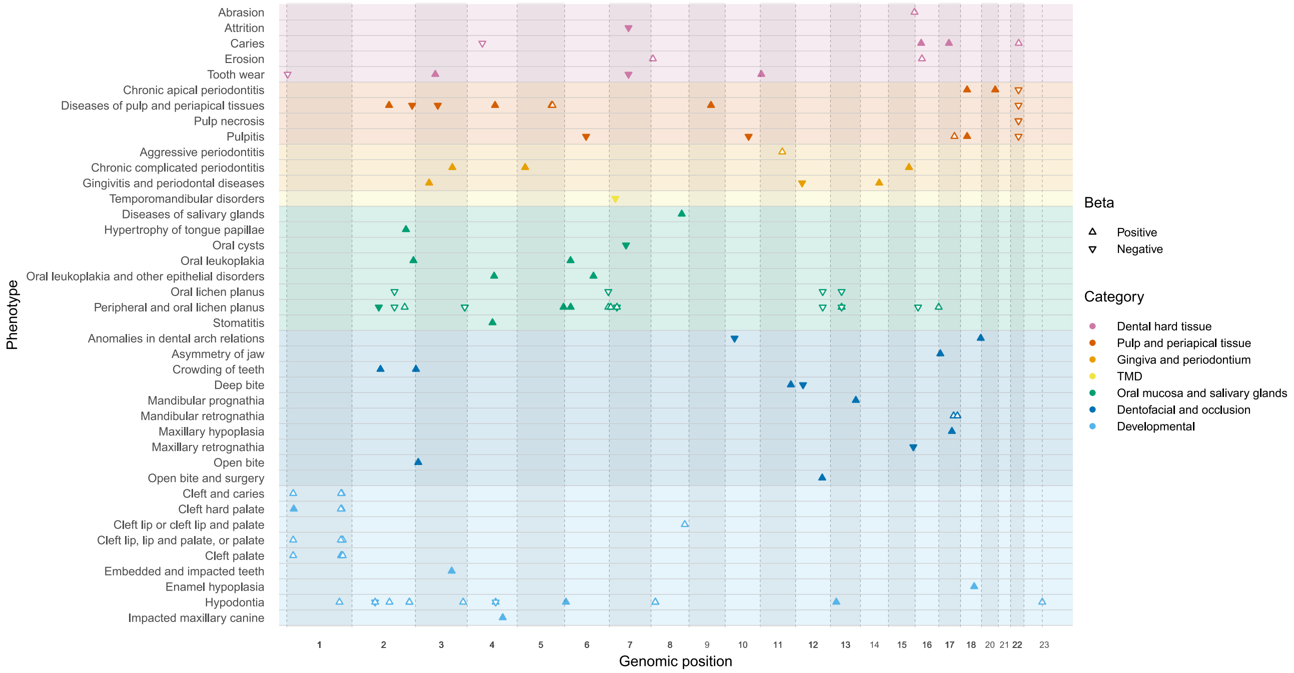

Figure 2. The distribution of fine-mapped GWAS loci across chromosomes for oral and craniofacial phenotypes
The approximate genomic positions of the variants are shown for each phenotype on the x axis. Phenotypes are organized by phenotype category on the y axis. Lead variants of previously unreported loci are highlighted. The direction of the triangle indicates the sign of the lead variant β estimate.

$p = 2.2 \times 10^{-9}$, FIN = 15.93). In addition, several known associations were observed, such as missense variants for hypodontia/oligodontia (MIM: 604625; rs121908120 in *WNT10A* [MIM: 606268], $\beta = 1.07$, $p = 1.3 \times 10^{-11}$) and orofacial cleft phenotypes (rs41268753 in *GRHL3* [MIM: 608317]).

Hypodontia/oligodontia showed convergent evidence across both associated loci and coding variants. In addition to the lead signal near *FGF9*, coding variants in *WNT10A* and *THNSL2* were identified. *WNT10A* is a well-established tooth agenesis gene, and *FGF9* has recognized roles in developmental signaling.[27,28] Exploratory functional annotation of the highlighted genes (*FGF9*, *WNT10A*, and *THNSL2*) suggested enrichment in signaling-related molecular functions, including receptor-ligand activity and signaling receptor activator/regulator activity (GO: 0048018, GO: 0030546, and GO: 0030545).

63 variants highlight biology of underexplored traits

To assess the genomic distribution of oral disease risk, we mapped the locations of lead variants across the chromosomes. The lead variants were distributed across all autosomes, with notable clusters on chromosomes 1, 2, 4, and 7 (Figure 2). The genome-wide significant associations span diverse phenotype categories, emphasizing the polygenic nature of oral disease susceptibility.

Of the 102 fine-mapped loci, 45 had no prior associations in previously published GWAS of similar oral phenotypes based on the GWAS Catalog and literature search. 63 lead variants had no prior associations in previously published GWASs. These loci were particularly enriched in dentofacial traits: among the 45 previously unreported loci, nearly one-third (12/45) were in dentofacial anomalies and malocclusions, for example, mandibular prognathia (MIM: 176700; rs535975844 in *HS6ST3* [MIM: 609401], $\beta = 2.54$, $p = 2.3 \times 10^{-8}$) and crowding of teeth (rs929805940 in *CNTN4* [MIM: 607280], $\beta = 1.88$, $p = 3.0 \times 10^{-8}$) (Table 1).

Several of the lead variants were enriched in the Finnish population (FIN), such as rs189942924 in peripheral and oral lichen planus (MIM: 151620; *HDGFL1* [MIM: 617884], $\beta = 0.56$, $p = 1.8 \times 10^{-8}$, FIN = 56.25), rs1321699072 in pulpitis (*LIPA* [MIM: 613497], $\beta = -0.63$, $p = 5.0 \times 10^{-8}$, FIN = 18.72), and rs191297708 in TMDs (*NUP42*, $\beta = -0.13$, $p = 2.0 \times 10^{-9}$, FIN = 18.64). In addition, a low-frequency variant, rs1443684783, in anomalies in dental arch relations (*MBP* [MIM: 159430], $\beta = 1.10$, $p = 8.0 \times 10^{-9}$, AF = 0.001) was observed only in the Finnish population (Table 1).

HLA alleles are associated with mucosal and pulpal diseases

We next focused on the HLA region to investigate immunogenetic contributions to oral diseases using a Finnish-specific reference panel for HLA alleles.[22]

HLA fine-mapping revealed strong associations across a range of oral phenotypes, including mucosal, pulpal, and periodontal conditions (Figure S3; Table S5). Most associations, with the exception of attrition, mapped to the HLA class II region, which encodes molecules responsible for presenting extracellularly derived peptides to $CD4^+$

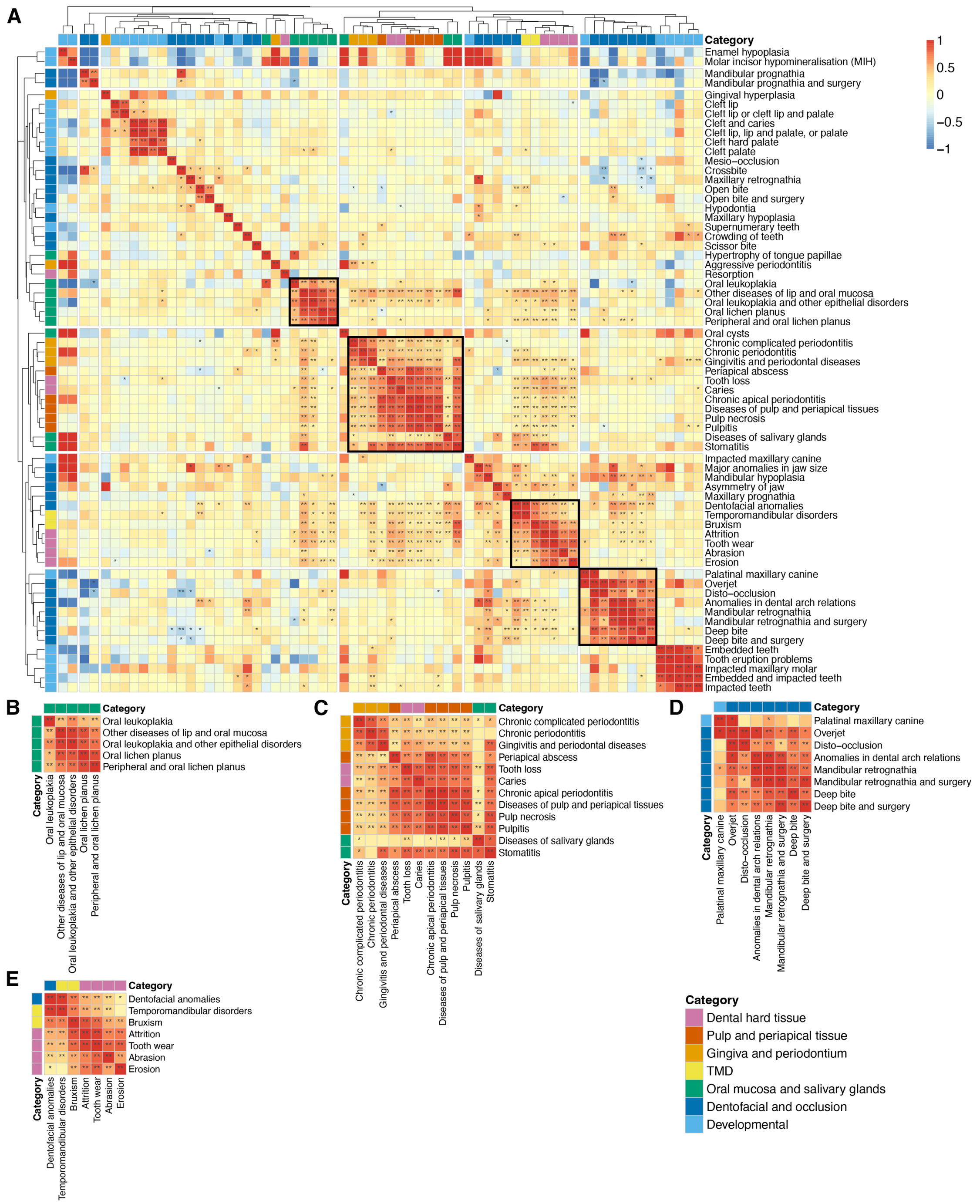

Figure 3. Heatmap of genetic correlations between oral phenotypes

(A) Heatmap of genetic correlations (r_g) between 67 oral and craniofacial phenotypes. Correlation estimates are hierarchically clustered using the complete-linkage method, as shown by dendrograms on both sides of the heatmap. The figure is annotated with phenotype categories. Nominally significant ($7.14 \times 10^{-3} < p < 0.05$) correlation estimates are indicated by a single asterisk (*) and those significant after category-level Bonferroni correction ($p < 7.14 \times 10^{-3}$) by a double asterisk (**).

(B–E) Zoomed-in views of identified clusters.

T cells, thereby initiating and regulating adaptive immune responses.

The strongest associations were observed for oral and peripheral lichen planus (*DQB1*05:01*, $\beta = 0.56$, $p = 7.7 \times 10^{-235}$) and oral lichen planus (*DQA1*01:01*, $\beta = 0.33$, $p = 3.2 \times 10^{-19}$). Additionally, we observed association signals at *DRB4*01:03* in diseases of pulp and periapical tissues ($\beta = 0.03$, $p = 7.4 \times 10^{-6}$), *DQB1*03:01* in chronic apical periodontitis ($\beta = 0.04$, $p = 6.3 \times 10^{-6}$), and *DQA1*01:02* in diseases of the lip and oral mucosa ($\beta = -0.06$, $p = 5.4 \times 10^{-5}$) (Table S5). These results suggest that multiple oral phenotypes share common HLA-mediated inflammatory mechanisms.

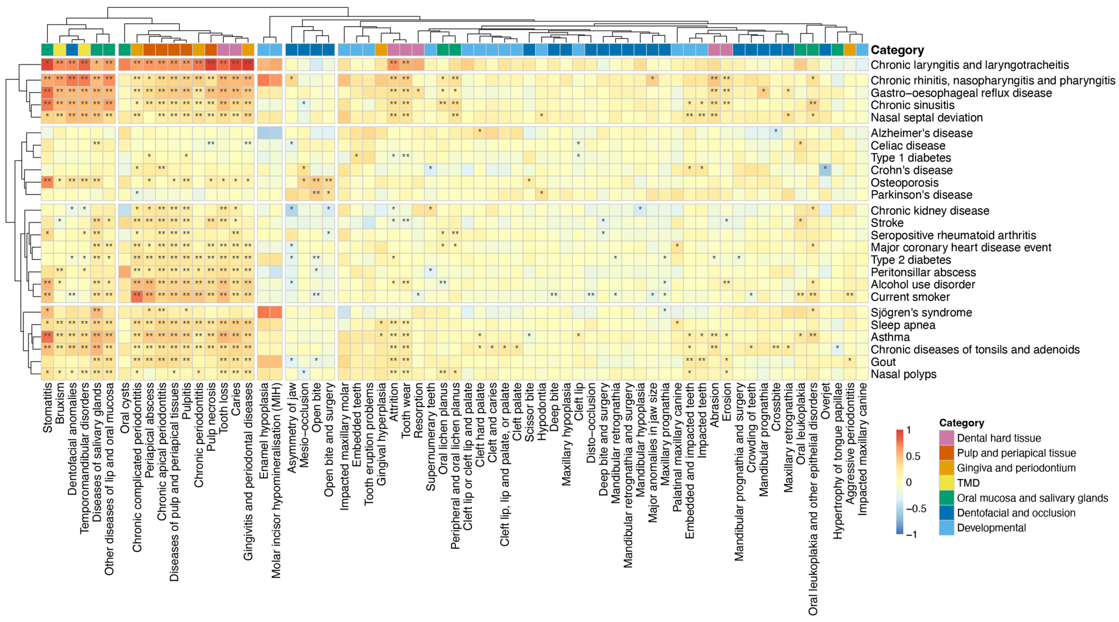

Figure 4. Heatmap of genetic correlations between all oral phenotypes and 27 systemic diseases and phenotypes
Correlation estimates are hierarchically clustered with the complete-linkage method, as shown by dendrograms on both sides of the heatmap. The figure is annotated with oral phenotype categories. Nominally significant ($7.14 \times 10^{-3} < p < 0.05$) correlation estimates are indicated by a single asterisk (*) and those significant after category-level Bonferroni correction ($p < 7.14 \times 10^{-3}$) by a double asterisk (**). r_g, genetic correlations.

Shared heritability among oral traits

To understand possible between-trait correlations, we computed genetic correlations using LDSC.[23] Of these, 378 pairs (17.1%) showed nominal evidence of genetic correlation ($p < 0.05$), and 231 pairs (10.4%) remained significant after category-level Bonferroni correction ($p < 7.14 \times 10^{-3}$). As expected, strong positive correlations were observed between clinically and etiologically closely related phenotypes, such as caries and pulp and periapical diseases ($r_g = 0.84$, 95% confidence interval [CI]: [0.78, 0.89], $p = 5.0 \times 10^{-174}$), pulpitis and chronic apical periodontitis ($r_g = 0.91$, 95% CI: [0.82, 0.99], $p = 1.3 \times 10^{-103}$), tooth loss and chronic apical periodontitis ($r_g = 0.91$, 95% CI: [0.76, 1.05], $p = 1.7 \times 10^{-34}$), and attrition and bruxism ($r_g = 0.90$, 95% CI: [0.75, 1.04], $p = 1.7 \times 10^{-33}$). Strong positive correlations were also found between oral lichen planus and oral leukoplakia and other epithelial disorders ($r_g = 0.86$, 95% CI: [0.50, 1.23], $p = 3.3 \times 10^{-6}$), and pulp necrosis and stomatitis ($r_g = 0.90$, 95% CI: [0.35, 1.45], $p = 0.0013$). Conversely, a small number of negative correlations were observed for occlusal phenotypes, such as crossbite and deep bite ($r_g = -0.51$, 95% CI: [−0.78, −0.25], $p = 0.0002$), and crossbite and disto-occlusion ($r_g = -0.66$, 95% CI: [−1.06, −0.25], $p = 0.0014$) (Table S6).

Hierarchical clustering of genetic correlation profiles revealed distinct clusters of oral phenotypes that reflect both anatomical and etiological proximity. An inflammatory cluster included caries, apical periodontitis, and periodontitis; a mucosal cluster grouped oral leukoplakia and lichen planus; an occlusal/functional cluster included TMD, bruxism, and tooth wear; and a structural/dentofacial cluster contained malocclusions and palatinal maxillary canine. These clusters are visualized in Figure 3 and suggest substantial pleiotropy within oral tissues.

Oral and systemic diseases share genetic architecture

To examine links between oral and overall health and disease burden, we calculated genetic correlations between oral phenotypes and 27 common systemic diseases and phenotypes using LDSC. Nominal evidence of genetic correlation ($p < 0.05$) was observed for 419 oral-systemic phenotype pairs (25.4%), suggesting overlapping biological mechanisms that extend beyond oral tissues. Of these, 278 pairs (16.8%) remained significant after category-level Bonferroni correction ($p < 7.14 \times 10^{-3}$; Table S7).

The strongest genetic correlations were observed between chronic laryngitis/laryngotracheitis and gingivitis and periodontal diseases ($r_g = 0.97$, 95% CI: [0.58, 1.36], $p = 1.2 \times 10^{-6}$) and caries ($r_g = 0.94$, 95% CI: [0.58, 1.30], $p = 3.0 \times 10^{-7}$). Several other inflammatory and infectious upper respiratory diseases were also strongly correlated with common oral diseases, forming a cluster as shown in Figure 4. For example, chronic rhinitis, nasopharyngitis, and pharyngitis showed correlation with TMDs ($r_g = 0.74$, 95% CI: [0.60, 0.88], $p = 1.3 \times 10^{-24}$) and diseases of pulp and periapical tissues ($r_g = 0.37$, 95% CI: [0.27, 0.48], $p = 1.8 \times 10^{-12}$). Additionally, genetic correlations were observed between autoimmune and oral diseases, such as seropositive rheumatoid arthritis (MIM: 180300) and caries ($r_g = 0.24$, 95% CI: [0.16, 0.31], $p = 9.4 \times 10^{-10}$).

Chronic complicated periodontitis was genetically correlated with being a current smoker ($r_g = 0.77$, 95% CI: [0.70, 0.84], $p = 1.1 \times 10^{-98}$) and type 2 diabetes (MIM: 125853; $r_g = 0.33$, 95% CI: [0.26, 0.39], $p = 1.1 \times 10^{-24}$), aligning with previous epidemiological evidence linking periodontal inflammation to metabolic dysfunction and smoking.[4,29] A positive correlation was also observed between bruxism and gastroesophageal reflux disease (MIM: 109350; $r_g = 0.51$, 95% CI: [0.38, 0.64], $p = 1.1 \times 10^{-13}$), as described in previous studies (Table S7).[10]

UK Biobank, EstBB, and MVP data support key findings

To validate our GWAS results, we performed replication analyses using summary statistics from 12 corresponding UK Biobank oral phenotypes,[30] 21 MVP phenotypes,[31] and EstBB data for three oral phenotypes (Tables S9–S15). Fine-mapped lead variants and additional coding variants were evaluated in corresponding or category-matched replication phenotypes.

Of the variants available for replication, 30 of 93 (32.3%) showed nominal evidence of replication ($p < 0.05$), while 16 variants were not available in the replication cohorts. Effect estimates between FinnGen and the replication analyses were largely concordant (Figure S4). Replication support was strongest for diseases of dental hard tissue and pulp/periapical tissue, which accounted for half of the replicated variants (15/30).

Most replicated associations were observed in MVP, where 25 variants showed nominal evidence of replication. Eight variants showed replication in more than one external cohort, including variants near *CHRNA5* (MIM: 118505) and *CHRNA3* (MIM: 118503) for chronic complicated periodontitis and near *MTMR3* (MIM: 603558) for pulp necrosis. In the EstBB, the previously unreported missense variant for caries in *USP31* also showed nominal replication. Additional replicated variants included loci near *ASCL5* (MIM: 620809) for hypodontia/oligodontia, *CDH6* (MIM: 603007) for chronic complicated periodontitis, *VMP1* (MIM: 611753) for pulpitis, *FZD9* for attrition, *CLEC16A* (MIM: 611303) for peripheral and oral lichen planus, and *GPNMB* for TMDs (Table S12). Replication power was limited by phenotype availability and smaller numbers of affected individuals in external cohorts, particularly for developmental, dentofacial, and occlusal phenotypes.

Discussion

In this comprehensive genome-wide analysis of 67 oral and craniofacial phenotypes in over 500,000 individuals from the FinnGen study, we observed 102 genome-wide significant loci, of which 45 had not been previously described in the literature for these phenotypes. The phenotypic spectrum analyzed spans dental hard tissues, oral mucosa, periodontal tissues, salivary glands, and craniofacial structures, offering broader coverage than previous studies, which have largely focused on caries and periodontitis in smaller cohorts.[5,6] By combining fine-mapping, HLA imputation, and genetic correlation analyses, our work expands the known genetic architecture of oral diseases and their overlap with systemic conditions.

Coding variants implicate key biological pathways

Among the 102 fine-mapped loci, we observed 14 coding variants, including five previously unreported variants. We identified shared pathways across dental phenotypes including ubiquitination, antigen presentation, lysosomal glycan catabolism, amino acid metabolism, epithelial integrity, and bone formation. For example, a missense variant in *USP31*, associated with caries (rs200486134), encodes a deubiquitinating enzyme linked to NF-κB activation and immune regulation.[32] Other members of the ubiquitin-specific protease family have also been implicated in dental biology; for example, *USP34* (MIM: 615295) has been shown to influence odontogenic differentiation and tooth root morphogenesis through stabilization of NFIC, a transcription factor essential for root development.[33] A variant (rs75826658) in *MANBA*, associated with oral leukoplakia and epithelial disorders, implicates lysosomal β-mannosidase in glycan degradation and epithelial biology.[34] *THNSL2* (rs4129190), associated with hypodontia/oligodontia, encodes threonine synthase-like 2. An alternatively spliced isoform (SOFAT) of *THNSL2* has been identified in human T cells and shown to stimulate IL-6 production and osteoclastogenesis.[35] *GPNMB* (rs11537976), linked here to TMDs, encodes osteoactivin, a transmembrane glycoprotein. It promotes osteoblast differentiation and bone formation *in vitro* and *in vivo* (mouse models) and functions as an immunomodulator.[36,37] *GPNMB* (variant rs199354) has been previously associated with anxiety-independent TMDs in a GWAS-by-subtraction analysis, suggesting possible involvement in bone remodeling or local inflammatory processes.[38] Overall, these findings demonstrate that classical biological processes, particularly those involving ubiquitination, lysosomal glycan catabolism, amino acid metabolism, epithelial integrity, immunity, and bone formation, identified earlier from model organism and human disorder studies, also contribute to common variation in dental and oral phenotypes.

Hypodontia/oligodontia is biologically consistent with a developmental interpretation, as tooth agenesis arises during early odontogenesis. *WNT10A* provides the strongest direct link to tooth development, with prior association to non-syndromic tooth agenesis, including in Arte et al.[27] *FGF9* further supports a role for developmental signaling, as FGF signaling is central to mammalian tooth development and *FGF9* is expressed during key stages of tooth morphogenesis.[28] *THNSL2* is interpreted more cautiously as a gene with plausible relevance to broader developmental or bone-related biology rather than as a

canonical tooth development gene.[35] Together, these findings suggest convergence on developmental signaling mechanisms relevant to tooth formation.

Finnish-enriched variants and insights from a founder population

Several variants were enriched in the Finnish population, reflecting its unique genetic architecture shaped by historical bottlenecks and drift.[15,39] These included variants near *HDGFL1* (encoding a growth factor-like protein implicated in cell proliferation), *LIPA* (encoding lysosomal acid lipase involved in lipid metabolism and inflammatory processes), *NUP42* (encoding a nucleoporin required for mRNA export), and *MBP* (encoding myelin basic protein essential for myelination), associated with peripheral/oral lichen planus, pulpitis, TMDs, and anomalies of dental arch relations, respectively. Such population-enriched variants, some absent in non-Finnish Europeans, may implicate biological pathways not previously linked to oral and craniofacial phenotypes and highlight the value of founder populations for rare variant discovery.

HLA region associations with oral mucosal diseases

Our HLA fine-mapping identified strong and specific associations between class I and class II alleles and diverse oral phenotypes. The strongest signals were for oral and peripheral lichen planus, with *DQB1*05:01* and *DQA1*01:01* showing large positive associations and *DQA1*01:02* showing a protective association with diseases of the lip and oral mucosa, as described earlier.[11] Inflammatory dental phenotypes, including pulpitis and chronic apical periodontitis, were associated with *DRB4*01:03* and *DQB1*03:01*, respectively, and attrition was associated with *B*18:01*. These findings are consistent with previous studies linking HLA variation to lichen planus and pulpal diseases, suggesting that antigen presentation may influence susceptibility to a broad range of oral diseases.[11,13]

Genetic correlations among oral traits and with systemic conditions

Our genome-wide genetic correlation analyses revealed extensive overlap both within oral phenotypes and between oral and systemic diseases. As expected, oral phenotypes that are clinically and etiologically linked showed the strongest positive correlations, such as caries with pulp and periapical diseases, pulpitis with chronic apical periodontitis, and attrition with bruxism. Mucosal disorders, including oral lichen planus and oral leukoplakia, also displayed strong genetic overlap, consistent with previous evidence that both conditions involve epithelial and immune dysregulation.[40] Conversely, some malocclusion traits exhibited negative correlations, for example, crossbite with deep bite, reflecting contrasting dentofacial patterns that are less commonly observed together. Hierarchical clustering of correlation profiles delineated four main oral disease clusters: an inflammatory dental cluster (e.g., caries, apical periodontitis, and periodontitis), a mucosal cluster (e.g., oral leukoplakia and lichen planus), an occlusal/functional cluster (e.g., TMDs, bruxism, and tooth wear), and a structural/dentofacial cluster (e.g., malocclusions).

Extending the analysis to 27 common systemic diseases and traits revealed that nearly one-quarter of oral-systemic pairs were genetically correlated. The strongest overlaps were observed between chronic laryngitis/laryngotracheitis and both gingivitis/periodontal disease and caries and between upper respiratory infections and TMDs or diseases of pulp and periapical tissues. Autoimmune conditions such as seropositive rheumatoid arthritis showed positive correlations with caries, in line with prior evidence linking systemic immune dysregulation to oral pathology.[4] Chronic complicated periodontitis also correlated strongly with current smoking and type 2 diabetes, reinforcing well-established epidemiological links between periodontal inflammation, smoking, and metabolic dysfunction.[41,42] Furthermore, bruxism was genetically correlated with gastroesophageal reflux disease, echoing previous clinical observations.[43]

Caries and related pulpal/apical phenotypes provide a clinically intuitive and biologically coherent example of a progressive odontogenic inflammatory continuum. Rather than representing isolated registry endpoints, caries, pulpitis, and chronic apical periodontitis can be viewed as consecutive stages of tissue injury and host response, extending from dental hard tissue breakdown to pulpal inflammation and persistent periapical lesions. This interpretation is consistent with prior literature showing that caries progression is closely linked to the development of pulpal disease[44] and that apical periodontitis arises from pulpal disease and is shaped by host inflammatory and immune responses.[45–47] In this context, the combination of strong genetic correlations, phenotype-specific association signals, and HLA associations in our study suggests that this cluster reflects not only structural damage but also shared inflammatory and immune mechanisms.

Limitations

This study has several limitations. Registry-based phenotyping may lead to misclassification or underdetection of certain diagnoses, particularly rare or subclinical conditions. Additionally, defining GWAS controls based on the absence of the corresponding ICD code may reduce phenotypic specificity and limit the detection of variants that are shared across related phenotypes. Although the Finnish founder population enhances power to detect low-frequency variants, generalizability of population-specific signals may be limited, and replication in non-Finnish cohorts is more challenging. In addition, some effect estimates, particularly for rarer phenotypes, were relatively large and should be interpreted with caution. Direct comparison with previously reported effect sizes is

difficult for many of these phenotypes because comparable GWAS data remain limited and phenotype definitions often differ across studies. Larger observed effects may partly reflect limited case counts, winner's curse, and Finnish enrichment of some associated variants. Finally, while our analyses suggest mechanistic hypotheses, functional validation of implicated variants is needed to establish causal relationships.

Data and code availability

FinnGen individual-level data are not publicly available but may be accessed through the Finnish biobanks' FINBB portal (https://finbb.fi/en/) and the Fingenious services (https://site.fingenious.fi/en/) managed by FINBB, subject to the relevant data access procedures. Publicly available FinnGen release 12 (R12) summary statistics can be accessed through the FinnGen results portal (https://r12.finngen.fi) and data download services. FinnGen Regenie, fine-mapping, and LDSC pipelines are publicly available through the FinnGen GitHub organization (https://github.com/FINNGEN). UK Biobank and MVP replication summary statistics used in this study were obtained from previously published GWAS, as listed in Tables S9 and S11. EstBB replication data used in this study are not publicly available due to ethical and legal restrictions but may be available from the corresponding authors upon reasonable request, subject to applicable regulations.

Acknowledgments

We want to acknowledge the participants and investigators of the FinnGen study. The FinnGen project is funded by two grants from Business Finland (HUS 4685/31/2016 and UH 4386/31/2016) and the following industry partners: AbbVie, Inc.; AstraZeneca UK Ltd.; Biogen MA, Inc.; Bristol-Myers Squibb, Inc. (and Celgene Corporation and Celgene International II Sàrl); Genentech; Merck Sharp & Dohme LLC; Pfizer, Inc.; GlaxoSmithKline Intellectual Property Development Ltd.; Sanofi US Services, Inc.; Maze Therapeutics, Inc.; Johnson & Johnson Innovative Medicine, Inc.; Novartis AG; Boehringer Ingelheim International GmbH; and Bayer AG. The following biobanks are acknowledged for delivering biobank samples to FinnGen: Auria Biobank (www.auria.fi/biopankki), THL Biobank (www.thl.fi/biobank), Helsinki Biobank (www.helsinginbiopankki.fi), Biobank Borealis of Northern Finland (https://www.ppshp.fi/Tutkimus-ja-opetus/Biopankki/Pages/Biobank-Borealis-briefly-in-English.aspx), Finnish Clinical Biobank Tampere (www.tays.fi/en-US/Research_and_development/Finnish_Clinical_Biobank_Tampere), Biobank of Eastern Finland (www.ita-suomenbiopankki.fi/en), Central Finland Biobank (www.ksshp.fi/fi-FI/Potilaalle/Biopankki), Finnish Red Cross Blood Service Biobank (www.veripalvelu.fi/verenluovutus/biopankkitoiminta), Terveystalo Biobank (www.terveystalo.com/fi/Yritystietoa/Terveystalo-Biopankki/Biopankki/), and Arctic Biobank (https://www.oulu.fi/en/university/faculties-and-units/faculty-medicine/northern-finland-birth-cohorts-and-arctic-biobank). All Finnish Biobanks are members of the BBMRI.fi infrastructure (https://www.bbmri-eric.eu/national-nodes/finland/). The Finnish Biobank Cooperative (FINBB; https://finbb.fi/) is the coordinator of BBMRI-ERIC operations in Finland. The Finnish biobank data can be accessed through the Fingenious services (https://site.fingenious.fi/en/) managed by FINBB. We want to acknowledge the participants of the EstBB for their contributions. The Estonian Genome Center analyses were partially carried out in the High Performance Computing Center, University of Tartu. The EstBB Research Team was responsible for data collection, genotyping, quality control, and imputation and consisted of Andres Metspalu (andres.metspalu@ut.ee), Mait Metspalu (mait.metspalu@ut.ee), Lili Milani (lili.milani@ut.ee), Reedik Mägi (reedik.magi@ut.ee), Mari Nelis (mari.nelis@ut.ee), Tõnu Esko (tonu.esko@ut.ee), and Georgi Hudjashov (georgi.hudjashov@ut.ee). The work of the Estonian Genome Center, University of Tartu, was funded by Estonian Research Council grant PRG1291 and Roadmap II project number TT17 and by the University of Tartu Development Fund bridging grant PLTGIARENG24. This work was supported by Finska Läkaresällskapet (to K.K. and N.M.) and the Academy of Finland (grant number 355567 to N.M.).

Declaration of interests

The authors declare no competing interests.

Supplemental information

Supplemental information can be found online at https://doi.org/10.1016/j.xhgg.2026.100633.

Web resources

Fingenious services, https://site.fingenious.fi/en/
FinnGen fine-mapping pipeline, https://github.com/FINNGEN/finemapping-pipeline
FinnGen LDSC pipeline, https://github.com/FINNGEN/LDSC
FinnGen Regenie pipeline, https://github.com/FINNGEN/regenie-pipelines/
FinnGen Release 12 results portal, https://r12.finngen.fi
Finnish biobanks' FINBB portal, https://finbb.fi/en/
NHGRI-EBI GWAS Catalog, https://www.ebi.ac.uk/gwas/
OMIM, http://www.omim.org/
SiSu v.4 reference panel, http://www.sisuproject.fi/

Received: February 12, 2026
Accepted: June 11, 2026

References

1. GBD 2017 Oral Disorders Collaborators, Bernabe, E., Marcenes, W., Hernandez, C.R., Bailey, J., Abreu, L.G., Alipour, V., Amini, S., Arabloo, J., and Arefi, Z. (2020). Global, Regional, and National Levels and Trends in Burden of Oral Conditions from 1990 to 2017: A Systematic Analysis for the Global Burden of Disease 2017 Study. J. Dent. Res. *99*, 362–373.
2. Natarajan, P., Madanian, S., and Marshall, S. (2025). Investigating the link between oral health conditions and systemic diseases: A cross-sectional analysis. Sci. Rep. *15*, 10476.
3. Al-Marzooq, F.I., and Christidis, N. (2025). The interconnection of oral and systemic health. Sci. Rep. *15*, 14931.
4. Botelho, J., Mascarenhas, P., Viana, J., Proença, L., Orlandi, M., Leira, Y., Chambrone, L., Mendes, J.J., and Machado, V. (2022). An umbrella review of the evidence linking oral health and systemic noncommunicable diseases. Nat. Commun. *13*, 7614.
5. Shungin, D., Haworth, S., Divaris, K., Agler, C.S., Kamatani, Y., Keun Lee, M., Grinde, K., Hindy, G., Alaraudanjoki, V.,

Pesonen, P., et al. (2019). Genome-wide analysis of dental caries and periodontitis combining clinical and self-reported data. Nat. Commun. *10*, 2773.
6. Munz, M., Willenborg, C., Richter, G.M., Jockel-Schneider, Y., Graetz, C., Staufenbiel, I., Wellmann, J., Berger, K., Krone, B., Hoffmann, P., and Van Der Velde, N. (2017). A genome-wide association study identifies nucleotide variants at SIGLEC5 and DEFA1A3 as risk loci for periodontitis. Hum. Mol. Genet. *26*, 2577–2588.
7. Munz, M., Richter, G.M., Loos, B.G., Jepsen, S., Divaris, K., Offenbacher, S., Teumer, A., Holtfreter, B., Kocher, T., Bruckmann, C., et al. (2019). Meta-analysis of genome-wide association studies of aggressive and chronic periodontitis identifies two novel risk loci. Eur. J. Hum. Genet. *27*, 102–113.
8. Salminen, A., Hyvärinen, K., Ritari, J., Caetano, A., Kambur, O., Mäntylä, P., Yilmaz, M., Sinisalo, J., FinnGen, Palotie, A., and Daly, M. (2025). Genetic Loci Associated With Periodontitis: The FinnGen Study Based on National Health Registers. J Clin Periodontol *52*, 1263–1275.
9. Saarentaus, E.C., Karjalainen, J., Rämö, J.T., Kiiskinen, T., Havulinna, A.S., Mehtonen, J., Hautakangas, H., Ruotsalainen, S., Tamlander, M., Mars, N., et al. (2023). Inflammatory and infectious upper respiratory diseases associate with 41 genomic loci and type 2 inflammation. Nat. Commun. *14*, 83.
10. Strausz, T., Strausz, S., FinnGen, Palotie, T., Ahlberg, J., and Ollila, H.M. (2023). Genetic analysis of probable sleep bruxism and its associations with clinical and behavioral traits. Sleep *46*, zsad107.
11. Reeve, M.P., Vehviläinen, M., Luo, S., Ritari, J., Karjalainen, J., Gracia-Tabuenca, J., Mehtonen, J., Padmanabhuni, S.S., Kolosov, N., Artomov, M., et al. (2024). Oral and non-oral lichen planus show genetic heterogeneity and differential risk for autoimmune disease and oral cancer. Am. J. Hum. Genet. *111*, 1047–1060.
12. Rahimov, F., Nieminen, P., Kumari, P., Juuri, E., Nikopensius, T., Paraiso, K., German, J., Karvanen, A., Kals, M., Elnahas, A.G., et al. (2024). High incidence and geographic distribution of cleft palate in Finland are associated with the IRF6 gene. Nat. Commun. *15*, 9568.
13. Salminen, A., Hyvärinen, K., Ritari, J., Leppilahti, J.M., Palotie, U., Vuollo, V., Kambur, O., FinnGen; and Estonian Biobank Research Team, and Reis, K. (2025). Genome-wide association study of pulpal and apical diseases. Nat. Commun. *16*, 6774.
14. Juuri, E., Strausz, S., Hasan, M.R., Hongqiang, M., Jónsson, T., Thordarsson, Á., Auðólfsson, G., Gudbjartsson, D., Stefansson, H., Nieminen, P., et al. (2025). Variants Near NOG and SOX9 Are Associated with Mandibular Retrognathia. J. Dent. Res. 220345251397375.
15. Kurki, M.I., Karjalainen, J., Palta, P., Sipilä, T.P., Kristiansson, K., Donner, K.M., Reeve, M.P., Laivuori, H., Aavikko, M., Kaunisto, M.A., et al. (2023). FinnGen provides genetic insights from a well-phenotyped isolated population. Nature *613*, 508–518.
16. Mbatchou, J., Barnard, L., Backman, J., Marcketta, A., Kosmicki, J.A., Ziyatdinov, A., Benner, C., O'Dushlaine, C., Barber, M., Boutkov, B., et al. (2021). Computationally efficient whole-genome regression for quantitative and binary traits. Nat. Genet. *53*, 1097–1103.
17. R Core Team (2026). R: A Language and Environment for Statistical Computing (R Foundation for Statistical Computing). https://www.R-project.org/.
18. Cerezo, M., Sollis, E., Ji, Y., Lewis, E., Abid, A., Bircan, K.O., Hall, P., Hayhurst, J., John, S., Mosaku, A., et al. (2025). The NHGRI-EBI GWAS Catalog: standards for reusability, sustainability and diversity. Nucleic Acids Res. *53*, D998–D1005.
19. Dyer, S.C., Austine-Orimoloye, O., Azov, A.G., Barba, M., Barnes, I., Barrera-Enriquez, V.P., Becker, A., Bennett, R., Beracochea, M., Berry, A., et al. (2025). Ensembl 2025. Nucleic Acids Res. *53*, D948–D957.
20. Online Mendelian Inheritance in Man (2026). OMIM. McKusick-Nathans Institute of Genetic Medicine (Johns Hopkins University). https://omim.org/.
21. Chen, S., Francioli, L.C., Goodrich, J.K., Collins, R.L., Kanai, M., Wang, Q., Alföldi, J., Watts, N.A., Vittal, C., Gauthier, L.D., et al. (2024). A genomic mutational constraint map using variation in 76,156 human genomes. Nature *625*, 92–100.
22. Ritari, J., Hyvärinen, K., Clancy, J., Koskela, S., FinnGen, and Partanen, J. (2020). Increasing accuracy of HLA imputation by a population-specific reference panel in a FinnGen biobank cohort. NAR Genom. Bioinform. *2*, lqaa030.
23. Bulik-Sullivan, B.K., Loh, P.R., Finucane, H.K., Ripke, S., Yang, J., Schizophrenia Working Group of the Psychiatric Genomics Consortium, Patterson, N., Daly, M.J., Price, A.L., and Neale, B.M. (2015). LD Score regression distinguishes confounding from polygenicity in genome-wide association studies. Nat. Genet. *47*, 291–295.
24. Kolde, R. (2025). pheatmap: Pretty Heatmaps (1.0.13). https://CRAN.R-project.org/package=pheatmap.
25. Milani, L., Alver, M., Laur, S., Reisberg, S., Haller, T., Aasmets, O., Abner, E., Alavere, H., Allik, A., Annilo, T., et al. (2025). The Estonian Biobank's journey from biobanking to personalized medicine. Nat. Commun. *16*, 3270.
26. Leitsalu, L., Haller, T., Esko, T., Tammesoo, M.L., Alavere, H., Snieder, H., Perola, M., Ng, P.C., Mägi, R., Milani, L., et al. (2015). Cohort Profile: Estonian Biobank of the Estonian Genome Center, University of Tartu. Int. J. Epidemiol. *44*, 1137–1147.
27. Arte, S., Parmanen, S., Pirinen, S., Alaluusua, S., and Nieminen, P. (2013). Candidate gene analysis of tooth agenesis identifies novel mutations in six genes and suggests significant role for WNT and EDA signaling and allele combinations. PLoS One *8*, e73705.
28. Porntaveetus, T., Otsuka-Tanaka, Y., Basson, M.A., Moon, A.M., Sharpe, P.T., and Ohazama, A. (2011). Expression of fibroblast growth factors (Fgfs) in murine tooth development. J. Anat. *218*, 534–543.
29. Leite, F.R.M., Nascimento, G.G., Scheutz, F., and López, R. (2018). Effect of Smoking on Periodontitis: A Systematic Review and Meta-regression. Am. J. Prev. Med. *54*, 831–841.
30. Zhou, W., Nielsen, J.B., Fritsche, L.G., Dey, R., Gabrielsen, M.E., Wolford, B.N., LeFaive, J., VandeHaar, P., Gagliano, S.A., Gifford, A., et al. (2018). Efficiently controlling for case-control imbalance and sample relatedness in large-scale genetic association studies. Nat. Genet. *50*, 1335–1341.
31. Verma, A., Huffman, J.E., Rodriguez, A., Conery, M., Liu, M., Ho, Y.L., Kim, Y., Heise, D.A., Guare, L., Panickan, V.A., et al. (2024). Diversity and scale: Genetic architecture of 2068 traits in the VA Million Veteran Program. Science *385*, eadj1182.
32. Tzimas, C., Michailidou, G., Arsenakis, M., Kieff, E., Mosialos, G., and Hatzivassiliou, E.G. (2006). Human ubiquitin specific protease 31 is a deubiquitinating enzyme implicated in activation of nuclear factor-kappaB. Cell. Signal. *18*, 83–92.

33. Jiang, S., Sheng, R., Qi, X., Wang, J., Guo, Y., and Yuan, Q. (2021). USP34 regulates tooth root morphogenesis by stabilizing NFIC. Int. J. Oral Sci. *13*, 7.
34. National Center for Biotechnology Information (NCBI) (2025). MANBA Mannosidase Beta [Homo sapiens (Human)] (NCBI). https://www.ncbi.nlm.nih.gov/gene/4126.
35. Rifas, L., and Weitzmann, M.N. (2009). A novel T cell cytokine, secreted osteoclastogenic factor of activated T cells, induces osteoclast formation in a RANKL-independent manner. Arthritis Rheum. *60*, 3324–3335.
36. Abdelmagid, S.M., Belcher, J.Y., Moussa, F.M., Lababidi, S.L., Sondag, G.R., Novak, K.M., Sanyurah, A.S., Frara, N.A., Razmpour, R., Del Carpio-Cano, F.E., and Safadi, F.F. (2014). Mutation in osteoactivin decreases bone formation in vivo and osteoblast differentiation in vitro. Am. J. Pathol. *184*, 697–713.
37. Saade, M., Araujo de Souza, G., Scavone, C., and Kinoshita, P.F. (2021). The Role of GPNMB in Inflammation. Front. Immunol. *12*, 674739.
38. Cao, Y., Yang, X., Svensson, P., Chung Wen, R.W., Han Sng, T.J., Islam, I., Han, W., Feng, X., Hou, B., Li, Y., and Zheng, L. (2026). Genetic subtraction reveals divergent pathways and targets in anxiety-related and anxiety-independent TMD. J. Headache Pain *27*, 105.
39. Lim, E.T., Würtz, P., Havulinna, A.S., Palta, P., Tukiainen, T., Rehnström, K., Esko, T., Mägi, R., Inouye, M., Lappalainen, T., et al. (2014). Distribution and medical impact of loss-of-function variants in the Finnish founder population. PLoS Genet. *10*, e1004494.
40. Binnie, R., Dobson, M.L., Chrystal, A., and Hijazi, K. (2024). Oral lichen planus and lichenoid lesions - challenges and pitfalls for the general dental practitioner. Br. Dent. J. *236*, 285–292.
41. Tomar, S.L., and Asma, S. (2000). Smoking-Attributable Periodontitis in the United States: Findings From NHANES III. J. Periodontol. *71*, 743–751.
42. Wu, C.Z., Yuan, Y.H., Liu, H.H., Li, S.S., Zhang, B.W., Chen, W., An, Z.J., Chen, S.Y., Wu, Y.Z., Han, B., et al. (2020). Epidemiologic relationship between periodontitis and type 2 diabetes mellitus. BMC Oral Health *20*, 204.
43. Wetselaar, P., Manfredini, D., Ahlberg, J., Johansson, A., Aarab, G., Papagianni, C.E., Reyes Sevilla, M., Koutris, M., and Lobbezoo, F. (2019). Associations between tooth wear and dental sleep disorders: A narrative overview. J. Oral Rehabil. *46*, 765–775.
44. Zero, D.T., Zandona, A.F., Vail, M.M., and Spolnik, K.J. (2011). Dental caries and pulpal disease. Dent. Clin. North Am. *55*, 29–46.
45. Graunaite, I., Lodiene, G., and Maciulskiene, V. (2012). Pathogenesis of apical periodontitis: a literature review. J. Oral Maxillofac. Res. *2*, e1.
46. Wen, Y.H., Lin, Y.X., Zhou, L., Lin, C., and Zhang, L. (2024). The immune landscape in apical periodontitis: From mechanism to therapy. Int. Endod. J. *57*, 1526–1545.
47. Márton, I.J., and Kiss, C. (2000). Protective and destructive immune reactions in apical periodontitis. Oral Microbiol. Immunol. *15*, 139–150.

HGGA, Volume 7

Supplemental information

Genetic architecture of 67 oral diseases

and their links to systemic diseases

Kirika Karppinen, Hanna M. Ollila, Kanwal Batool, FinnGen, Estonian Biobank Research Team, Erik Abner, David P. Rice, Aarno Palotie, Tuula Palotie, Samuli Ripatti, Nina Mars, and Satu Strausz

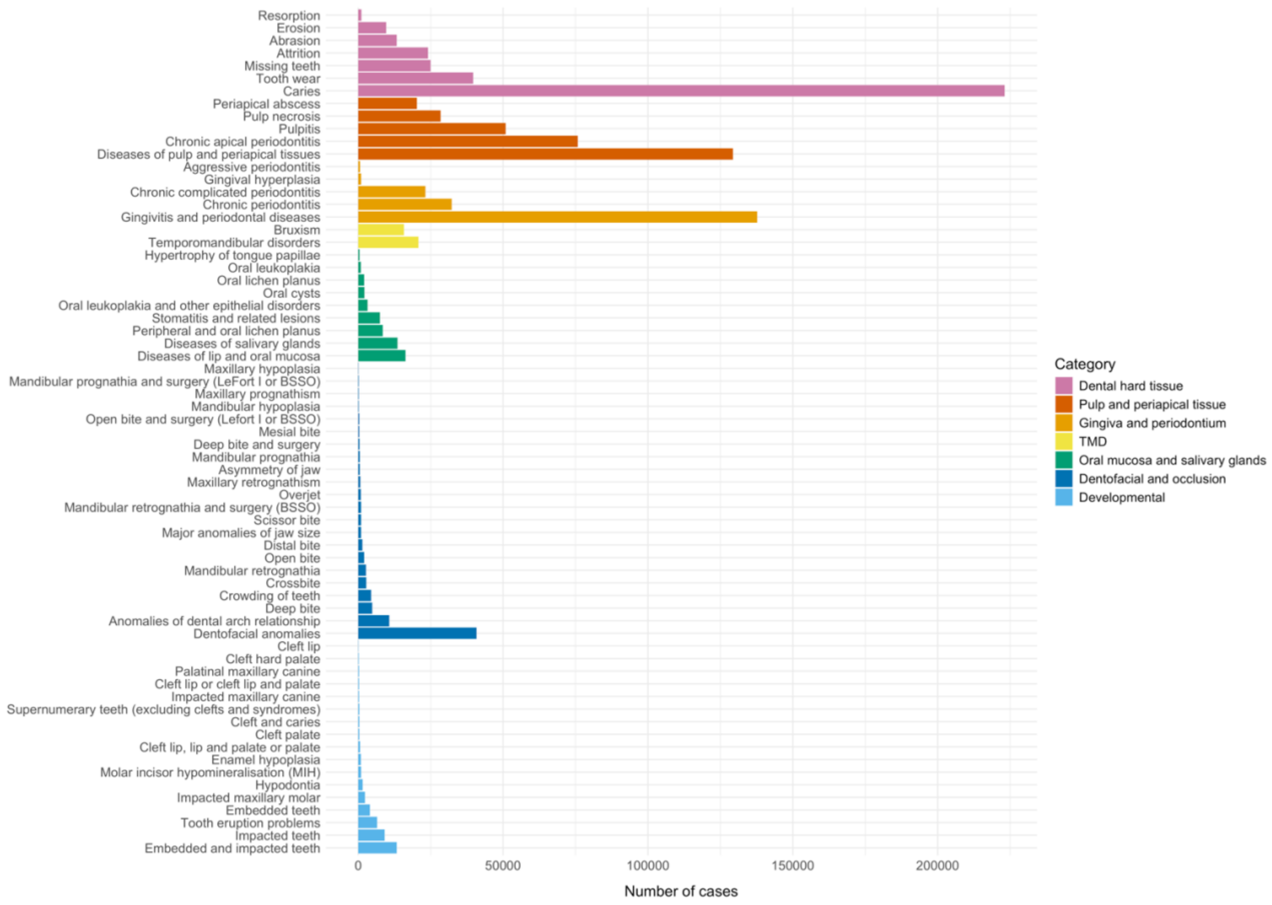

Figure S1. Number of cases of all oral and craniofacial phenotypes. *Phenotypes are organized by phenotype category.*

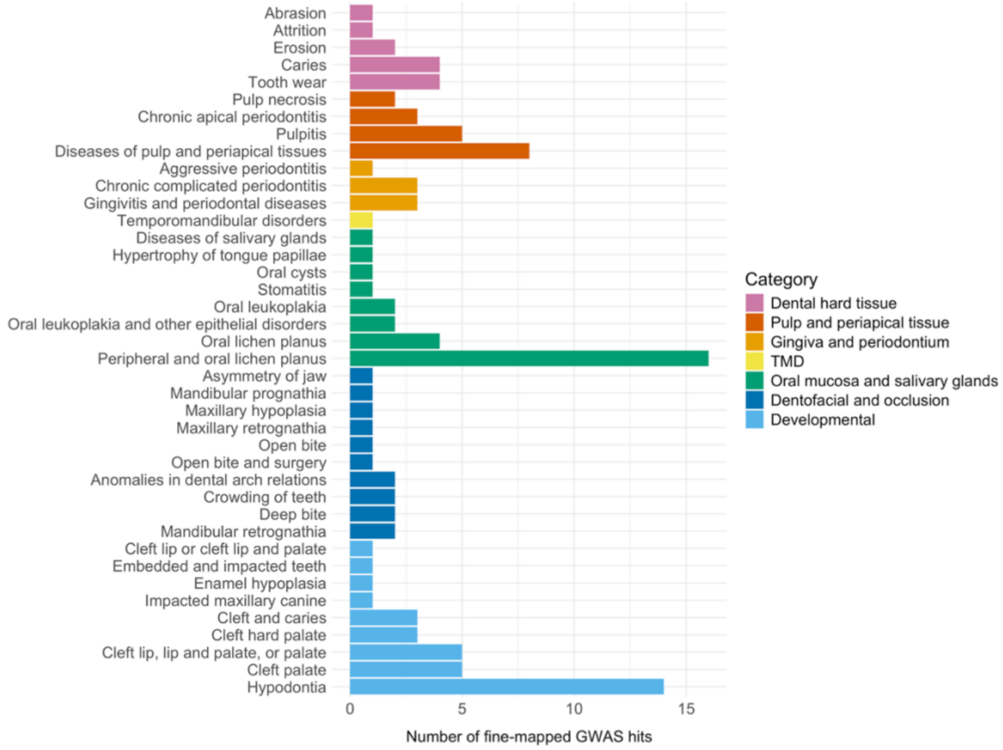

Figure S2. Number of fine-mapped GWAS association per oral and craniofacial phenotype. *Phenotypes are organized by phenotype category.*

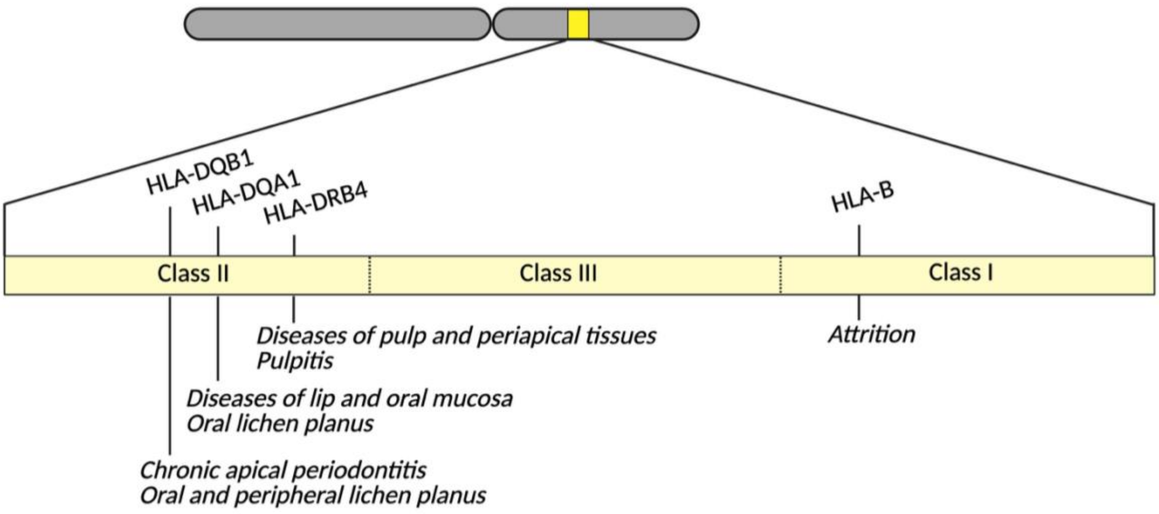

Figure S3. Association of oral phenotypes with HLA alleles according to HLA fine-mapping results. *The approximate positions of the associated HLA alleles within the HLA region are shown. The figure is not drawn to scale.*

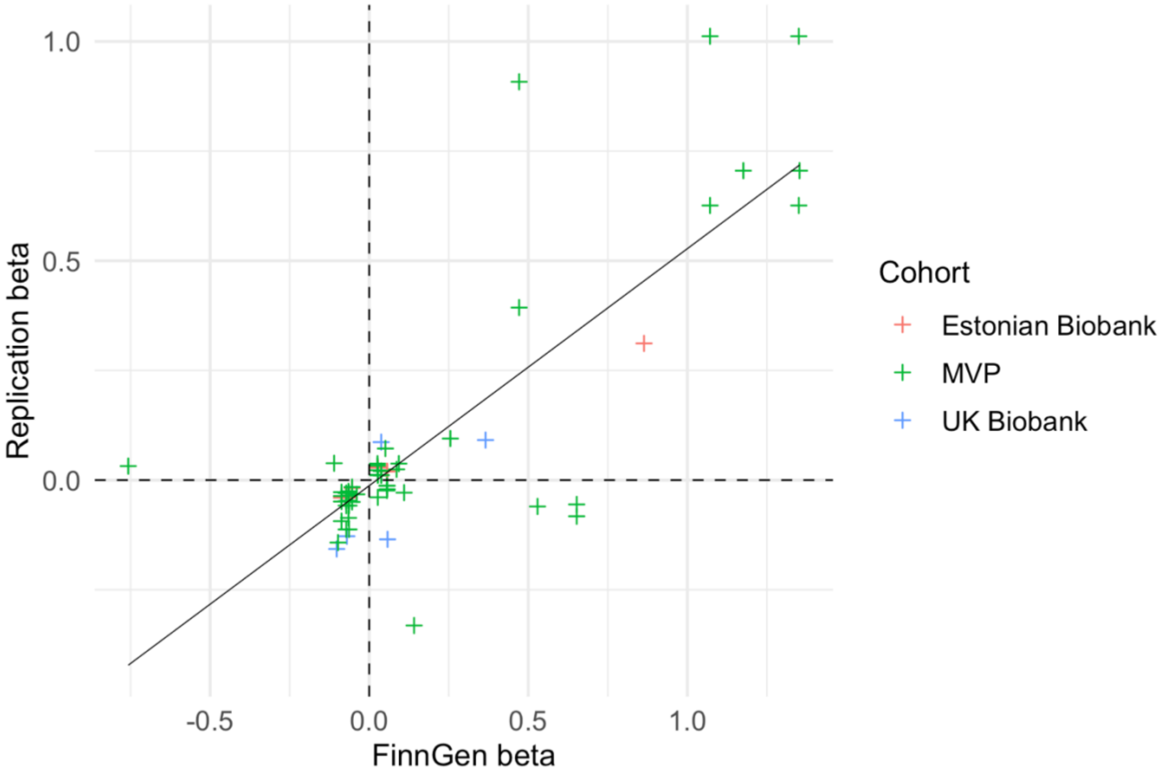

Figure S4. Comparison of effect size estimates between discovery and replication analyses. *Each point represents a successfully replicated variant-phenotype association, with FinnGen GWAS beta estimates plotted against replication estimates across Estonian Biobank, MVP and UK Biobank cohorts. The overall linear fit is shown by the regression line.*

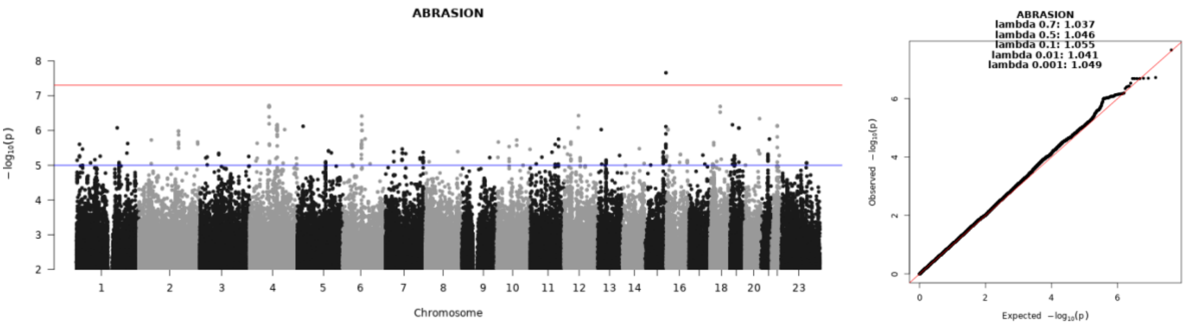

Figure S5. Manhattan and QQ-plot of phenotype Abrasion.

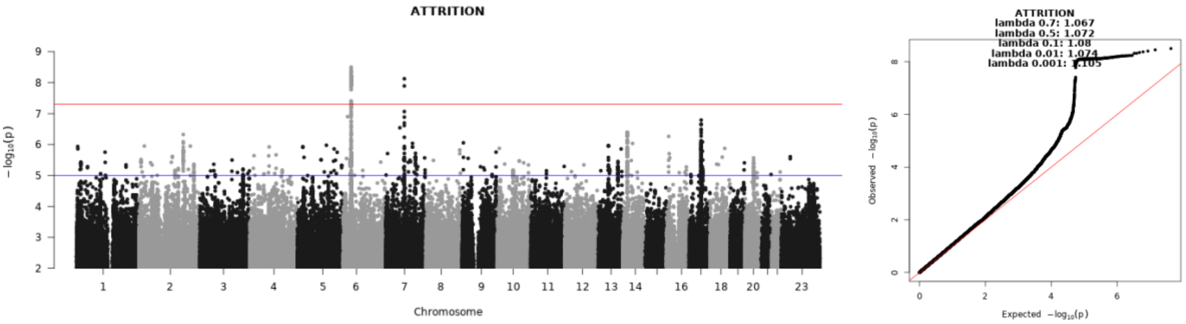

Figure S6. Manhattan and QQ-plot of phenotype Attrition.

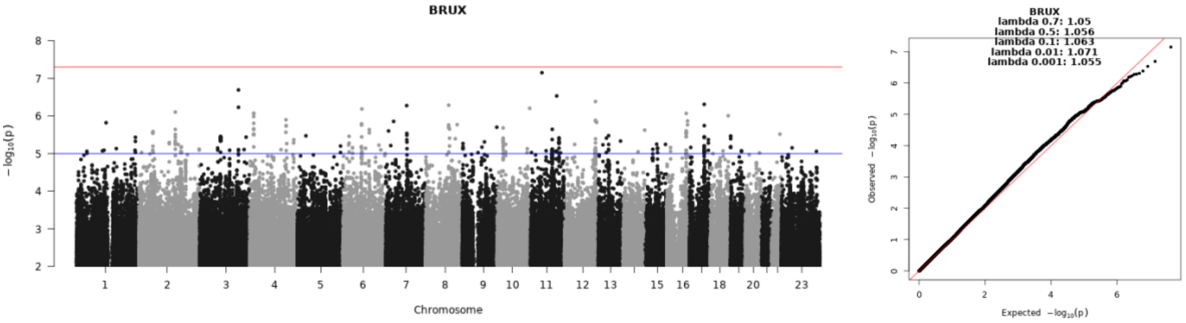

Figure S7. Manhattan and QQ-plot of phenotype Bruxism.

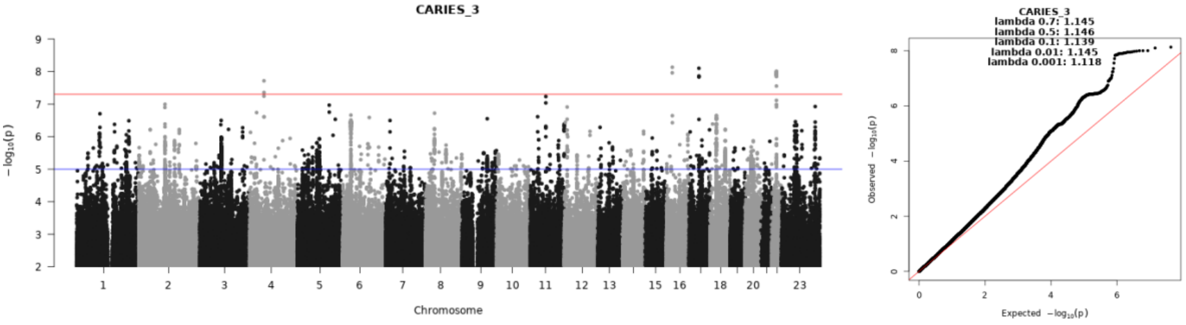

Figure S8. Manhattan and QQ-plot of phenotype Caries.

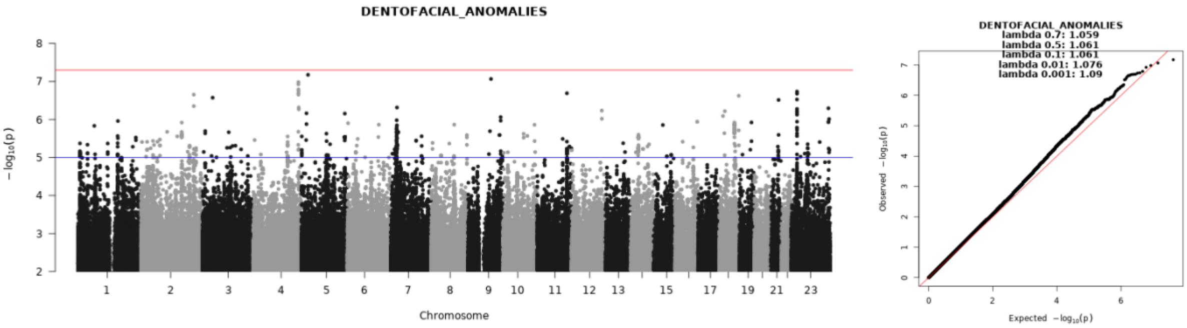

Figure S9. Manhattan and QQ-plot of phenotype Dentofacial anomalies.

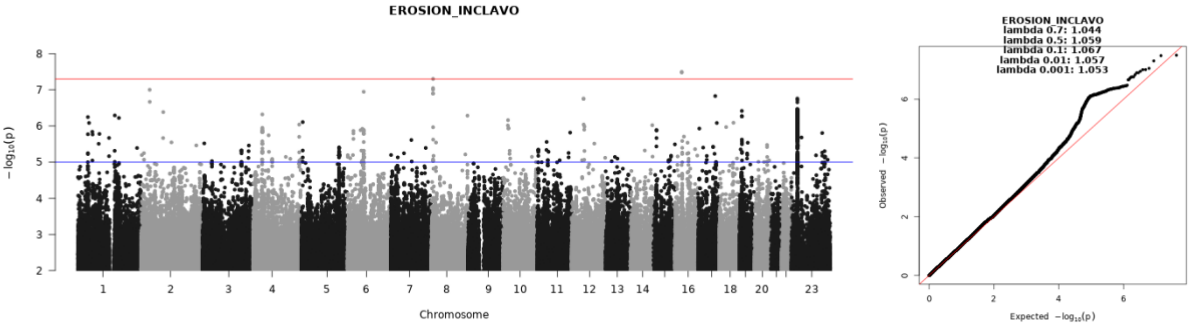

Figure S10. Manhattan and QQ-plot of phenotype Erosion.

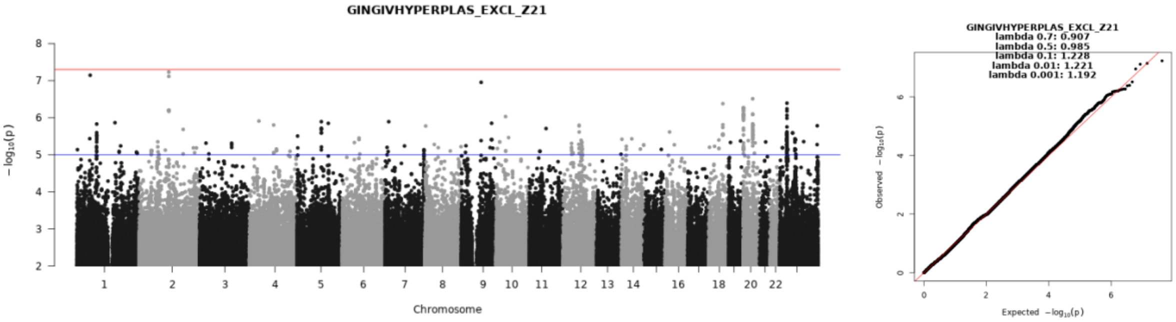

Figure S11. Manhattan and QQ-plot of phenotype Gingival hyperplasia.

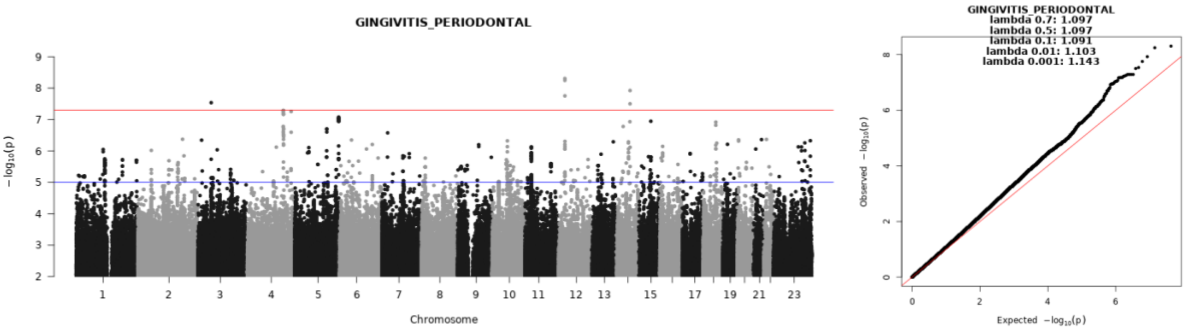

Figure S12. Manhattan and QQ-plot of phenotype Gingivitis and periodontal diseases.

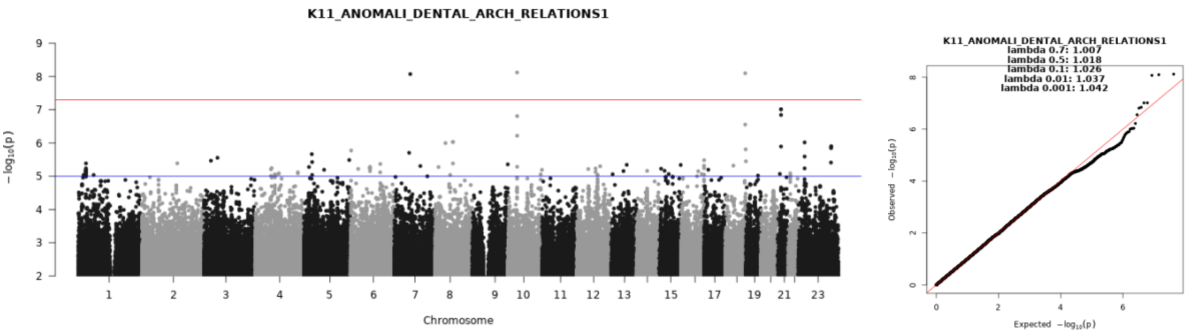

Figure S13. Manhattan and QQ-plot of phenotype Anomalies of dental arch relationship.

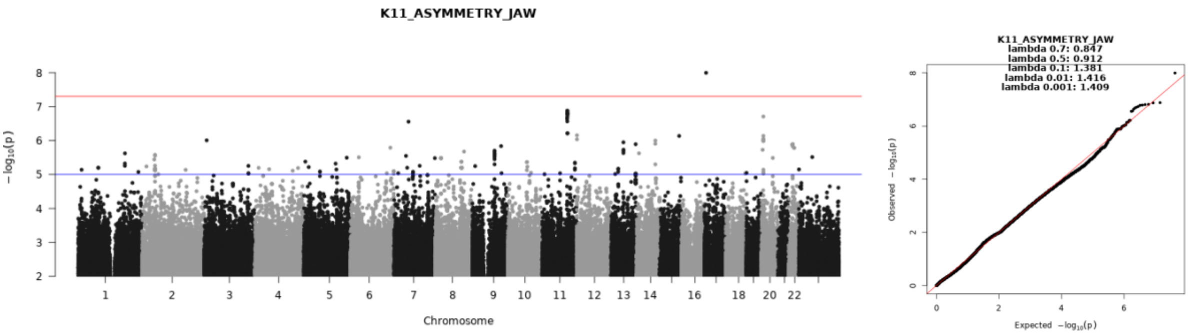

Figure S14. Manhattan and QQ-plot of phenotype Asymmetry of jaw.

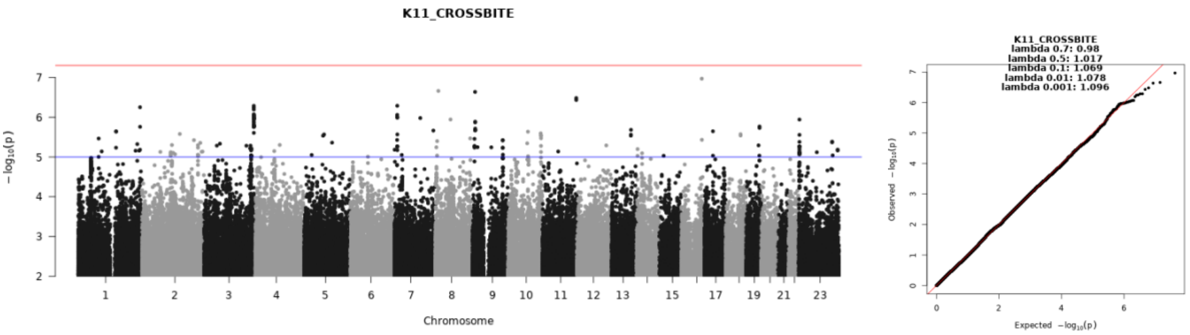

Figure S15. Manhattan and QQ-plot of phenotype Crossbite.

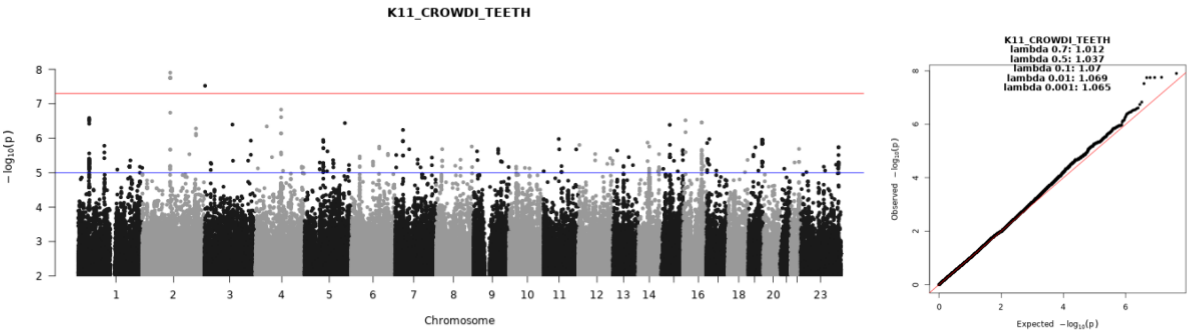

Figure S16. Manhattan and QQ-plot of phenotype Crowding of teeth.

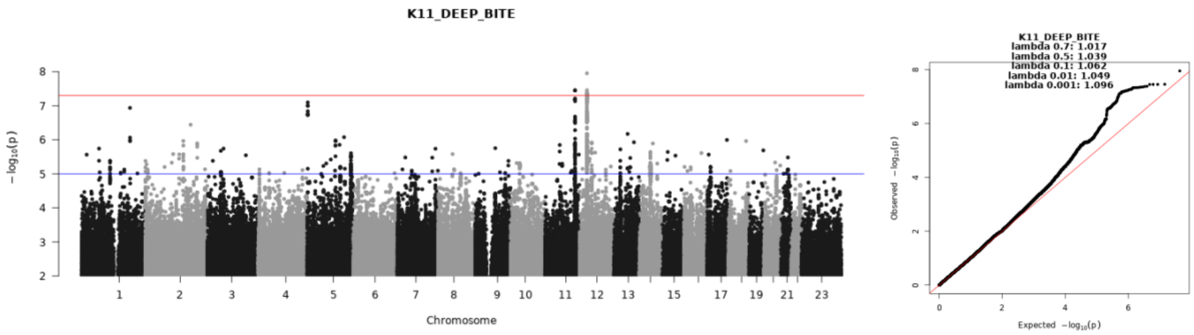

Figure S17. Manhattan and QQ-plot of phenotype Deep bite.

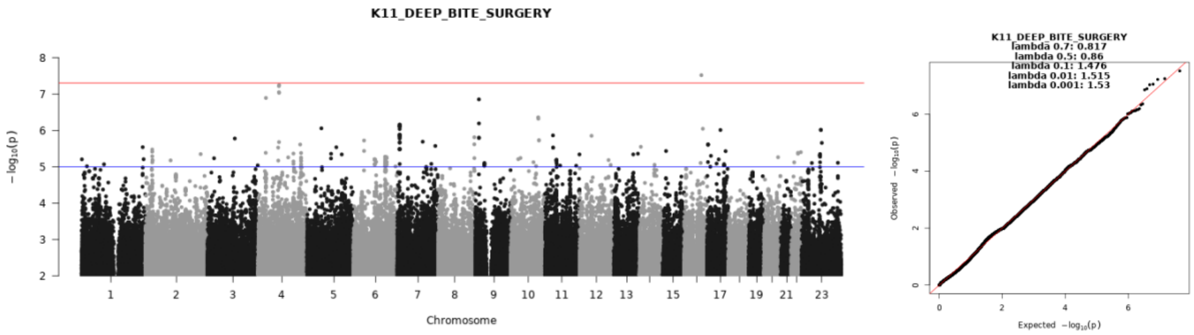

Figure S18. Manhattan and QQ-plot of phenotype Deep bite that required surgery.

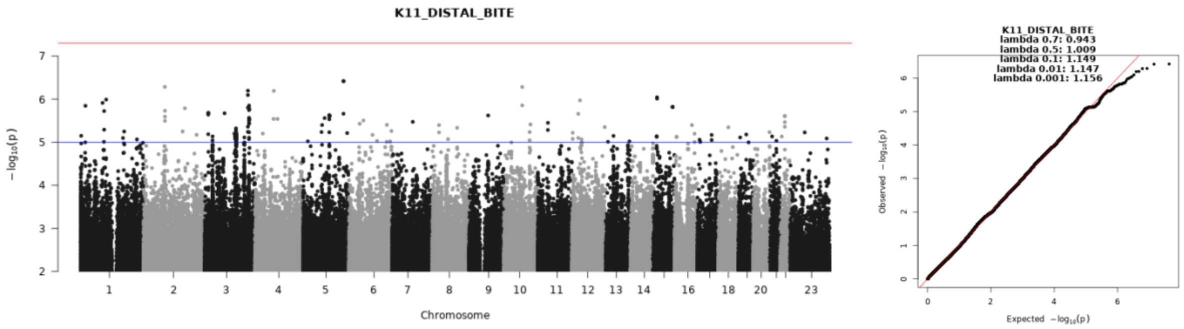

Figure S19. Manhattan and QQ-plot of phenotype Disto-occlusion.

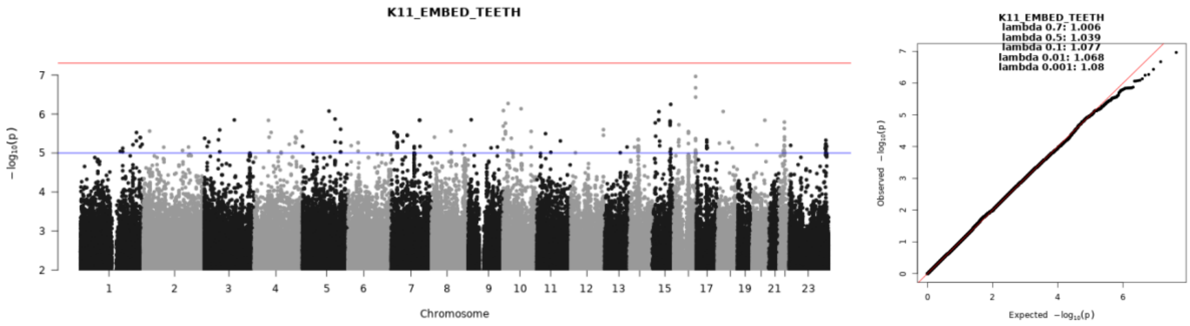

Figure S20. Manhattan and QQ-plot of phenotype Embedded teeth.

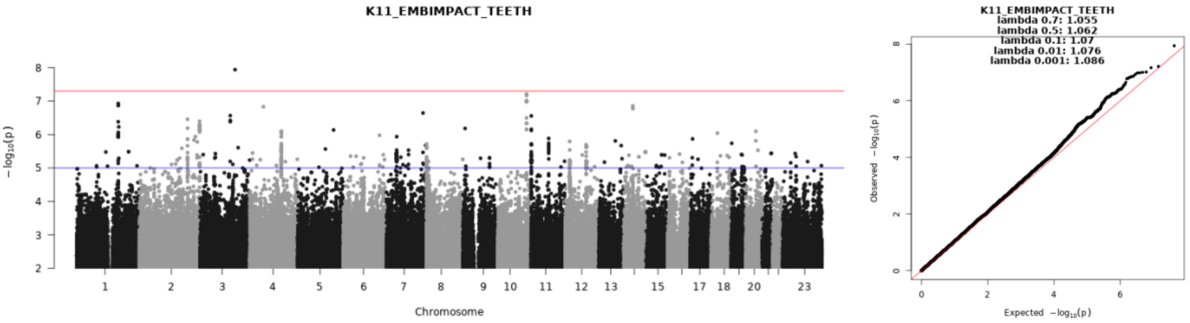

Figure S21. Manhattan and QQ-plot of phenotype Embedded and impacted teeth.

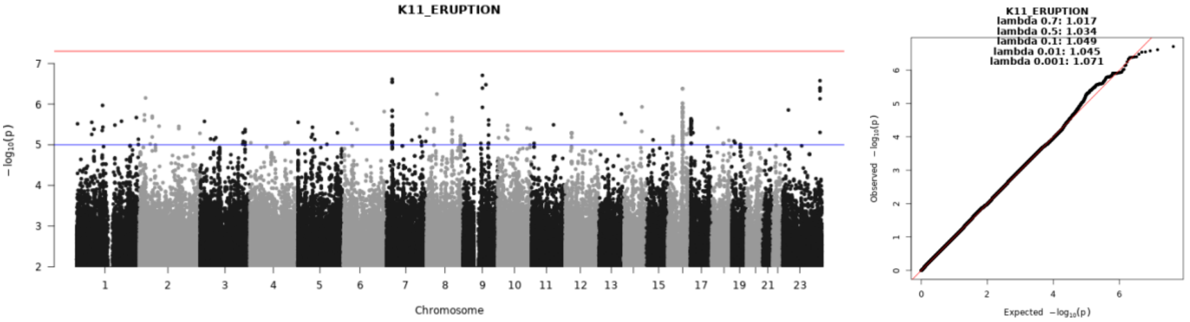

Figure S22. Manhattan and QQ-plot of phenotype Tooth eruption problems.

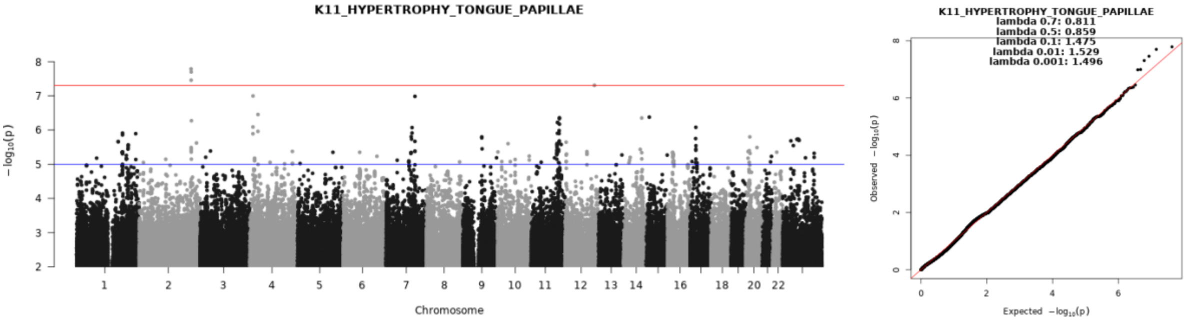

Figure S23. Manhattan and QQ-plot of phenotype Hypertrophy of tongue papillae.

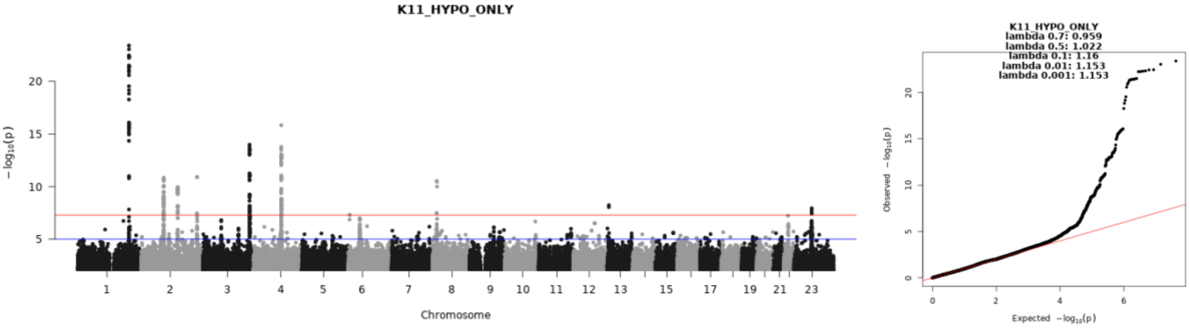

Figure S24. Manhattan and QQ-plot of phenotype Hypodontia or oligodontia.

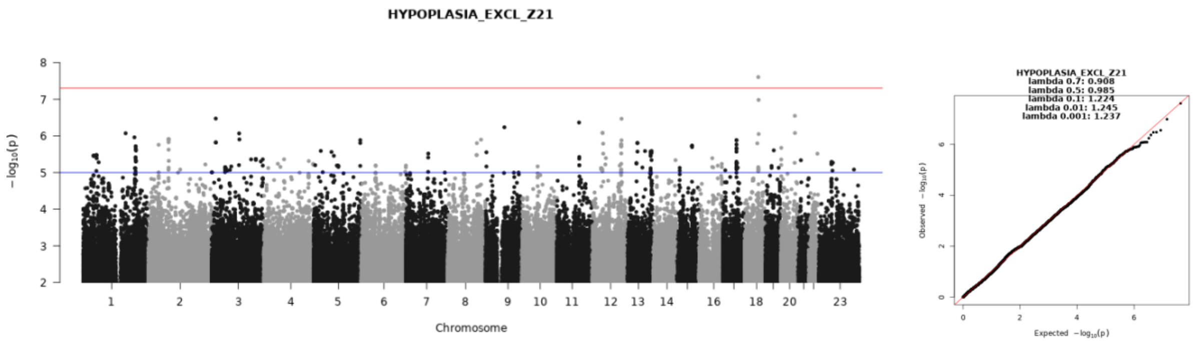

Figure S25. Manhattan and QQ-plot of phenotype Hypoplasia of dental enamel.

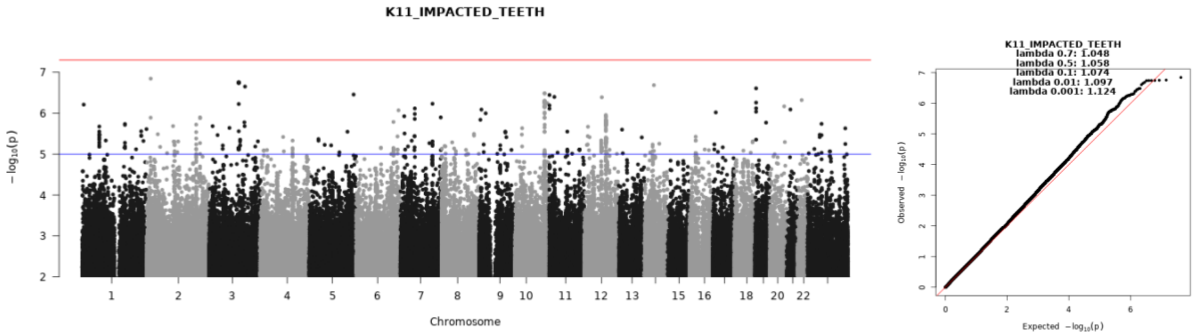

Figure S26. Manhattan and QQ-plot of phenotype Impacted teeth.

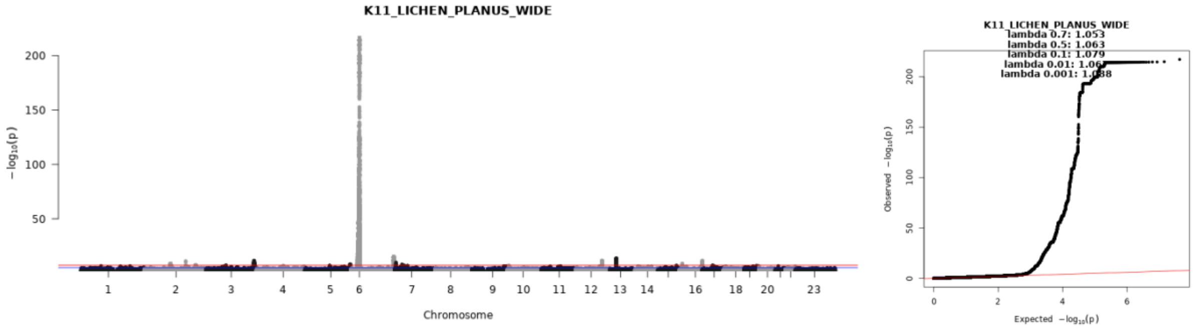

Figure S27. Manhattan and QQ-plot of phenotype Peripheral and oral lichen planus.

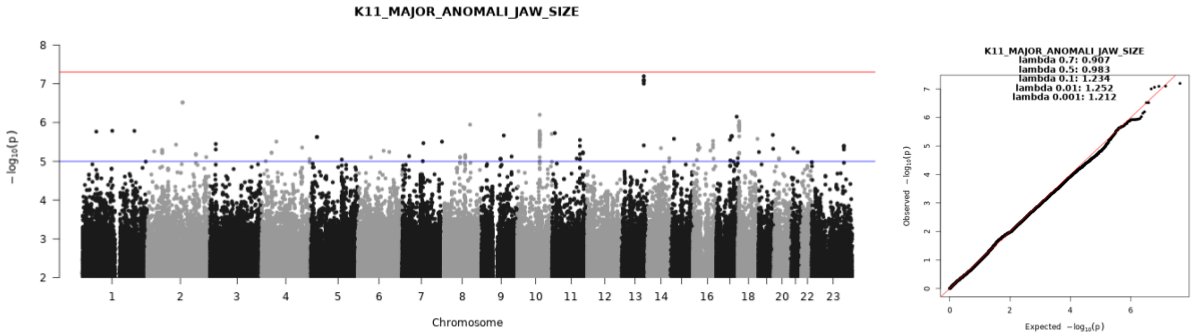

Figure S28. Manhattan and QQ-plot of phenotype Major anomalies of jaw size.

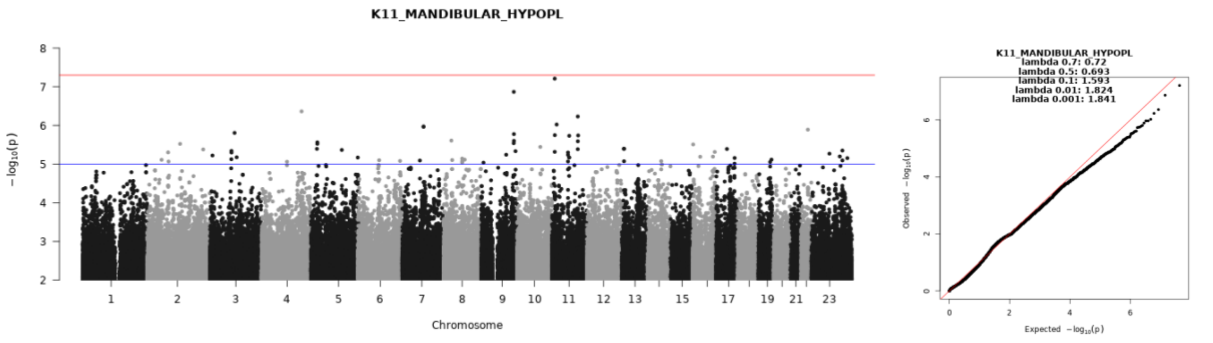

Figure S29. Manhattan and QQ-plot of phenotype Mandibular hypoplasia.

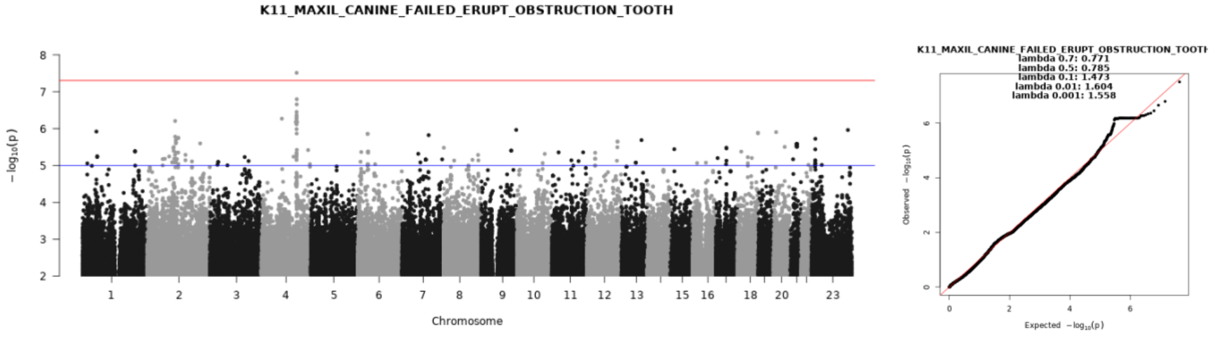

Figure S30. Manhattan and QQ-plot of phenotype Impacted maxillary canine.

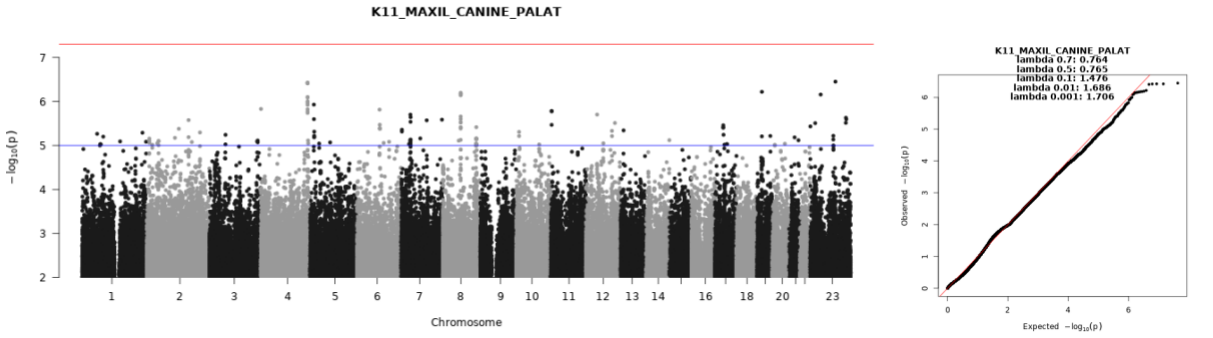

Figure S31. Manhattan and QQ-plot of phenotype Palatinal maxillary canine.

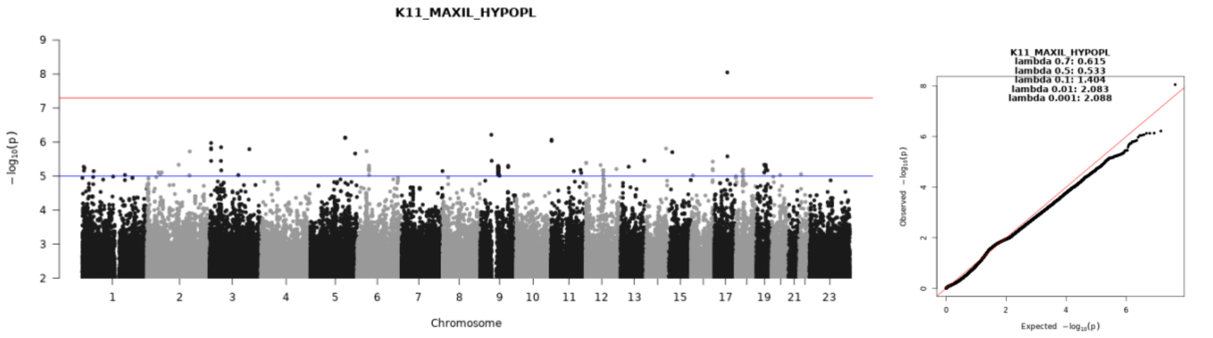

Figure S32. Manhattan and QQ-plot of phenotype Maxillary hypoplasia.

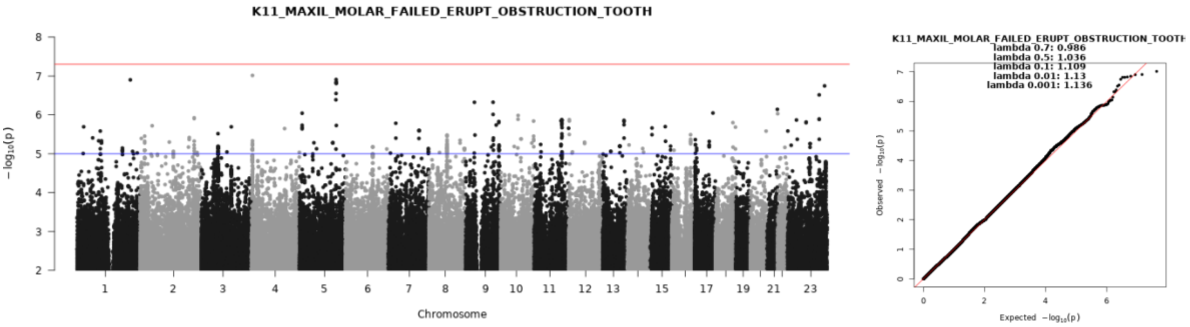

Figure S33. Manhattan and QQ-plot of phenotype Impacted maxillary molar.

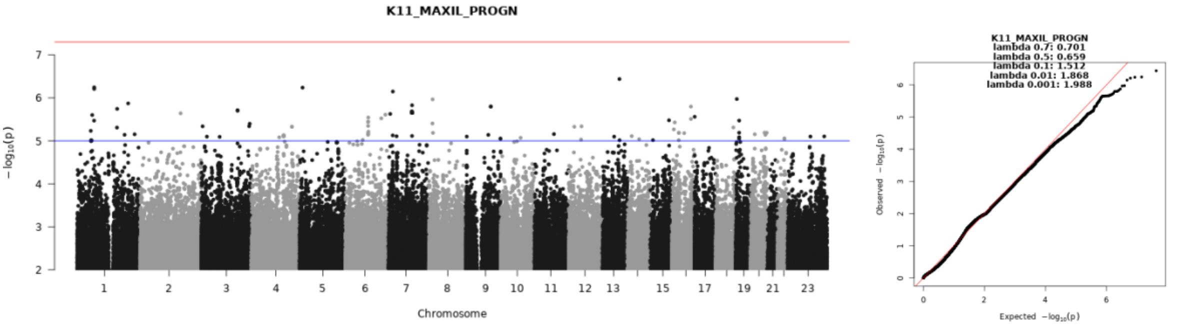

Figure S34. Manhattan and QQ-plot of phenotype Maxillary prognathia.

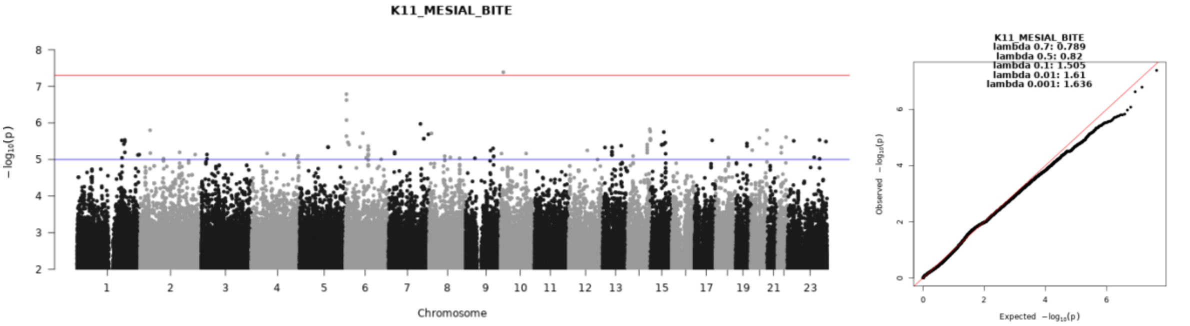

Figure S35. Manhattan and QQ-plot of phenotype Mesio-occlusion.

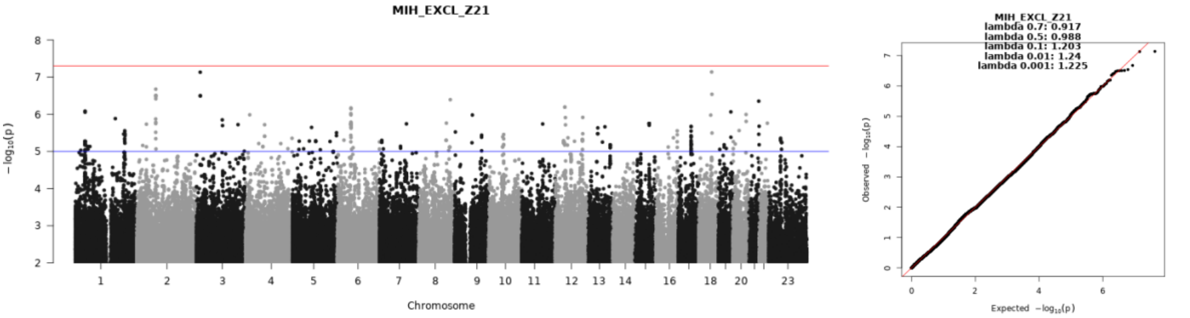

Figure S36. Manhattan and QQ-plot of phenotype Molar incisor hypomineralisation (MIH).

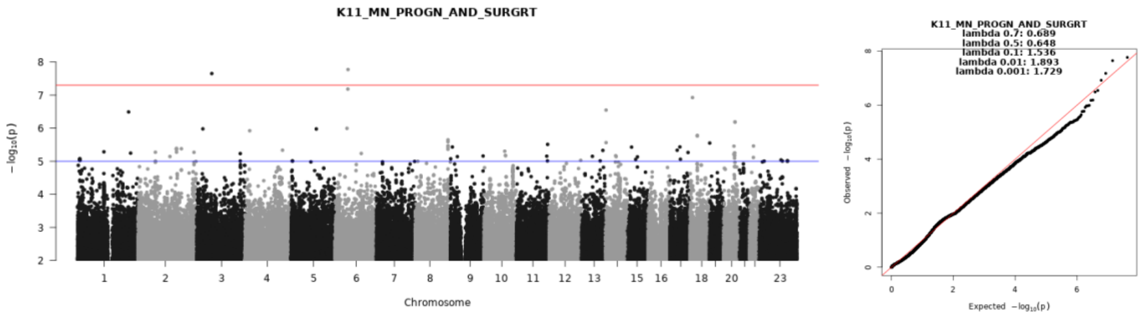

Figure S37. Manhattan and QQ-plot of phenotype Mandibular prognathia and surgery (LeFort I or BSSRO).

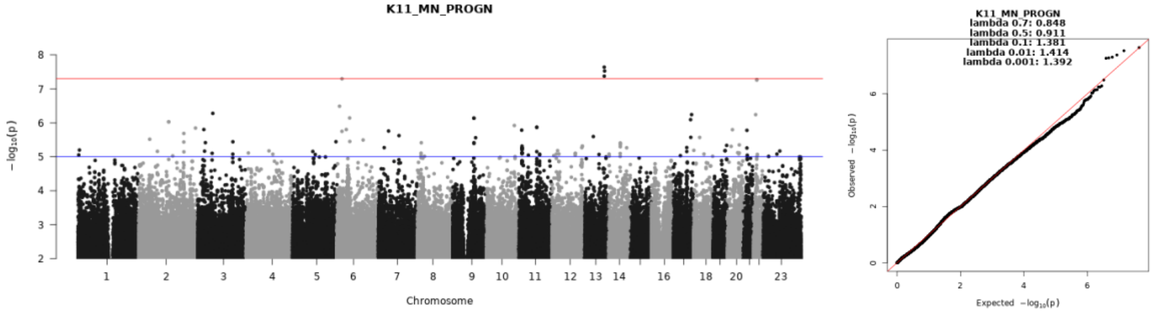

Figure S38. Manhattan and QQ-plot of phenotype Mandibular prognathia.

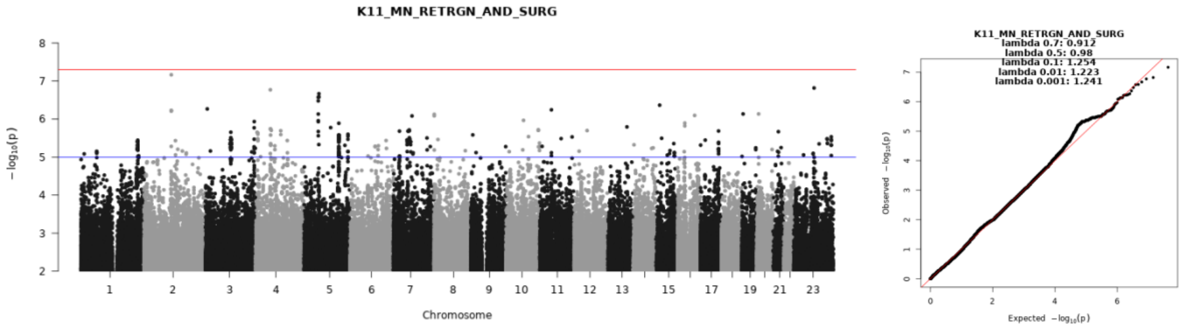

Figure S39. Manhattan and QQ-plot of phenotype Mandibular retrognathia and surgery (BSSRO).

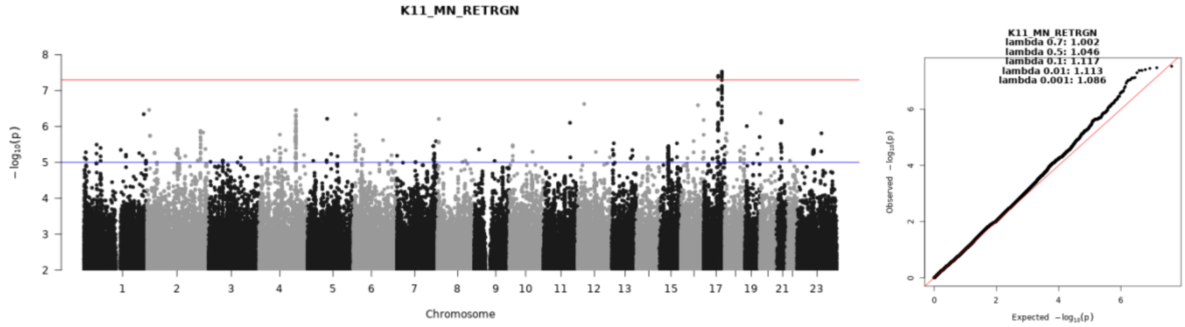

Figure S40. Manhattan and QQ-plot of phenotype Mandibular retrognathia.

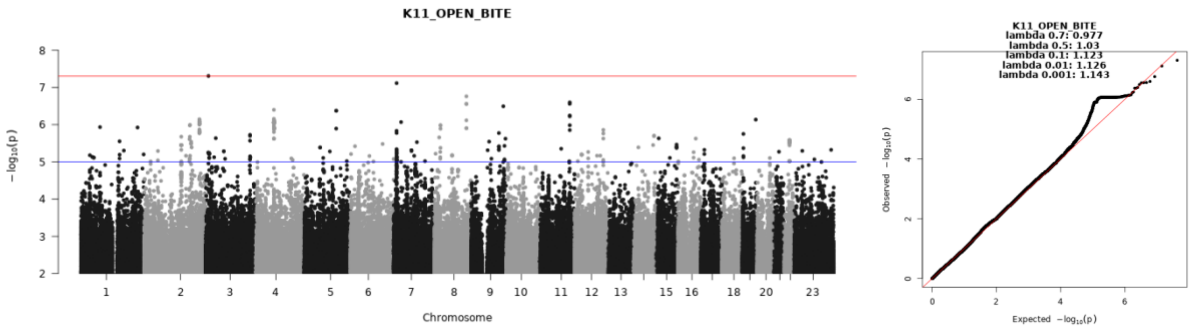

Figure S41. Manhattan and QQ-plot of phenotype Open bite.

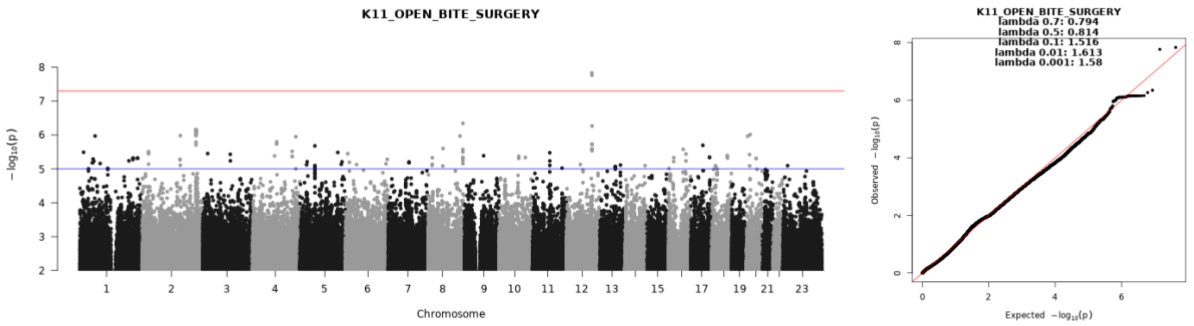

Figure S42. Manhattan and QQ-plot of phenotype Open bite that required surgery (LeFort1 or BSSRO).

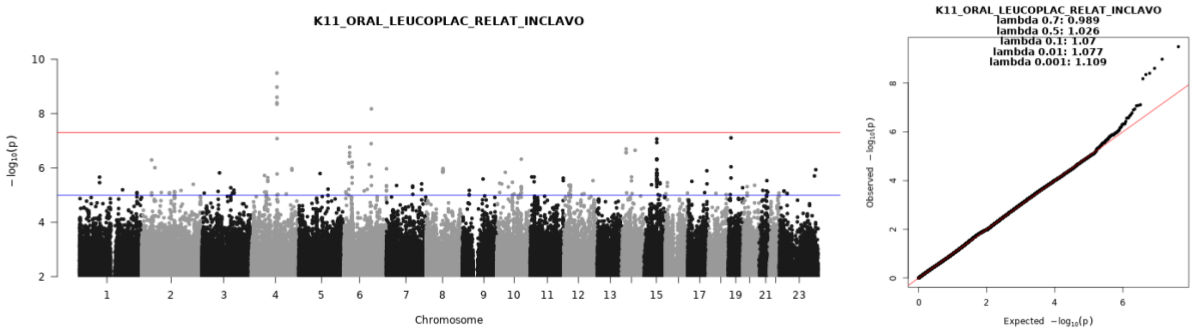

Figure S43. Manhattan and QQ-plot of phenotype Oral leukoplakia and related diseases.

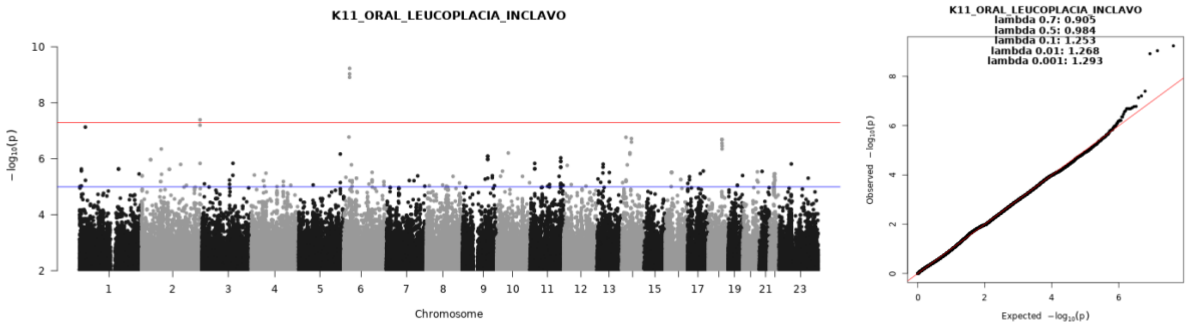

Figure S44. Manhattan and QQ-plot of phenotype Oral leukoplakia.

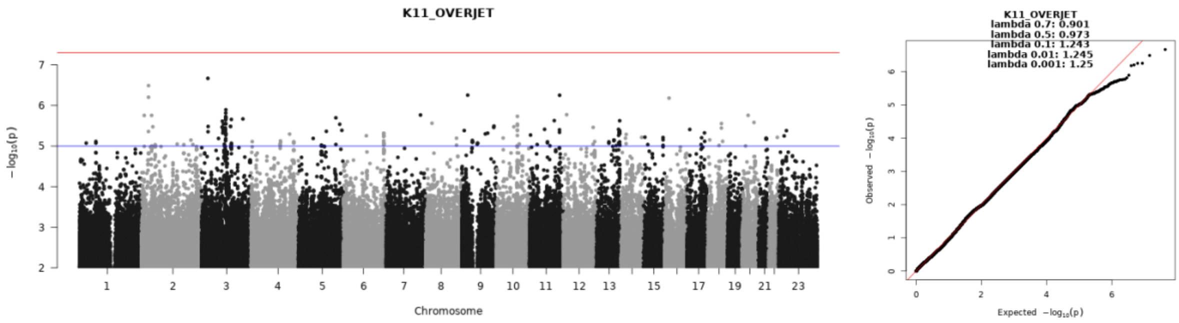

Figure S45. Manhattan and QQ-plot of phenotype Overjet.

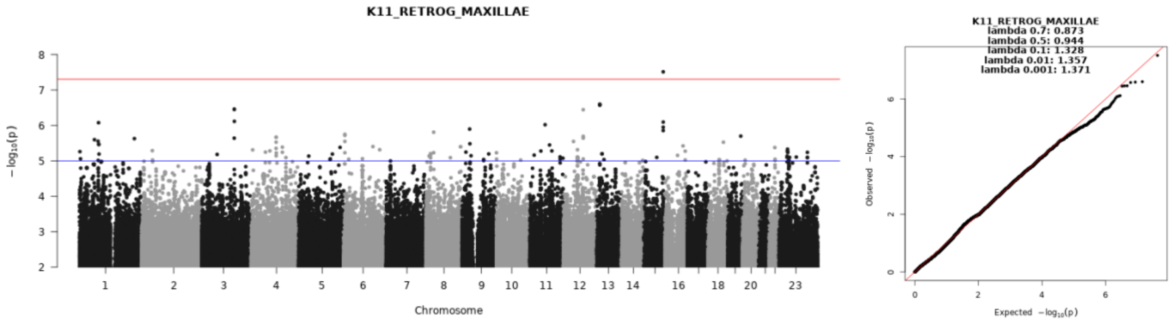

Figure S46. Manhattan and QQ-plot of phenotype Maxillary retrognathism.

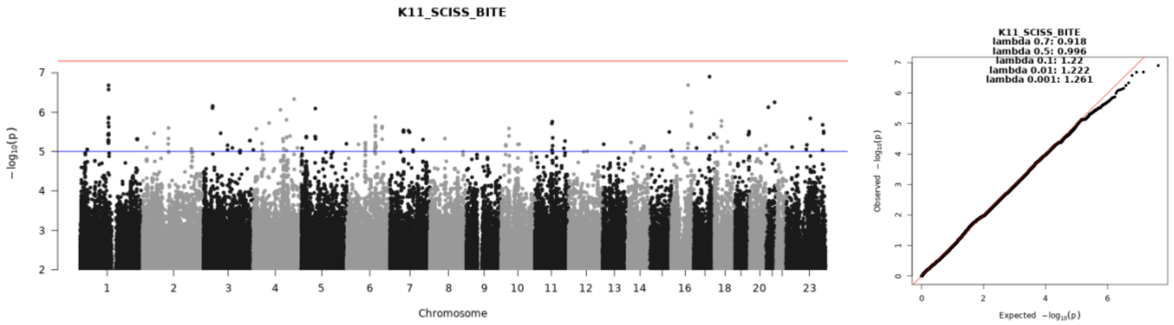

Figure S47. Manhattan and QQ-plot of phenotype Scissor bite.

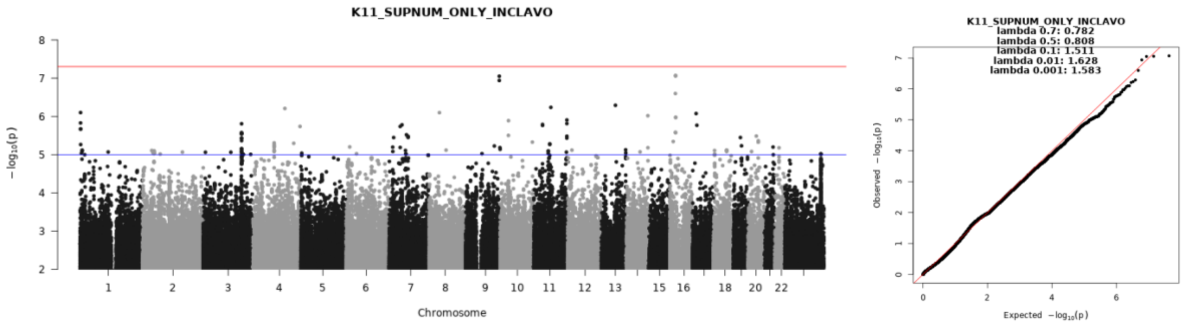

Figure S48. Manhattan and QQ-plot of phenotype Supernumerary teeth (excluding clefts and syndromes).

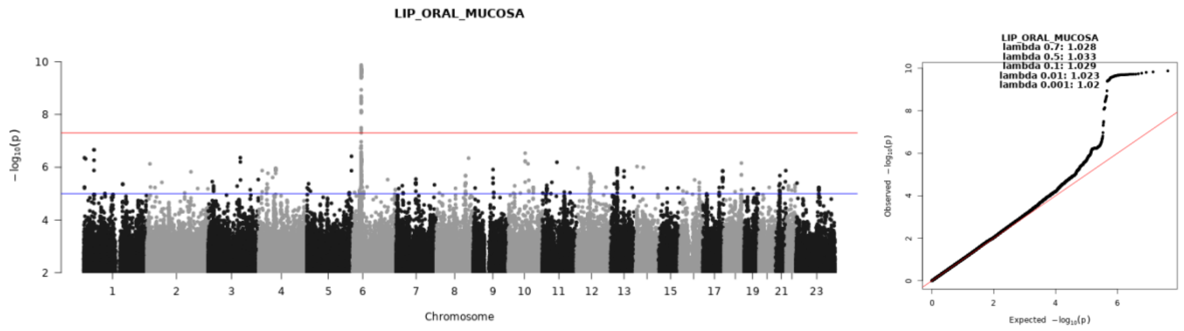

Figure S49. Manhattan and QQ-plot of phenotype Diseases of lip and oral mucosa.

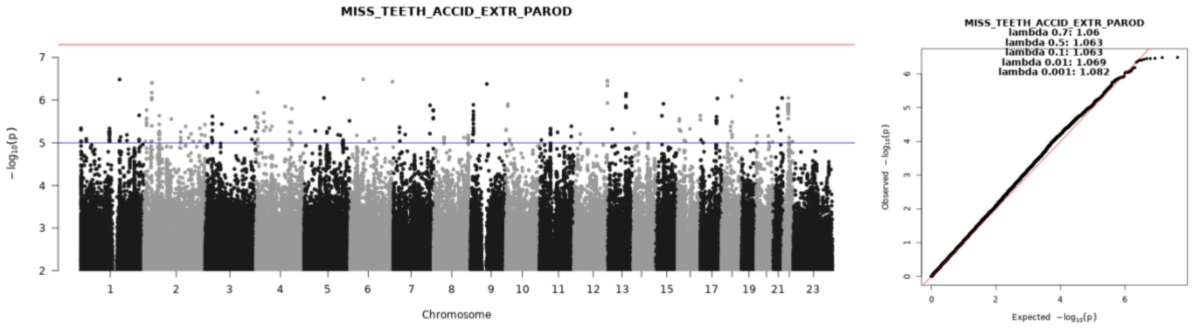

Figure S50. Manhattan and QQ-plot of phenotype Tooth loss.

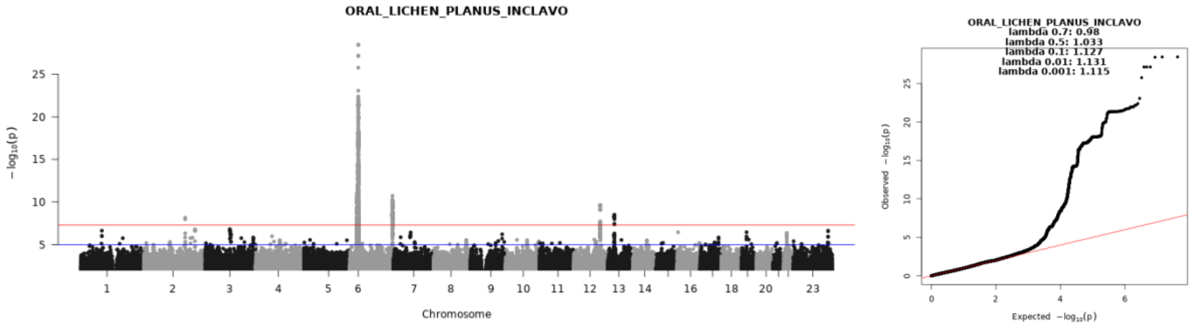

Figure S51. Manhattan and QQ-plot of phenotype Oral lichen planus.

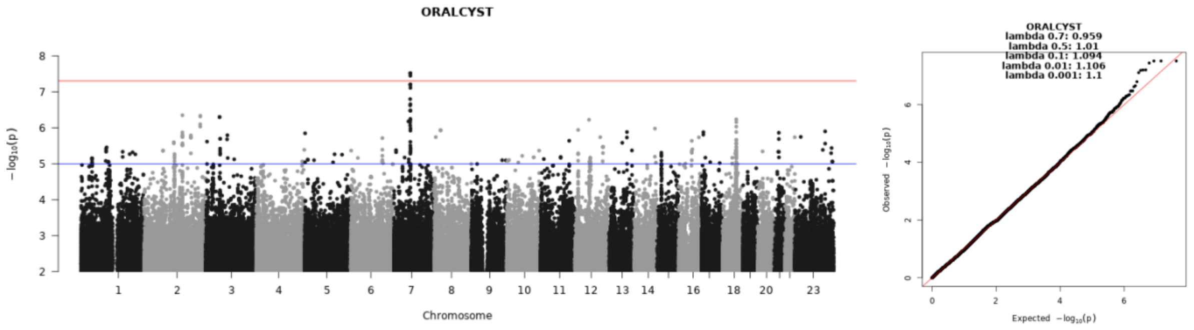

Figure S52. Manhattan and QQ-plot of phenotype Oral cysts.

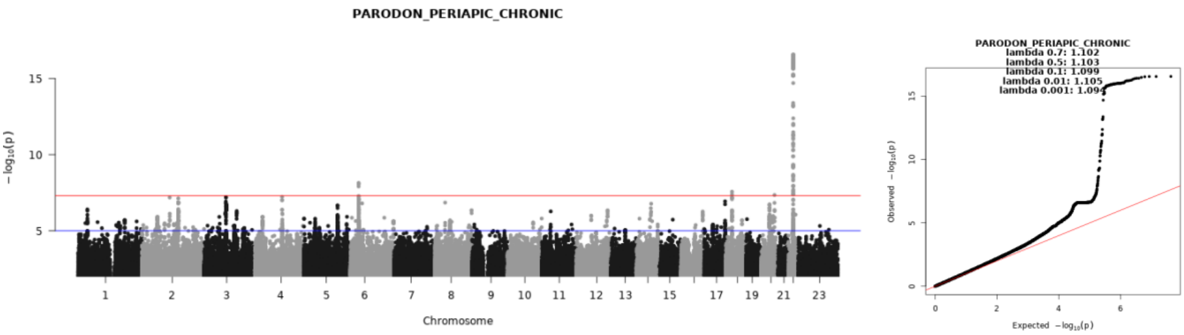

Figure S53. Manhattan and QQ-plot of phenotype Chronic apical periodontitis.

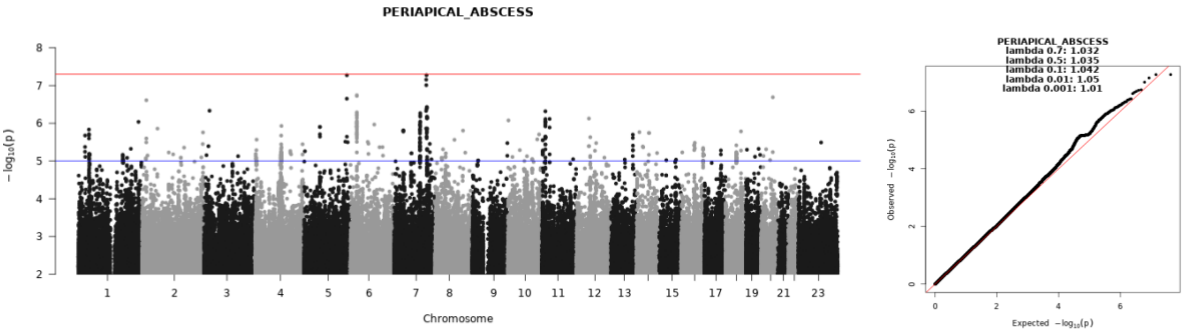

Figure S54. Manhattan and QQ-plot of phenotype Periapical abscess.

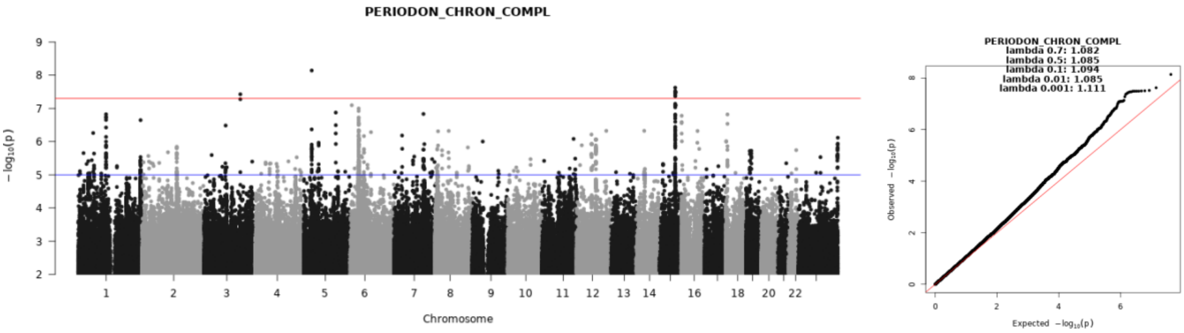

Figure S55. Manhattan and QQ-plot of phenotype Chronic complicated periodontitis.

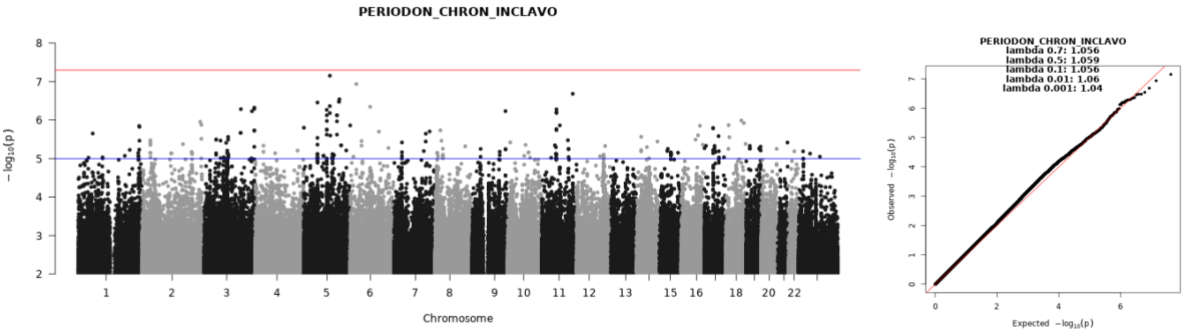

Figure S56. Manhattan and QQ-plot of phenotype Chronic periodontitis.

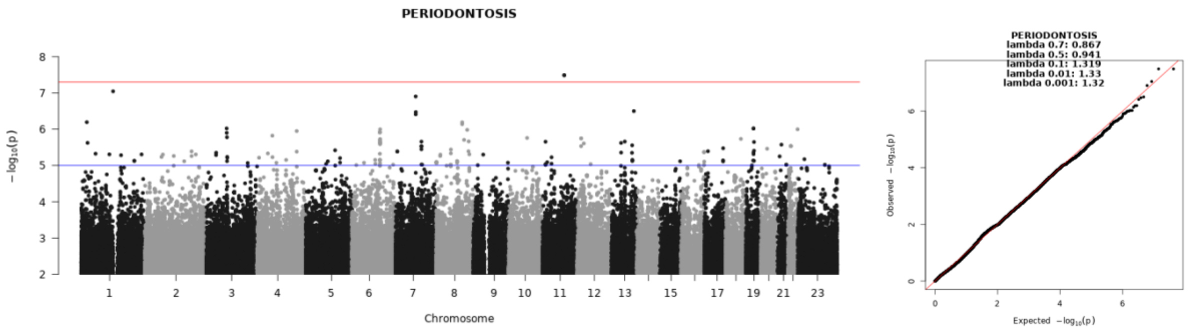

Figure S57. Manhattan and QQ-plot of phenotype Aggressive periodontitis.

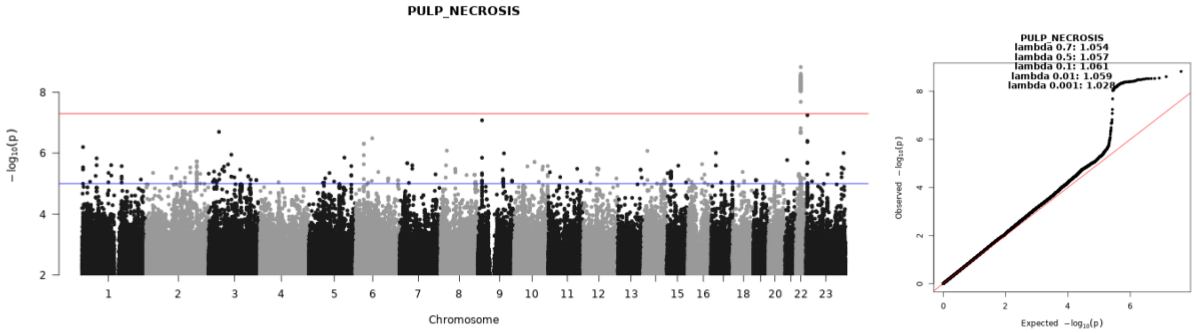

Figure S58. Manhattan and QQ-plot of phenotype Necrosis of pulp.

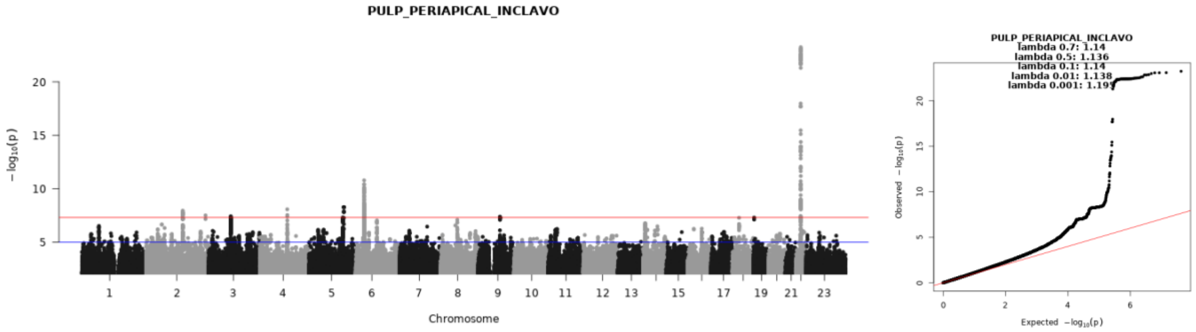

Figure S59. Manhattan and QQ-plot of phenotype Diseases of pulp and periapical tissues.

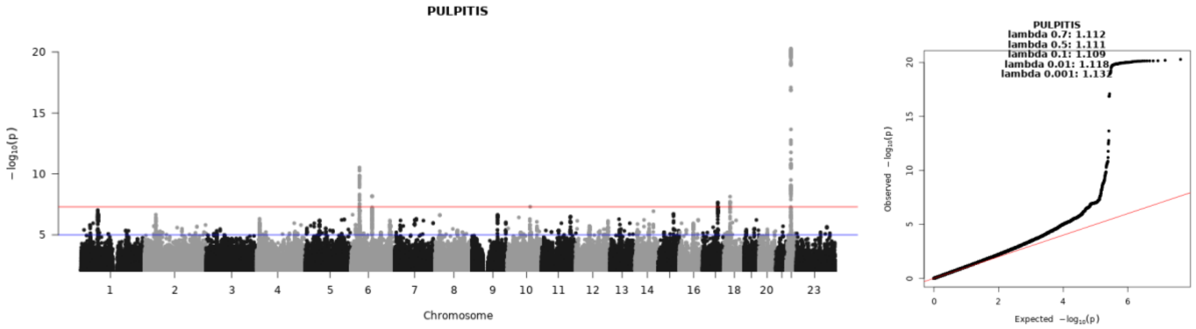

Figure S60. Manhattan and QQ-plot of phenotype Dental pulpitis.

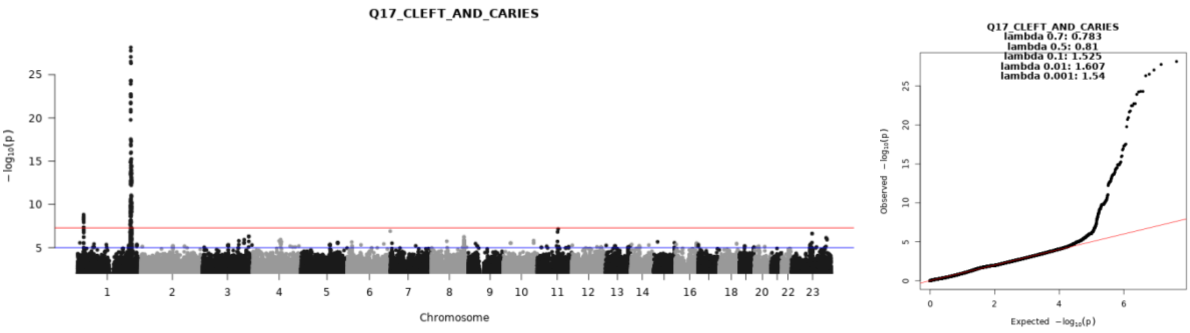

Figure S61. Manhattan and QQ-plot of phenotype Caries in clefts patients.

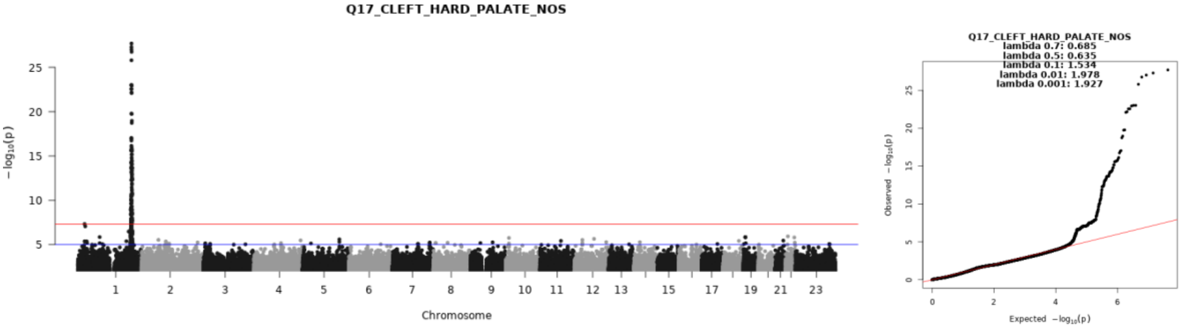

Figure S62. Manhattan and QQ-plot of phenotype Cleft hard palate.

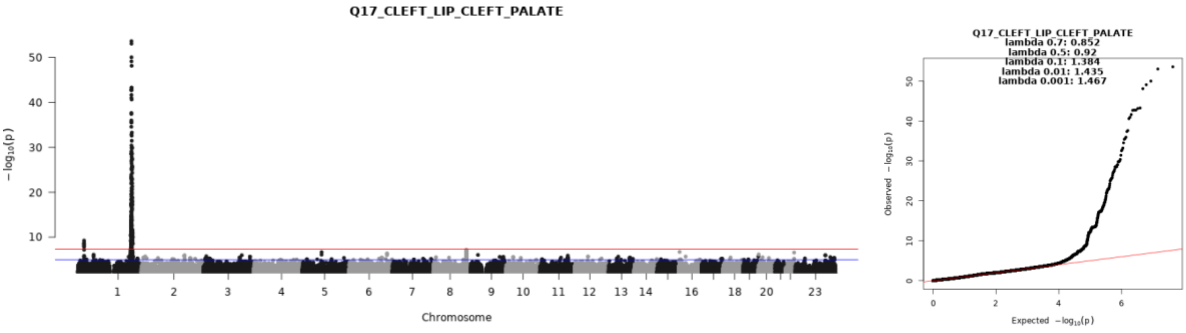

Figure S63. Manhattan and QQ-plot of phenotype Cleft lip, lip and palate or palate.

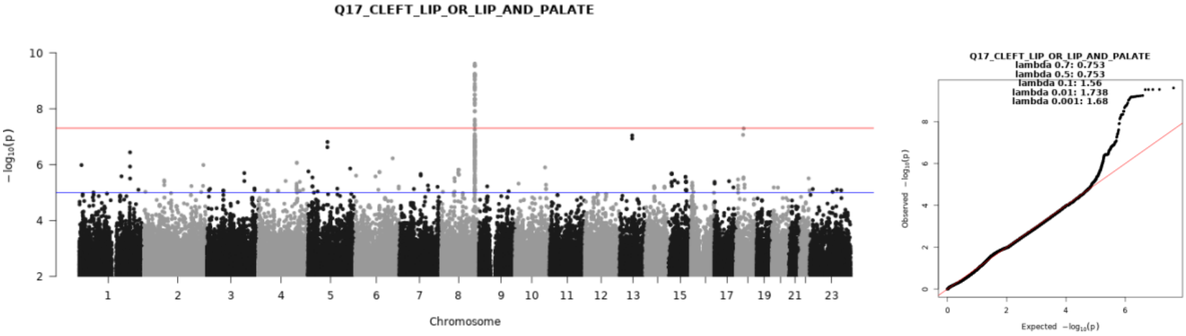

Figure S64. Manhattan and QQ-plot of phenotype Cleft lip or cleft lip and palate.

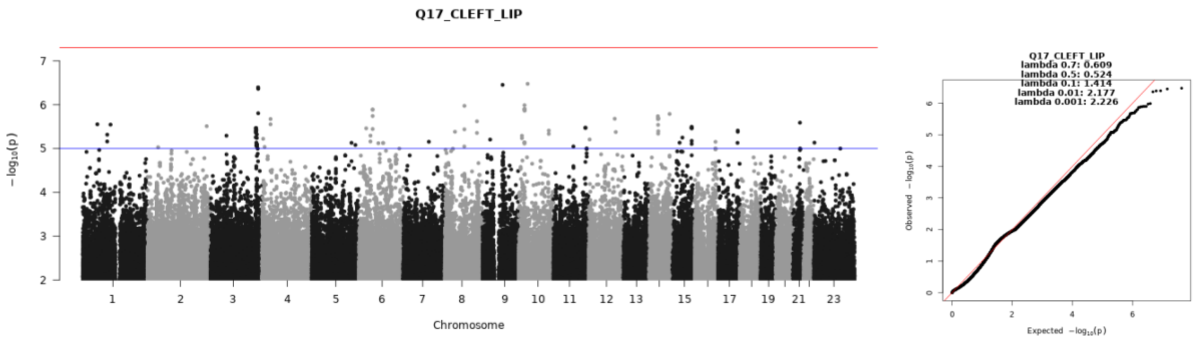

Figure S65. Manhattan and QQ-plot of phenotype Cleft lip.

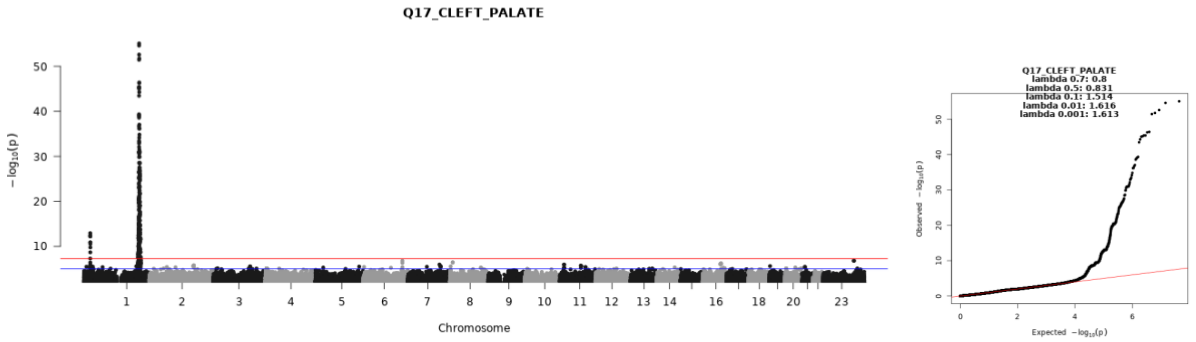

Figure S66. Manhattan and QQ-plot of phenotype Cleft palate.

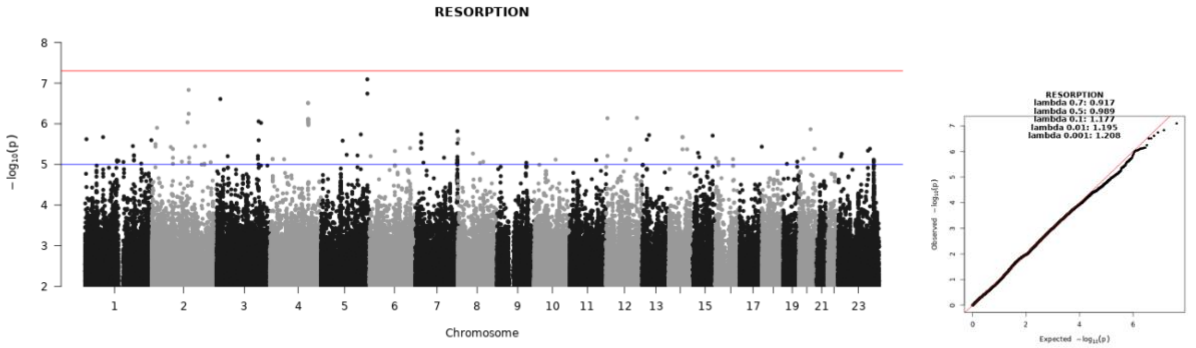

Figure S67. Manhattan and QQ-plot of phenotype Pathological resorption of teeth.

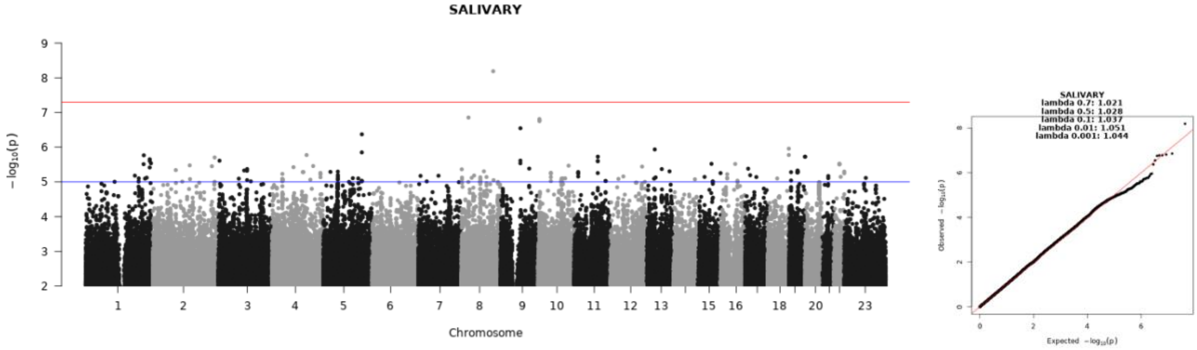

Figure S68. Manhattan and QQ-plot of phenotype Diseases of salivary glands.

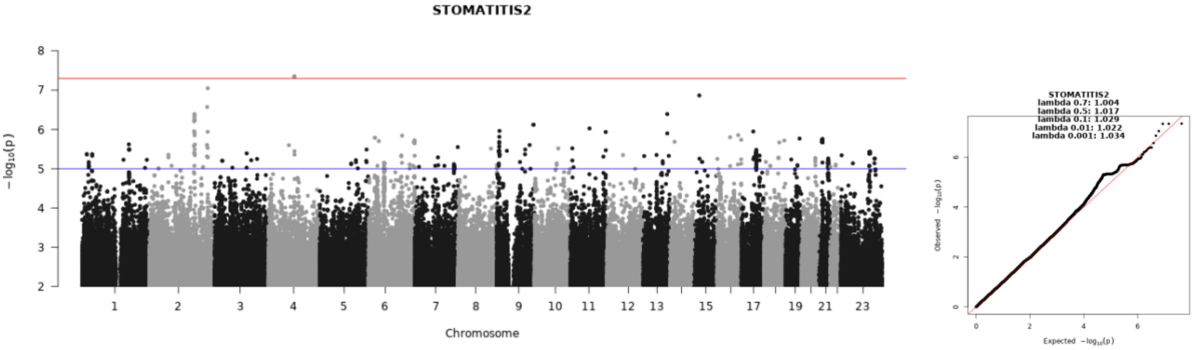

Figure S69. Manhattan and QQ-plot of phenotype Stomatitis and related lesions.

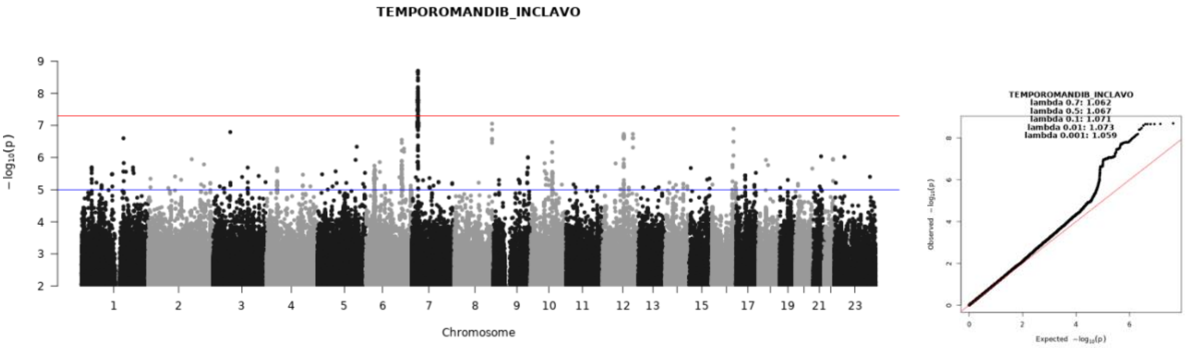

Figure S70. Manhattan and QQ-plot of phenotype Temporomandibular joint disorders.

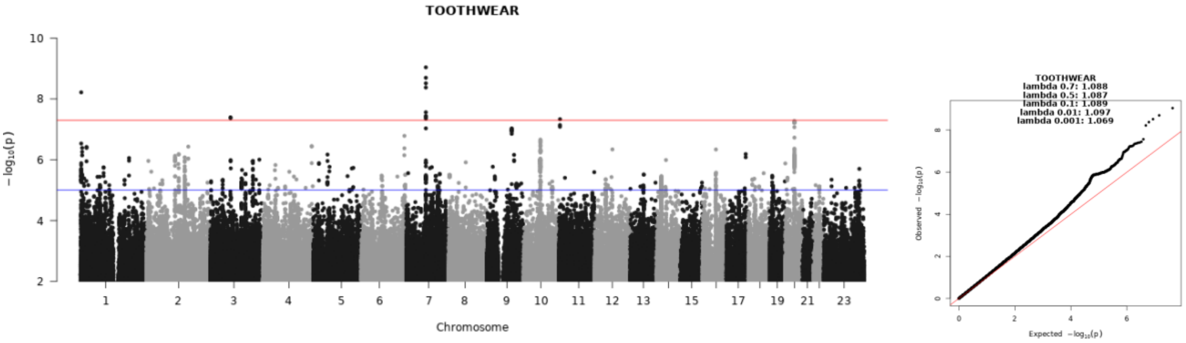

Figure S71. Manhattan and QQ-plot of phenotype Tooth wear.
